# University students’ use of mental health services: a systematic review and meta-analysis

**DOI:** 10.1186/s13033-022-00569-0

**Published:** 2022-12-17

**Authors:** T. G. Osborn, S. Li, R. Saunders, P. Fonagy

**Affiliations:** 1 Division of Psychology and Language Sciences, Faculty of Brain Sciences, UCL, 26 Bedford Way, London, WC1H 0AP UK; 2 Centre for Outcomes Research and Effectiveness (CORE), Research Department of Clinical Educational and Health Psychology, University College London, 1-19 Torrington Place, London, WC1E 7HB UK

**Keywords:** University students, Healthcare, Utilisation, Accessibility, Mental health services, Systematic review, Meta-analysis

## Abstract

**Background:**

International estimates suggest around a third of students arrives at university with symptoms indicative of a common mental disorder, many in late adolescence at a developmentally high-risk period for the emergence of mental disorder. Universities, as settings, represent an opportunity to contribute to the improvement of population mental health. We sought to understand what is known about the management of student mental health, and asked: (1) What proportion of students use mental health services when experiencing psychological distress? (2) Does use by students differ across health service types?

**Methods:**

A systematic review was conducted following PRISMA guidelines using a Context, Condition, Population framework (CoCoPop) with a protocol preregistered on Prospero (CRD42021238273). Electronic database searches in Medline, Embase, PsycINFO, ERIC and CINAHL Plus, key authors were contacted, citation searches were conducted, and the reference list of the WHO World Mental Health International College Student Initiative (WMH-ICS) was searched. Data extraction was performed using a pre-defined framework, and quality appraisal using the Joanna Briggs Institute tool. Data were synthesised narratively and meta-analyses at both the study and estimate level.

**Results:**

7789 records were identified through the search strategies, with a total of 44 studies meeting inclusion criteria. The majority of included studies from the USA (n = 36), with remaining studies from Bangladesh, Brazil, Canada, China, Ethiopia and Italy. Overall, studies contained 123 estimates of mental health service use associated with a heterogeneous range of services, taking highly variable numbers of students across a variety of settings.

**Discussion:**

This is the first systematic quantitative survey of student mental health service use. The empirical literature to date is very limited in terms of a small number of international studies outside of the USA; studies of how services link together, and of student access. The significant variation we found in the proportions of students using services within and between studies across different settings and populations suggests the current services described in the literature are not meeting the needs of all students.

**Supplementary Information:**

The online version contains supplementary material available at 10.1186/s13033-022-00569-0.

## Background

Globally, university students could be considered a privileged group given the significant variation in percentage of national populations with a university education [1]. However, for those who do attend university usually do so at a developmentally high risk period for the emergence of mental heath problems [2, 3]. Psychological distress, encompassing symptoms ranging from normal fluctuations in mood to the emergence of a serious mental illness, is an increasingly common experience among university students which can have significant consequences for individuals [4, 5]. Recent international evidence suggests 35% of first year students report symptoms indicative of lifetime mental disorder, and 31.4% report symptoms in the previous 12 months [6]. International longitudinal research is more limited. Studies in Norway, the UK and the USA has shown both psychological distress and common mental disorders (CMD) have increased in prevalence among both students and similar aged non-student populations over the last 10 years [7–11]. Suicidal behaviour, while lower in students compared to matched non-student populations, has also increased over a similar timeframe in England and Wales [12]. International estimates among students suggest around 4.3% have attempted suicide in their lifetime [6]. The short- and longer-term consequences of mental health difficulties can be significant including poorer academic performance, relationship breakdown, and exclusion from the labour market [6, 13, 14]. Current students face greater financial and academic pressures compared to 20 years ago, which may be contributing to poorer mental health outcomes [2, 15–17]. These findings suggest a significant mental health need among this population. [1].

For students in mental distress, the support available to them is likely to vary signficiantly between and within countries. For example, in many high-income countries (HIC) students may have a range of effective mental health services available to them but these services are often fragmented, uncoordinated and underutilised [6, 19, 20]. For example, US studies suggest around a 1/3 of students received treatment [9], while epidemiological studies suggest this varies widely independent of need based on sex and gender, ethnicity, age, and where they attend university [6, 20–23]. Barriers such as self-stigma, perceived need, and self-reliance influence when and how they seek help, while student’s also report a lack of awareness of appropriate services, concerns about confidentiality and discrimination, cost, or may perceive services to be ineffective or inappropriate [19, 24, 25]. These barriers may explain why some students only seek help in crisis and others tend to rely on informal sources of support [26, 27]. International studies suggest very few students with need, receive support globally. One recent international cross-sectional study found 19.8% of first year university students, and 36% of those who may meet criteria for CMD report having ever used a mental health service, defined as medication or psychological counselling [6]. Compared to HICs, much less is known about students in Lower and Middle Income Countries (LMIC), although individual studies suggest very small numbers of students report accessing support when in distress [18, 28].

While a limited number of studies have highlighted the scale and nature of the problem outside of the USA, there is a renewed effort to understand and address barriers to treatment that stop some students reaching help in the first place [4, 16, 27]. The World Health Organization’s (WHO) World Mental Health International College Student Initiative (WMH-ICS) aims to provide greater clarity on the unmet need of this group [16]. In the UK, there has been a policy focus on improving access to mental health interventions through greater integration between the National Health Service (NHS) and Universities, and an emphasis on mobilising university resources towards the mental health of students [29, 30]. Previous reviews in the USA have looked at which students are most likely to seek help [20, 31], however this is obviously confounded by the nature of services available to them. There are no systematic reviews conducted on the variety of services available to students internationally, how these integrate with each other and how use varies by types of service that deliver interventions to support mental health and wellbeing. Studies have examined individual services such as university counselling centres, external psychological services, or inpatient settings but have not compared the differential use of these by students with different clinical presentations. Given the developmental period in which many students attend university these settings are important in contributing to improving overall population mental health [3, 32]. By understanding where variation occurs could indicate areas of differential access, highlighting where care pathways could be improved and inform policy initiatives.

This systematic review was conducted to address this gap, by answering two review questions: (1) what proportion of university students use mental health services when experiencing psychological distress? And (2) does utilisation differ across health service type?

## Method

This review was reported in accordance with PRISMA guidelines [33] (see Additional file 1: Appendix S1). A protocol for this review was pre-registered on the 22/02/21 on PROSPERO (https://www.crd.york.ac.uk/prospero/display_record.php?ID=CRD42021238273).

### Deviations from initial protocol

On the 26th of April 2021 we made an amendment to only include studies published in the year 2000 or after over concerns around changes to the student population that would create issues of comparability [4]. On the 27th of July 2021 we amended the focus of the review as the original aims were considered too broad for a coherent synthesis. The amendment removed one review question related to student characteristics associated with service use which could be explored in future analysis.

### Eligibility criteria

Studies were included that:

1. Measured the use or utilisation of mental health services (as a primary or secondary outcome).
2. Studies that included adults (aged 18 +) studying at a university.

Studies were excluded:

1. That employed an empirical study design that aimed to test an intervention or approach to address or effect access or use of healthcare services.
2. Where it was not possible to extract sociodemographic and utilisation data for student participants.
3. Where participants under 18 were recruited.
4. Where participants weren’t all university students.

Studies needed to be published in English due to the languages spoken by the primary reviewer (TO).

### Search strategy

The following electronic databases were searched on the 9th of March 2021, 3rd of November 2021 and the 23rd of August 2022: MEDLINE (Ovid); EMBASE (Ovid); PsycINFO (Ovid); ERIC (ESBCO); and CINAHL plus (ESBCO). The search strategy using a Context, Condition, Population (CoCoPop) framework with the concepts of “students”, “mental health/illness”, “access” and “mental health services” [34]. Key words and MeSH terms were developed in Medline between 2nd of December 2020 and 9th of March 2021, and adapted for each database (see Additional file 1: Appendix S2). On the 16th and 17th of June 2021, the 14th of December 2021 and the 16th of November 2022 forward and backward citation searching was conducted. The publicly available reference list of studies published by the WHO’s WMH-ICS was searched on the 23rd of April 2021, the 14th of December 2021 and the 16th of November 2022. The authors of the originally included studies were contacted on the 18th of June 2021, where possible, to help identify any unpublished or ongoing research.

### Data extraction

Records retrieved from electronic database searches were exported to Endnote X9, where duplicates were removed. Abstracts and full texts of potentially relevant articles were screened against the inclusion and exclusion criteria on Rayyan software. A random sample of approximately 10% of titles and abstracts identified in the initial searches were screened independently by a second reviewer (SL) using a purpose designed screening tool (see Additional file 1: Appendix S3). Data from the included studies were extracted independently by two reviewers (TO and SL) using a pre-defined data extraction framework (see Additional file 1: Appendix S4). Data were extracted into Excel. After data were extracted for two studies, the data extraction framework was checked for interpretation by both TO and SL. Study authors were contacted where additional data or clarification was required. The main items of interest were:

#### i Condition: use or utilisation

We defined use as the occurrence or number of uses of a mental health service over a defined time-period [35]. Indicators could include attendances, usage, inpatient days, admissions, contacts, episodes, or costs due to the receipt of treatment or attendance [35]. These indicators may be measured through self-report, clinical records, and/ or other routinely collected data. As observational or more naturalistic study designs were included in this review, outcomes are likely to be reported as prevalence or incidence and therefore as a proportion of the total study sample. Therefore, the effect measures were proportions with a 95% confidence interval as the main outcome [34].

#### ii Context: mental health service

An amended version of the WHO’s definition of a mental health service was used, this being ‘the means by which effective interventions are delivered for the dominant or subdominant intention to improve wellbeing or mental health’ [36]. This included outpatient services, day treatment, inpatient wards, community mental health teams, General Practice, mental health hospitals, and university counselling services [36]. To facilitate comparison of proportions by service type an adapted version of the Description and Evaluation of Services for Disabilities in Europe (DESDE) instrument was used (see Appendix S5) [37]. This is a hierarchical classification system, with six initial categories: (1) Information for care, (2) Accessibility to care, (3) Self-help and volunteer care, (4) Outpatient Care, (5) Day care, and (6) Residential care. A random 10% sample were double coded by two reviews (TO and SL). No service descriptions could be classified beyond the first level of the DESDE hierarchy. Therefore, to further specify, we used the National Institute for Health and Care Excellence (NICE) treatment stepped care categories, referred to as ‘treatment type’ [38], and the service location—being either on campus, off campus, or potentially either.

#### iii Other items

We also collected sociodemographic characteristics, study design, duration of study, data collection methods, data analysis methods, setting and date of study, raw data for the outcome, indicator(s) used, and time point(s) outcomes where reported, source of funding and conflicts of interest.

### Quality assessment

We assessed risk of bias using the Joanna Briggs Institute (JBI) appraisal checklist for systematic review reporting prevalence data [34]. The checklist prompts the reviewer to answer nine questions with four possible response options: “yes”/ “no”/ “unclear”/ “not applicable”. Each study was assigned low, moderate, or high quality based on the number of yes answers it scored to indicate study quality. Studies with 1–3 ‘yes’ were low, 3–6 indicating moderate, and 7–9 as high quality. Quality appraisal was conducted independently on all studies meeting the inclusion criteria by two reviewers (TO and SL). Where there were disagreements, these were discussed until agreement was reached. No studies were excluded based on the study quality to enable sensitivity analyses to be conducted by removing studies rated as low quality.

### Synthesis methods

#### i Narrative synthesis

Initially, a non-statistical narrative synthesis was conducted to describe the included studies relevant to the review questions [34]. Study participants and the measures of psychological symptoms were not universally well described. Therefore, the samples were qualitatively summarised and then categorised based on whether this was a general student sample, subgroup sample or a sample of students with more severe current psychological distress, referred to as ‘at risk’.

#### ii Meta-analysis

Most studies provided data for multiple service types, therefore three-level mixed effects models were used to account for clustering. Where the study provided a single estimate or an overall estimate of service use they were included in one of three conventional random effects meta-analytic models: (1) overall service use (any service), (2) overall outpatient service use, (3) overall residential service use reflecting the service types commonly observed in the data. Following this, to specifically test differences between these service types all estimates were then included into a three-level mixed effects model, where sub-group analysis and meta-regression were also conducted [39]. Further analyses were conducted for studies providing multiple estimates within the same study using two three-level mixed effects models to account for clustering: (1) outpatient service use; (2) service use where the service could be classed within multiple DESDE service categories.

For all pooled proportions, a priori subgroup analysis and meta-regression were conducted based on population group. Post-hoc analyses were conducted based on service location, treatment type, reporting timeframes, publication year, study design, and country, due to the substantial estimated heterogeneity. To conduct meta-regression for recall time-period a continuous variable was created based on the number of months participants were asked to recall service use (e.g., 12 months). If the reporting time-period did not use months (e.g., the student’s lifetime), it was estimated using the average age of the participants.

Heterogeneity was further explored by identifying outliers above or below the 95% confidence interval of the pooled proportion; by conducting influencer analysis; drafting a Baujat plot and conducting Graphic Display of Heterogeneity (GOSH) plots [39].

Sensitivity analyses were conducted for pooled estimates where low quality studies, estimates of lifetime service use and outliers and influential cases were excluded then all described analyses were repeated. Publication bias was not assessed due to the substantial between study heterogeneity [39].

## Results

### Search results

**Fig. 1:**
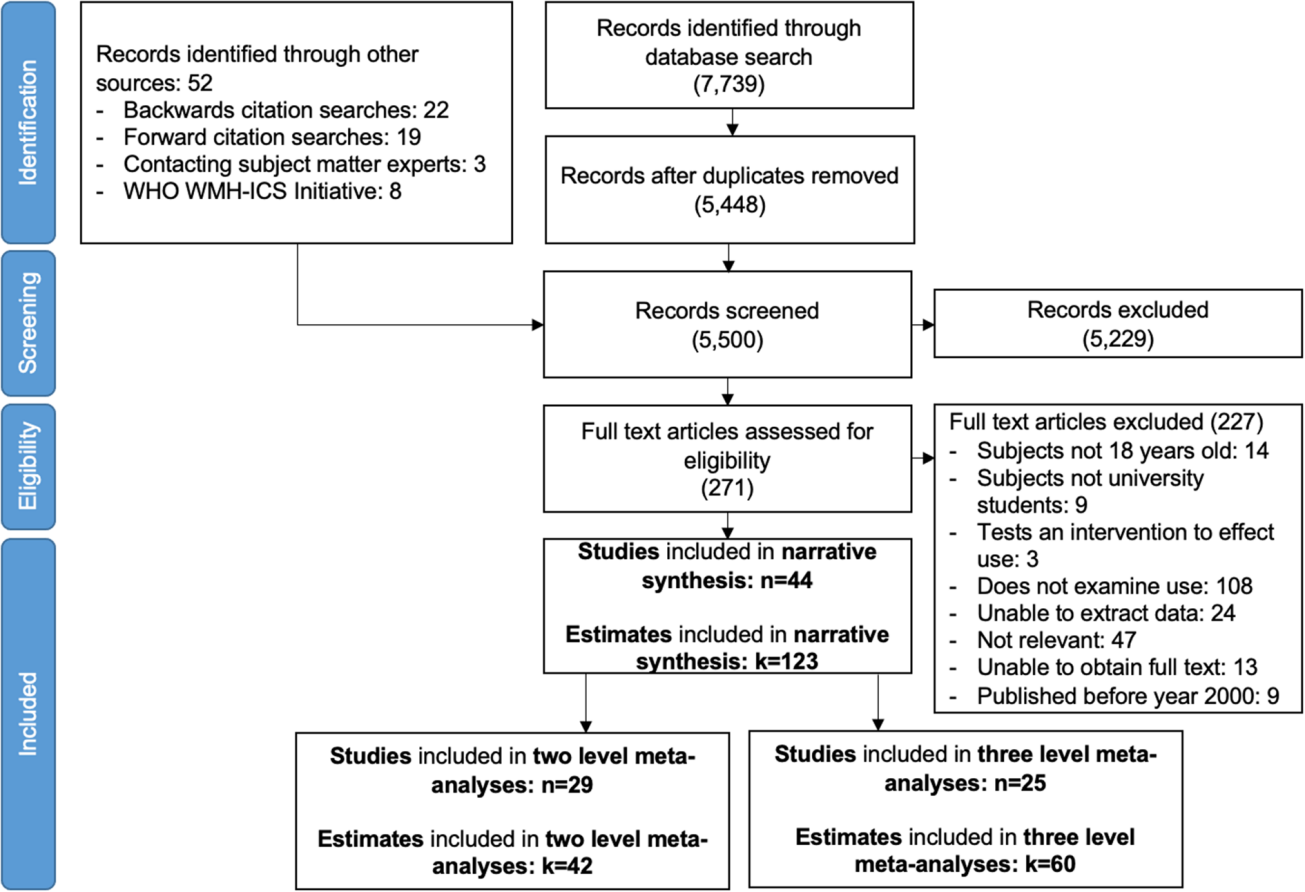
PRISMA flow diagram

A total of 7739 unique titles / abstracts were identified through database searches, and a further 52 through other search strategies (see Fig. 1 and Additional file 1: Appendix S6). Inter-rater agreement for data screening was Cohen’s Kappa (*K*) = 0.85 indicating strong agreement [40].

As a result of these search strategies, 44 studies were deemed eligible for inclusion. Within these studies there were 123 estimates of service use. Seven of these studies were smaller analyses of larger surveys conducted in the USA [23, 41–46]. These seven studies were excluded from meta-analysis as their estimates would double count participants. 29 studies and 42 estimates were included in conventional two-level meta-analyses pooling estimates of overall service use, and then a three-level meta-analysis to test differences by service type. 25 studies and 60 estimates were included in further analyses using three-level meta-analysis. Inter-rater agreement for data extraction was *K* = 0.82 indicating strong agreement [40].

### Study characteristics

#### i Study origin

Studies were conducted in a range of mostly high-income countries. The majority were from the United States, where 34 of the 44 studies were based [9, 23, 41–72]. The remainder from Australia [73, 74], Brazil [75, 76], China [77], Canada [78], Ethiopia [79], Bangladesh [28], and Italy [80]. A total of nineteen studies were samples of students from separate individual universities [43, 46, 48–55, 67, 68, 70, 73, 75–77, 79, 80]. Whereas the remaining twenty-four were samples across multiple universities [9, 20, 23, 28, 41, 44, 45, 47, 56–59, 61–66, 69, 71, 72, 74, 78].

#### ii Study design and methods

Most studies (n = 36) were either primary or secondary analyses of cross-sectional surveys [9, 20, 23, 41, 43–45, 47, 49–51, 53–56, 58, 61–69, 73–75, 78, 79] (see Table 1). Outcomes were assessed using standardised questionnaires and open questions. Of the remaining seven studies, one was a longitudinal study [46], one was a cohort study using a mix of a baseline survey and linked electronic medical records from the university counselling centre [77], two were secondary data analyses of electronic medical records from university counselling or health centres [52, 59, 60], and two were mixed method studies [48, 80].

**Table 1:** Study characteristics

Study #	Author (year)	Study type	Country	Sample size	Population group	Sample description	Outcomes assessed	Quality rating
1	Bastos, et al. (2022) [76]	Cross-sectional	Brazil	382	Subgroup	A sample of medical students at a federal university in the South of Brazil sampled between November 2019 and February 2020	- Symptoms of anxiety - Symptoms of depression - Alcohol use - Academic environment - Current mental health service use	Low
2	Huang et al. (2020) [48]	Mixed method	USA	15	At risk	Sample of all students who had been fostered, adopted, or had experience homelessness with a mental disorder diagnosis in a Miami university in Florida in 2016	- Mental disorder diagnoses - Academic functioning - Current mental health service use	Moderate
3	Gebreegziabher et al. (2019) [79]	Cross-sectional	Ethiopia	444	At risk	A multistage random sample of all students attending university in Jimma, Ethiopia in November 2012. Students who were included in the analyses of mental health service use were those who were at risk of a common mental disorder	- Mental health literacy - Substance use - Somatic symptoms - Common mental disorders - Help-seeking behaviour in previous two weeks	High
4	Jennings et al. (2015) [49]	Cross-sectional	USA	246	At risk	A convenience sample of undergraduate students in a research participant pool in a university in Southeast USA. Students included in the analysis of mental health service use had major depressive disorder	- Attitudes toward treatment seeking - Perceived stigma to treatment seeking - Self-stigma to treatment seeking - Self-reliance - Self-reported current mental health problems, - Symptoms of depression - Alcohol use - Mental health service use in the previous two months	Low
5	Cranford et al. (2008) [43]	Cross-sectional	USA	2843	General	A random sample of all students enrolled at a university in the Midwest of the USA in 2005	- Substance use behaviours - Symptoms of psychological distress - Perceived need - Mental health service use in the past 12 months	High
6	Eisenberg et al. (2007) [67]	Cross-sectional	USA	2785	General	A random sample of students registered at university in the Midwest of the USA in 2005	- Symptoms of depression and anxiety - Academic functioning - Awareness of services - Beliefs about treatment effectiveness - Perceived need in the previous 12 months - Receipt of treatment in the previous 12 months	High
7	Eisenberg et al. (2011) [46]	Longitudinal	USA	2822	General	A random sample of students registered at university in in the Midwest of USA in 2005, followed up in 2007	- Eating disorder symptoms - Symptoms of depression - Symptoms of panic disorder and anxiety - Mental health diagnoses - Suicidal ideation - Mental health service use in the last 12 months in 2005 and 24 months in 2007	High
8	Jardon et al. (2022) [70]	Cross-sectional	USA	174	Subgroup	A sample of all nursing students with health insurance, eligible for campus services at a university in Los Angeles, USA in 2021	- COVID-19 experiences - Symptoms of depression - Symptoms of anxiety - Traumatic stress - Loneliness - Resilience - Mental health service utilisation in the last 12 months	Moderate
9	Lee et al. (2021) [50]	Cross-sectional	USA	1236	At risk	A sample of all students enrolled at a public research university in Kentucky between March and April 2020. Use of mental health services were only reported in those with moderate to severe stress, anxiety, or depression	- Symptoms of stress - Symptoms of anxiety - Symptoms of depression - Mental health service use in the previous academic year on or off campus	Moderate
10	Chang et al. (2013) [51]	Cross-sectional	USA	336	Subgroup	A sample of all 1^st^, 2^nd^, and 3^rd^ year medical students at Baylor medical school in March and April 2010	- Symptoms of burnout - Symptoms of depression - Mental health service use while at medical school	Moderate
11	Williams et al. (2021) [68]	Cross-sectional	USA	152	At risk	A sample of Black men and women, and White men attending at Mid-Atlantic university in the USA who reported depressive symptoms above the 75^th^ quintile on the Symptom Checklist-90	- Symptoms of anxiety and depression - Campus service utilization	Moderate
12	Nilsson et al. (2004) [52]	Secondary data analysis	USA	2050	Subgroup	A sample of international students who had used the university counselling centre at a University in the East of the USA	- Overall functioning - Assessment at end of treatment - Use of the university counselling centre while at university	Low
13	Ryan et al. (2017) [73]	Cross-sectional	Australia	176	At risk	A sample of all 4^th^, 5^th^, and 6^th^ year medical students reporting a mental health problem at a medical school in Western Australia	- Self-reported psychological wellbeing, mental health, and stress - Perceived usefulness of health service - Barriers to service use - Use of health services in previous 12 months	Moderate
14	Smith et al. (2021) [53]	Cross-sectional	USA	292	General	A random sample of English-speaking students with an email address aged between 18 to 40 attending a university in the Midwest of the USA	- Loneliness - Social support - Basic psychological needs - Mental health service use while at university - Barriers to help seeking	Moderate
15	Yorgason et al. (2008) [54]	Cross-sectional	USA	266	General	A random sample of students registered at a university in the East of the USA	- Mental health functioning - Knowledge of and use of the university’s mental health service	Low
16	Liu et al. (2017) [77]	Cohort	China	13,085	General	A sample of all freshman university students followed up for four years noting episodes of use of the university counselling centre using linked medical records at a University in Beijing	- Symptoms of mental health problems - Help-seeking behaviours while at university	Moderate
17	Leao et al. (2011) [75]	Cross-sectional	Brazil	156	Subgroup	A sample of final year medical students attending a final year practical exam at a medical school in Sao Paulo	- Depression and anxiety symptoms - Perceived need for help - Knowledge of university mental health service - Use of university mental health service	Moderate
18	Giusti et al. (2020) [80]	Mixed methods	Italy	103	At risk	A random of all students who used an online mental health service at a university in the Abruzzo region	- Traumatic distress - Anxiety and depression symptoms - Current treatment - Lifetime mental health service use	Moderate
19	Bourdon et al. (2020) [55]	Cross-sectional	USA	674	General	A random sample of undergraduates attending Virginia Commonwealth University in 2018	- Mental health concerns - Treatment received for mental health concerns - Lifetime mental health service use	Moderate
*Multiple institution samples*								
20	Dyrbye et al. (2015) [56]	Cross-sectional	USA	154	At risk	A sample of all 2^nd^,3^rd^, and 4^th^ year medical students with burnout attending Mayo Medical School; University of Washington School of Medicine; University of Alabama School of Medicine; University of California, San Diego School of Medicine; Rutgers New Jersey Medical School; and Uniformed Services University of Health Sciences in 2012	- Symptoms of burnout - Symptoms of Depression - Quality of life - Help-seeking behaviours in the 12 months	High
21	Eisenberg et al. (2011) [45]	Cross-sectional	USA	14,175	General	A convenience sample of 13 universities in 2007 and then 15 universities in 2009 the USA. A random sample of students registered in each institution were sampled in 2007 and 2009	- Symptoms of depression - Symptoms of panic and anxiety - Suicidal ideation - Self-injurious behaviours - Mental health service use in the last 12 months	High
22	Eisenberg et al. (2012) [20]	Cross-sectional	USA	8488	General	A convenience sample of 15 universities in the USA. A random sample of students registered in each institution were sampled in 2009	- Symptoms of depression - Suicidal thoughts and behaviours - Symptoms of anxiety - Substance use - Minimally adequate care - Mental health service use in the last 12 months	Moderate
23	Fischbein et al. (2019) [44]	Cross-sectional	USA	482	Subgroup	A convenience sample of 23 universities in the USA. A random sample of medical and pharmacy students registered in each institution were sampled between 2015–2016	- Symptoms of depression - Symptoms of anxiety - Use of alcohol and other substance - Perception of treatment efficacy - Stigma - Receipt of medication in the last 12 months - Formal and informal help-seeking in the last 12 months	High
24	Han et al. (2016) [57]	Cross-sectional study	USA	9400	At risk	A nationally representative sample of all adults with a household address in the USA sampled between 2008 and 2016. Students those who reported suicidal thoughts and behaviour	- Suicidal thoughts and behaviour - Mental and physical health status - Mental health treatment in the last 12 months	Moderate
25	Lipson et al. (2019) [9]	Cross-sectional	USA	155,026	General	A convenience samples of universities across the main regions of the USA with random sample of students attending these universities between 2007 and 2017	- Symptoms of depression - Suicidal ideation - Stigma - Mental health service use in the last 12 months	Moderate
26	Lipson et al. (2021) [23]	Cross-sectional	USA	58,063	At risk	A convenience sample of universities and community colleges across the main census regions of the USA with a random sample of students attending these institutions between 2016 and 2019	- Symptoms of depression in the last 2 months - Symptoms of anxiety in the last 2 months - Eating disorder behaviours - Non-suicidal self-injurious behaviours - Suicide ideation - Academic performance - Mental health service use in the last 12 months	Moderate
27	Lu et al. (2014) [74]	Cross-sectional	Australia	144	Subgroup	A convenience sample of students recruited from Chinese student associations in universities across Australia	- Symptoms of psychological distress - Barriers to help-seeking - Willingness to seek help - Help-seeking behaviours in last 12 months	Moderate
28	Nash et al. (2017) [58]	Cross-sectional	USA	7992	General	Convenience sample of major public research state sponsored universities across the USA. All undergraduate students enrolled at these universities were sampled	- Academic status - Socioeconomic status - Psychological and physical conditions - Stereotypical views -Understand of others - Mental health service use in the last 12 months	Moderate
29	Sifat et al. (2022) [28]	Cross-sectional	Bangladesh	350	General	A sample of students from 27 universities in Bangladesh, mostly (62.8%) from Jahangimagar University sampled in 2020	- Perceived need for intervention - Positive feeling towards services - Relatedness - Autonomy - Competency - Current non-clinical mental health practice - Perceived stress - Symptoms of depression - Mental health service use in the last 12 months	Moderate
30	Romano et al. (2022) [72]	Cross-sectional	USA	22,171	At risk	A convenience samples of universities across the main regions of the USA with random sample of students with a positive screen for eating disorder symptoms attending these universities in the 2019–2020 wave	- Eating disorder symptoms - Eating disorder diagnosis - Mental health service use in the last 12 months	High
31	Connor et al. (2022) [71]	Cross-sectional	USA	1892	Subgroup	A convenience sample of universities in Oregon with a sample of sexual and gender minority students	- Mental health symptoms - Helpfulness of service utilization - Barriers to on campus service utilisation - Awareness of services - Mental health service use while at university	Moderate
32	Dunbar et al. (2017) [41]	Cross-sectional	USA	33,220	Subgroup	A sample of all University of California (UC), California State University (CSU), California Community Colleges (CCC) receiving mental health funding and a random sample of CCCs not receiving funding. All enrolled students and staff in these universities were sampled in 2013. Students identifying as lesbian, gay, bisexual, queer or questioning (LGBQQ) were compared with non-LGBQQ students	- Psychological health - Mental health related academic impairment - Awareness of campus mental health services - Campus mental health climate - Mental health service use while at university	Moderate
33	Sontag-Padilla et al. *(2016)* [47]	Cross-sectional	USA	33,943	General	A sample of all UC, CSU, CCCs receiving mental health funding and a random sample of CCCs not receiving funding. All enrolled students and staff in these universities were sampled in 2013	- Coping - Mental health related academic impairment - Awareness of campus mental health services - Campus mental health climate - Mental health service use while at university	Moderate
34	Turner et al. (2015) [59]	Secondary data analysis	USA	730,785	Subgroup	A sample of all students up to age 50 who had interacted with university health centres at a convenience sample of universities across the country between January 2011 and May 2014	- Type of health service visits - Diagnoses	Moderate
35	Xiao et al. (2017) [60]	Secondary data analysis	USA	473,338	General	All universities part of the Center for Collegiate Mental Health Practice Research Network in the USA between 2010 and 2015	- Assessment of treatment history, history of events and symptoms, current problems - Symptoms of mental disorder - Mental health service use of university counselling service	Moderate
36	Albright et al. (2019) [69]	Cross-sectional	USA	2289	Subgroup	A convenience sample of universities across the USA. Random sample of students within those universities. Only students who identified as American Indian, Alaskan Native, or Native Hawaiian with and without army deployment between 2011–2014	- Discrimination - Army deployment - Financial stress - Health insurance coverage - Substance use - Diabetes diagnosis or treatment in the previous 12 months - Suicidal ideation in lifetime - Lifetime mental health service utilization	Low
37	Artime et al. (2019) [62]	Cross-sectional	USA	19,861	At risk	A convenience sample of universities across the USA. Within these universities a random sample of students who had experienced trauma related to interpersonal violence or a history of army deployment in 2015	- Trauma exposure - Symptoms of mental disorder - Lifetime mental health service use	Moderate
38	Baams et al. (2018) [63]	Cross-sectional	USA	25,844	Subgroup	A random sample of four-year universities participating in the National Research Consortium of Counselling Centers in High Education. Students who were lesbian, gay, bisexual, or questioning attending these universities were sampled	- Future resource use - Ability to cope during times of stress - Suicidal ideation in lifetime - Lifetime mental health history - Relationship status - Lifetime mental health service use	Moderate
39	Bonar et al. (2015) [64]	Cross-sectional	USA	1439	At risk	Students who were returning members of the army with experience in combat were sampled using a total design method between October 2011 and July 2013	- Symptoms of depressive - Anxiety symptoms - Symptoms of Post-Traumatic Stress - Alcohol use - Physical and mental health functioning - Perceived stigma and barriers to care - Lifetime mental health service use	Moderate
40	Kerr et al. (2013) [65]	Cross-sectional	USA	6689	Subgroup	A convenience sample of universities across the USA and a random sample of undergraduate female students aged 18–25 years old attending those institutions between 2008–2009	- Symptoms of mental disorder - Lifetime mental health service use	Low
42	Karaffa et al. (2019) [61]	Cross-sectional	USA	573	Subgroup	A convenience sample of American Veterinary Medical Association on Education colleges in the USA. All students enrolled in these colleges were sampled	- Symptoms of depression - Symptoms of anxiety - Alcohol use - Non suicidal self-injurious behaviours - Suicidal thoughts - Lifetime mental health service use	Low
43	Linden et al. (2021) [78]	Cross-sectional	Canada	137,235	General	A convenience sample of 32 universities in 2013, 41 universities in 2016 and 58 universities 2019. Within these universities a random sample of students were chosen	- Symptoms of stress and psychological distress - Mental illness diagnosis - Help-seeking behaviours	Moderate
44	Rice (2015) [66]	Cross-sectional	USA	26,451	At risk	A stratified random sample of university counselling centres that were part of National Research Consortium of Counselling Centers in Higher Education across the main four regions of the USA in 2003. Those reporting use were those who had experienced suicidal thoughts or behaviour	- Suicidal ideation - Severity of suicidal ideation - Primary care service use - Mental health service use in lifetime, previous 12 months and while at university	Moderate

#### iii Study participants

Sample sizes varied substantially ranging from 15 to 730,785 participants. Most studies included general samples of student attending a university with fifteen studies studying specific subgroups of students [41, 44, 51, 52, 58, 59, 61, 63, 65, 69–71, 73–76]. Thirteen studies included samples of students ‘at risk’ [23, 48–50, 56, 57, 62, 64, 66, 68, 72, 79, 80]. Two studies sampled university faculty members, in addition to university students, although these participants were not asked about mental health service use [41, 47]. One study included students at community college and 4-year institutions in the USA [23].

#### iv Mental health services

Overall, most estimates were associated with services classified into the outpatient service category of the DESDE instrument (see Table 2). Seventy-four estimates associated with thirty-seven studies were outpatient services [9, 20, 28, 41, 43–52, 54, 55, 57, 59, 61–67, 70–73, 75–80]. Thirty-seven estimates associated with twenty-two studies could be classed as multiple service categories [9, 20, 23, 41, 47, 50, 53, 56, 57, 61–66, 68–71, 74, 78]. Residential service category was appropriate for seven estimates associated with five studies [9, 57, 61, 66, 70]. Inter-rater agreement for service coding was *Κ* = 0.89, indicating strong agreement [40].

**Table 2:** Service categories and mental health service use

Study #	Author (year)	Mental health service categories							Service location	Treatment	Service as described	Percentage of students who used the service	Reporting period
		Information (I)	Assessment (A)	Self-help and voluntary (S)	Outpatient (O)	Day care (D)	Residential (R)	Multiple service categories					
*Single institution sample*													
1	Bastos et al. (2022) [76]				•				On or off campus	Any	Any	38.5%	Current
					•				On or off campus	High intensity	Psychotherapy and medication	17.1%	
					•				On or off campus	High Intensity	Psychotherapy	15%	
					•				On or off campus	High intensity	Medication	6.8%	
					•				On or off campus	Any	Other treatment	0.3%	
2	Huang et al. (2020) [48]				•				On or off campus	Any	Any	60%	Current
3	Gebreegziabher et al. (2019) [79]				•				On or off campus	High intensity	Doctor	14.9%	Previous 2 weeks
					•				On or off campus	High intensity	Mental health professional	7%	
4	Jennings et al. (2015) [49]				•				On or off campus	High intensity	Primary care provider	14.7%	Previous 2 months
					•				Off campus	Any	Off campus mental health provider	13.7%	
					•				On or off campus	High intensity	Medication	12.6%	
					•				On or off campus	Low intensity	Therapy	17.9%	
					•				On or off campus	Any	Any	68%	
5	Cranford et al. (2008) [43]				•				On or off campus	High intensity	Any	17.9%	Previous 12 months
					•				On or off campus	High intensity	Medication	12.41%	
					•				On or off campus	Low intensity	Counselling	10.56%	
6	Eisenberg et al. (2007) [67]				•				On or off campus	High intensity	Medication	9%	Previous 12 months
					•				On or off campus	Low intensity	Counselling	10%	
					•				On or off campus	Any	Any	15%	
7	Eisenberg et al. (2011) [46]				•				On or off campus	High intensity	Counselling	9.4%	Previous 12 months
					•				On or off campus	Low intensity	Counselling	10.7%	
					•				On or off campus	Any	Any	15.1%	
8	Jardon et al. (2022) [70]							• (O, S)	On campus	Any	Overall use of campus services	39%	Previous 12 months
					•				On campus	High intensity	Mental illness treatment	16%	
								• (O, R, S, I)	Off campus	Any	Overall use of off campus services	66%	
					•				Off campus	High intensity	Therapy	20%	
							•		Off campus	Specialist	Hospital	2%	
					•				Off campus	Any	Other mental health services	16%	
9	Lee et al. (2021) [50]				•				On campus	Any	On campus services	33.5%	Previous 12 months
								• (I, S, O, R)	Off campus	Any	Off campus services	25.7%	
10	Chang et al. (2013) [51]				•				On campus	Any	Any	21%	Ever while at university
11	Williams et al. (2021) [68]				•				On campus	Low intensity	University counselling Services	36.4%	Ever while at university
					•				On campus	Any	University Health Services	70.9%	
		•							On campus	Low intensity	The Wellness Resource Center	32.5%	
12	Nilsson et al. (2004) [52]				•				On campus	Low intensity	Counselling service	2.6%	Ever while at university
13	Ryan et al. (2017) [73]				•				On or off campus	Any	Any	75%	Ever while at university
14	Smith et al. (2021) [53]							• (O, R)	Off campus	Any	Off campus services	40%	Ever while at university
								• (O, I)	On campus	Any	On campus services	68%	
15	Yorgason et al. (2008) [54]				•				On or off campus	Any	Any	17%	Ever while at university
16	Liu et al. (2017) [77]				•				On campus	Low intensity	Counselling services	5.1%	Ever while at university
17	Leao et al. (2011) [75]				•				On campus	Low intensity	On campus counselling services	26%	Ever while at university
				•					On campus	Advice and support	Mentoring service	59%	
18	Giusti et al. (2020) [80]				•				On or off campus	Any	Any	22.3%	Ever
19	Bourdon et al. (2020) [55]				•				On or off campus	Low intensity	Counselling	42.4%	Ever
								• (O, R)	On or off campus	Specialist	Psychiatrist	20.1%	
								• (O, R)	On or off campus	High intensity	Medical professional	20.8%	
					•				On campus	Low intensity	University counselling services	19.9%	
*Multiple institution samples*													
20	Dyrbye et al. (2015) [56]							• (O, R)	On campus	High intensity	Mental health specialist associated with the medical school	68.2%	Previous 12 months
								• (O, R)	Off campus	High intensity	Mental health specialist not associated with the medical school	24.7%	
21	Eisenberg et al. (2011) [45]				•				On or off campus	High intensity	Medication	13.7%	Previous 12 months
					•				On or off campus	Low intensity	Counselling	14.8%	
					•				On or off campus	Any	Any	21.8%	
22	Eisenberg et al. (2012) [20]				•				On or off campus	High intensity	Medication	9%	Previous 12 months
					•				On or off campus	Low intensity	Counselling	16.2%	
								• (O, R)	On or off campus	Any	Any	19.7%	
23	Fischbein et al. (2019) [44]				•				On or off campus	High intensity	Medication	20%	Previous 12 months
					•				On or off campus	Low intensity	Counselling	16.6%	
24	Han et al. (2016) [57]				•				On or off campus	Any	Outpatient	21.4%	Previous 12 months
							•		On or off campus	Specialist	Inpatient	4.5%	
								• (O, R)	On or off campus	Any	Any	37.1%	
25	Lipson et al. (2019) [9]				•				On campus	Low intensity	On campus services	11.8%	Previous 12 months
							•		On or off campus	Specialist	Psychiatric emergency	1%	
								• (O, R)	On or off campus	High intensity	Other locations	8.7%	
								• (O, R)	On or off campus	Any	Any	33.8%	
26	Lipson et al. (2021) [23]				•				On or off campus	Low intensity	Therapy	36.9%	Previous 12 months
					•				On or off campus	High intensity	Medication	30.2%	
					•				On campus	Any	On campus services	20.6%	
27	Lu et al. (2014) [74]							• (I, S, O, R)	On or off campus	Any	Any	49%	Previous 12 months
								• (O, R)	On or off campus	High intensity	Medical doctor	6.3%	
								• (O, R)	On or off campus	Specialist	Psychiatrist	5.6%	
28	Nash et al. (2017) [58]				•				On campus	Low intensity	Counselling	11.1%	Previous 12 months
29	Sifat et al. (2022) [28]				•				On or off campus	Any	Medication or support from a mental health professional	7.1%	Previous 12 months
30	Romano et al. (2022) [72]				•				On or off campus	High intensity	Therapy or counselling	38%	Previous 12 months
31	Conner et al. (2022) [71]				•				On campus	High intensity	Psychological services	23.3%	Ever while at university
					•				On campus	Specialist	Substance use services	2.2%	
								• (O, R, S)	Off campus	Any	Off campus	12.1%	
					•				Off campus	Specialist	Off campus substance use services	1.5%	
32	Dunbar et al. (2017) [41]				•				On campus	Any	On campus services	7.0%	Ever while at university
								• (I, S, O, R)	Off campus	Any	Off campus services	9.5%	
								• (I, S, O, R)	On or off campus	Any	Any	13.7%	
34	Sontag-Padilla et al. *(2016)* [47]				•				On campus	Any	On campus services	10%	Ever while at university
								• (I, S, O, R)	Off campus	Any	Off campus services	10%	
								• (I, S, O, R)	On or off campus	Any	Any	20%	
35	Turner et al. (2015) [59]				•				On campus	Low intensity	Counselling	21%	Ever while at university
36	Xiao et al. (2017) [60]				•				On campus	Low intensity	Counselling	8.95%	Ever while at university
37	Albright et al. (2020) [69]				•				On campus	Low intensity	University Mental Health Service	15%	Ever
38	Artime et al. (2019) [62]				•				On or off campus	Low intensity	Counselling	36.5%	Ever
								• (O, R)	On or off campus	Specialist	Psychiatrist	13%	
								• (O, R)	On or off campus	High intensity	Another medical provider	15.5%	
39	Baams et al. (2018) [63]				•				On or off campus	Low intensity	Counselling	40.7%	Ever
								• (O, R)	On or off campus	Specialist	Psychiatrist	12.9%	
								• (O, R)	On or off campus	High intensity	Another medical provider	10.1%	
40	Bonar et al. (2015) [64]							• (O, R)	On or off campus	Any	Any mental health treatment	46.9%	Ever
								• (O, R)	On or off campus	Any	Veteran Affairs centre treatment	30.9%	
					•				On or off campus	Low intensity	Psychotherapy	33.3%	
					•				On or off campus	High intensity	Medications	22.2%	
								• (O, R)	On or off campus	Any	Military facility	25.9%	
								• (O, R)	On or off campus	Any	Civilian facility	24.7%	
41	Kerr et al. (2013) [65]				•				On or off campus	Low intensity	Counselling	46.7%	Ever
								• (O, R)	On or off campus	Specialist	Psychiatrist	19.9%	
								• (O, R)	On or off campus	High intensity	Another medical provider	19.9%	
					•				On campus	Low intensity	University counselling	22.3%	
42	Karaffa et al. (2019) [61]							• (O, R)	On or off campus	Any	Any	68.6%	Ever
					•				On or off campus	Low intensity	Counselling	62.5%	
					•				On or off campus	High intensity	Medication	36.8%	
					•				On or off campus	Low intensity	Family counselling	14.7%	
					•				On or off campus	Low intensity	Group counselling	8%	
							•		On or off campus	Specialist	Crisis	5.4%	
43	Linden et al. (2021) [78]				•				On or off campus	Low intensity	Therapy	37.5%	Ever
					•				On campus	Low intensity	University counselling	18.5%	
								• (O, R)	On or off campus	Specialist	Psychiatrist	12.6%	
								• (O, R)	On or off campus	High intensity	Another medical provider	20.9%	
								• (O, R)	On or off campus	Any	Any	44.7%	
44	Rice (2015) [66]				•				On or off campus	High intensity	Counselling	36%	Previous 12 months
								• (O, R)	On or off campus	Specialist	Psychiatrist	12%	
					•				On or off campus	High intensity	General medicine provider	10%	
					•				On campus	High intensity	University counselling on a small campus	25.5%	Ever at uni
					•				On campus	High intensity	University counselling on a medium campus	20%	
					•				On campus	High intensity	University counselling on a large campus	19%	
							•		Off campus	Specialist	Hospitalisation among students on a small campus	4%	Ever
							•		Off campus	Specialist	Hospitalisation among students on a medium campus	3%	
							•		Off campus	Specialist	Hospitalisation among students on a large campus	3%	

Across the service categories, 38 estimates related to services providing a range of treatments, 1 providing advice and support, 25 providing low intensity treatment, 35 related to high intensity treatment and 17 related to specialist treatment. Of these estimates thirteen related to services located off campus; 29 were on campus, whereas the remaining 79 estimates could have been located on or off a university campus.

#### v Defining and measuring use of health services

While all studies implicitly conceptualised mental health service use as an event or occurrence by a person in a time-period, the operational assessment was heterogeneous. In the cross-sectional and longitudinal studies, measurement varied by recall period and by item wording [9, 20, 23, 28, 41, 43–45, 47, 49–51, 53–56, 58, 61–75, 78, 79]. Only one study used a validated instrument assessing use over the previous two weeks [79], one asked student about their use over the previous two months [49], sixteen over the last 12 months [9, 23, 28, 42–46, 50, 56–58, 67, 70, 72, 74], four while students were at university [41, 47, 68, 71], and ten asked participants to report about previous use in their lifetime or ever [55, 61–66, 69, 78]. One cross-sectional study asked student participants to both recall use of university counselling centre while at university, and the students use of other mental health service over their lifetime [66]. Nearly all cross-sectional studies gave participants a binary response option—either yes or no. Only one study used an ordered categorical response option where participants were asked to state whether they had used a particular service using a Likert scale ranging from 1–5 (never-often) [50]. Of the two mixed methods studies one reported current use [48], and the other reported on lifetime use [80]. Secondary analyses of electronic medical records examined number of unique visits per student over the study period [52, 59, 60].

### Quality appraisal

Overall, the quality of the studies included in the review were moderate with around a quarter of the total samples rated as either high [43–46, 56, 67, 72, 79], or low quality [49, 52, 54, 61, 65, 69, 76]. The main area of weakness came from questions related to the validity and reliability of the assessment of mental health service use, with only six studies being rated as “yes” in both questions [45, 46, 56, 67, 74, 79]. A further area of significant weakness was found in question eight which related to whether appropriate statistical analyses had been conducted with four studies rated as “yes” [49, 53, 59, 63] (see Table 1 and Additional file 1: Appendix S7). Inter-rater agreement for quality appraisal was *Κ* = 0.88 indicating strong agreement [40].

### What proportion of university students use mental health services when experiencing psychological distress?

#### i. Overall use of any mental health service

##### Narrative summary (n = 10; k = 11)

Ten studies reporting on students’ use of any mental health service use with estimates ranging between 13.7 and 68.6% of the study population reporting use [9, 41, 47, 50, 53, 57, 61, 64, 70, 71, 74, 78]. Estimates ranged from 13.7 to 68.6% of the study population reporting using a service. It was difficult conclude the source of this variation. The highest estimate, at 68.6%, was the only for an on-campus service. Treatment offered by the service did not appear to be associated with variation across estimates. Broader operational service definitions tended to have higher estimates [53, 74]. For example, in one study 49% of Chinese international students reported using “any form of help”, whereas all other estimates within the same study relating to specific services were low.

There was some evidence to suggest more severe current psychological distress was associated with higher previous mental health service use. For example, in studies with at risk samples reported estimates between 25.7 and 49% [50, 57, 74]. Whereas estimates in general populations of students had a lower range between 19.7 and 45% [9, 47, 53, 78]. Variation also appeared to be related to the reporting period, where studies reporting on lifetime mental health service use tended to have higher estimates [61, 78] (see Tables 1 and 2).

##### Meta-analysis (n = 9; k = 9)

**Fig. 2:**
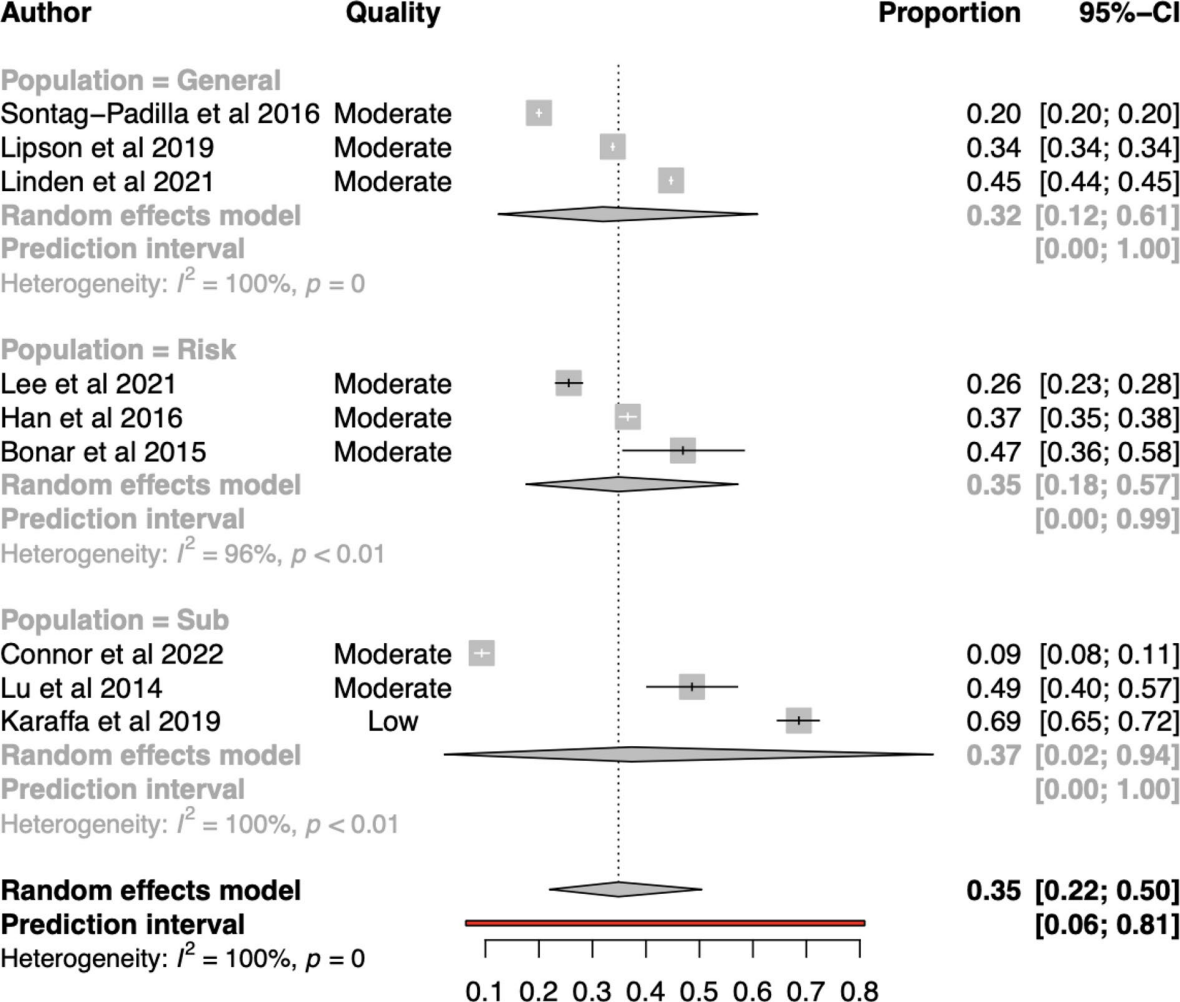
Forest plot for overall mental health service use by population group

The overall pooled proportion effect size using a random effects model was estimated to be 0.35 (95%CI: 0.22;0.50) (see Fig. 2). The between study heterogeneity was estimated at τ^2^ = 0.69, and *Ι* ^2^ = 99.9%. The prediction interval ranged from 0.06 to 0.81. This indicated a wide range of future possible estimates. Overall, these results indicate substantial heterogeneity across the included estimates of mental health service use.

##### Subgroups and meta-regressions for overall use

No variables were associated with an overall reduction in between study heterogeneity using meta-regressions. Subgroup analyses found differences by service location (*Q* = 40.41, df:2, *p* < 0.001), and reporting period (*Q* = 5.92, df:2, *p* = 0.05), However, meta-regressions found lower proportions were associated with off-campus service (*β* = − 1.35, 95%CI:− 2.52; − 0.18, *p* = *0*.03), and higher proportions associated with longer reporting periods (*β* = 0.0043, 95%CI:− 0.001; 0.0075, *p* = 0.02) (see Additional file 1: Appendix S8).

#### ii Overall outpatient use

##### Narrative summary (n = 25; k = 27)

Twenty-five studies reported estimates of students overall outpatient service use with between 2.6 and 75% of the study populations reporting service use [9, 28, 41, 43–52, 54, 57, 59, 61–63, 66, 67, 69–73, 75–77, 80]. Use of on-campus services were lower ranging between 2.6 and 33.5% [9, 41, 47, 50–52, 58–60, 66, 69, 73, 77]. There was only one estimate of off-campus service use at 13.7% [49], whereas the remaining estimates were for services that could be either on or off campus between 7 and 75%. These differences could also be partly explained by differences in population group and treatment offered by the service. The lowest two estimates overall were in subgroups of students namely international students (2.6%) [52], and students in China (5.1%) [77], and among students Bangladeshi universities (7.1%) [28]. Whereas the highest estimates overall and in the category of either on campus or off campus services were in a study of medical students with more severe current psychological distress using services offering potentially any treatment (75%) [73]; previously homeless students or who had been in care where a broad service model had been developed for them (68%) [48], and veterinary students (62.5%) [61]. For this estimate participants reported against the use of “counselling”—which could have a broad interpretation in the USA. A further study also using a broad outpatient service definition was associated with a high estimate of 68% [49]. Overall, studies asking students to recall service use over their lifetime reported a higher range of estimates [61–63, 69, 80], compared to studies with shorter recall periods (see Tables 1 and 2).

##### Meta-analysis for overall outpatient use (n = 24; k = 26)

**Fig. 3:**
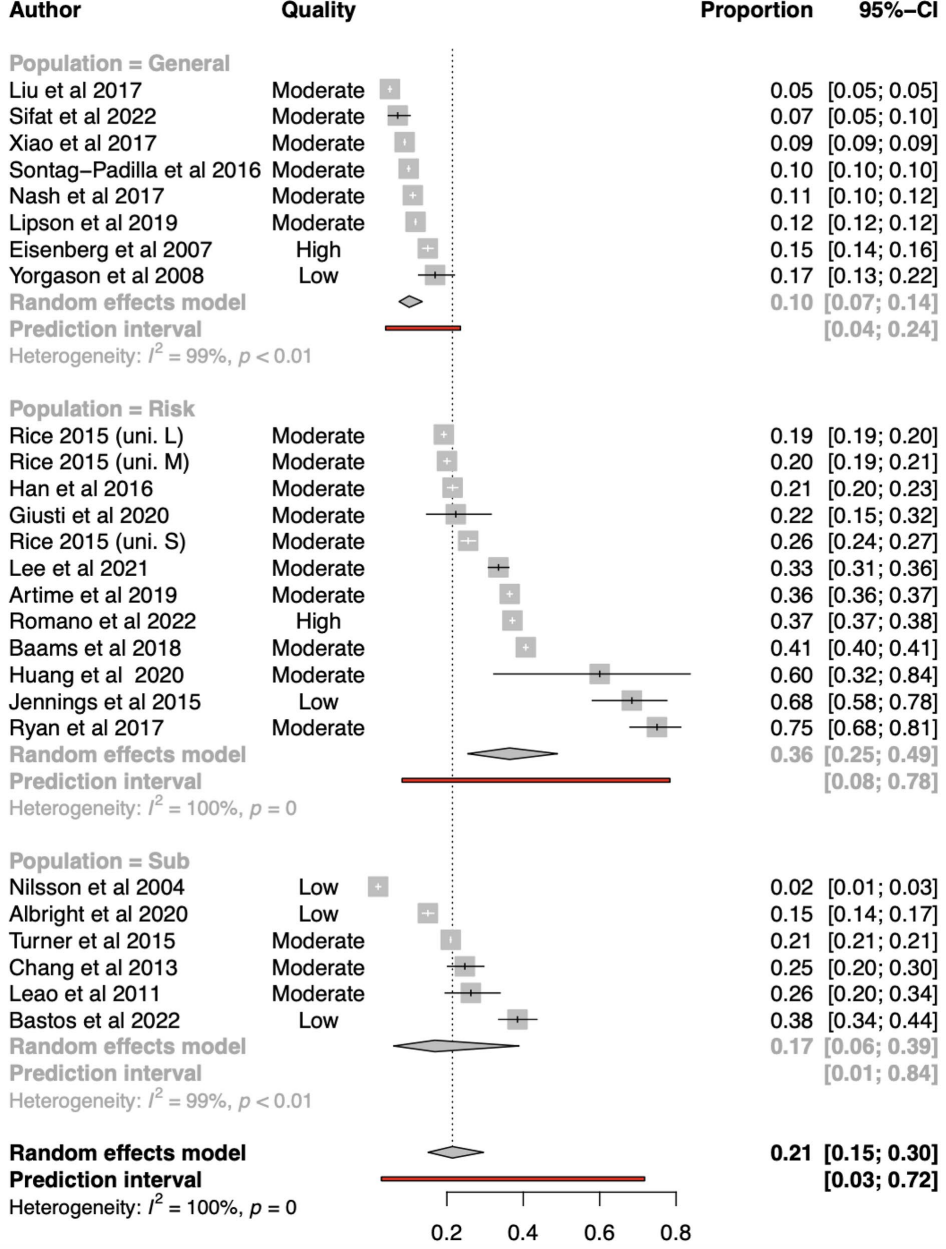
Forest Plot for outpatient overall service use by population group

The overall pooled proportion effect size using a random effects model was estimated to be 0.21 (95%CI = 0.15;0.30) (see Fig. 3). The between study heterogeneity was estimated at τ^2^ = 1.12 and *Ι* ^2^ = 99.9%. The prediction interval ranged from 0.03 to 0.72. This indicated a wide range of future possible estimates. Overall, these results indicate substantial heterogeneity across the included estimates of residential mental health service use.

##### Sub-group analyses and meta-regressions for overall outpatient use

No meta-regression model resulted in a significant reduction in overall between-study heterogeneity. Subgroup analyses found overall differences by service location (*Q* = 9.03, df:1, *p* = 0.002), population group (*Q* = 35.40, df:2, *p* < 0.001), study design (*Q* = 94.68, df:3, *p* < 0.001) (see Additional file 1: Appendix S9). Meta-regressions were conducted finding lower proportions of service utilisation were associated with service providing low intensity treatment (*β* = − 0.91; 95%CI = − 1.78;− 0.04; *p* = 0.04), and on campus services compared than those either on or off campus (*β* = − 1.10, 95%CI: − 1.85; − 0.36, *p* = 0.005). Higher proportions of use were associated in ‘at risk’ to general populations of students (*β* = 1.62, 95%CI:0.88; 2.37, *p* < 0.001), and mixed methods studies (*β* = 2.41, 95%CI:0.08; 4.73, *p* = 0.04).

#### iii Overall residential service use

##### Narrative summary (n = 5; k = 7)

Four studies reported six estimates of residential service use [9, 57, 61, 66, 70], ranging from 1 to 5.4%. Population group appeared to be associated with this variation, with the study reporting on general populations of students having a lower estimate than other groups (see Tables 1 and 2, and Additional file 1: Appendix S10 for a detailed narrative summary).

#### Meta-analysis for overall residential service use (n = 5; k = 7)

**Fig. 4:**
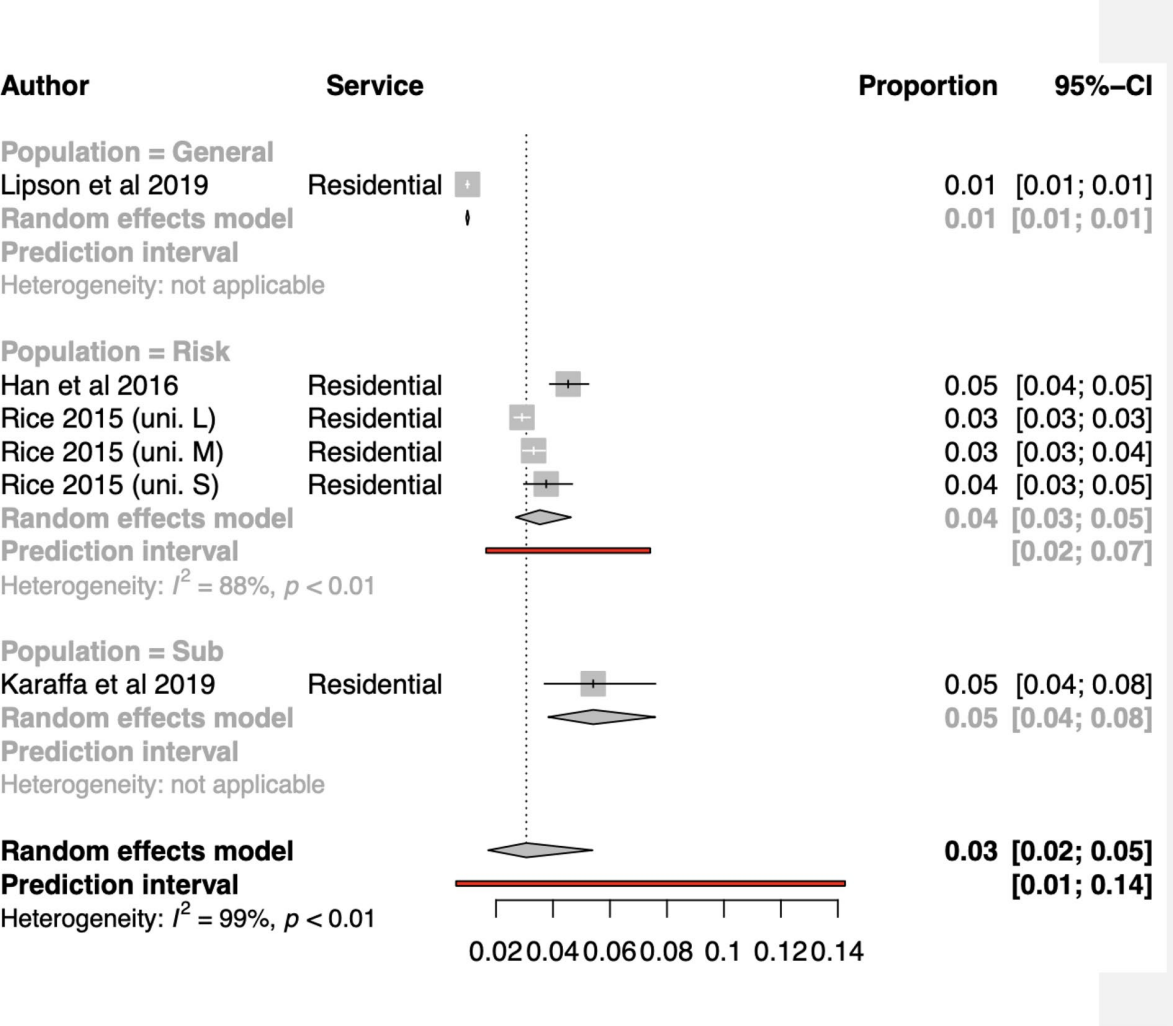
Forest Plot for overall residential service use

The overall pooled proportion effect size using a random effects model was estimated to be 0.03 (95%CI:0.02;0.05) (see Fig. 4). The between study heterogeneity was estimated at τ^2^ = 0.30, and *Ι* ^2^ = 99.4%. There was a prediction interval which ranged from a proportion of 0.007 to 0.12. This indicated a wide range of future possible estimates. Overall, these results indicate substantial heterogeneity across the included estimates of residential mental health service use.

#### Subgroup analyses and meta-regressions for overall residential service use

Meta-regressions only a found a reduction in between study heterogeneity association with population group (τ^2^ = 0.19, *Ι* ^2^ = 86.6%). High estimates were associated with ‘at risk’ students (*β* = 1.29, 95%CI: 0.84; 1.73, *p* = 0.001), and subgroup of students (*β* = 1.50, 95%CI: 0.80; 2.21, *p* = 0.0041) when compared to general populations of students (see Additional file 1: Appendix S10).

### Does service use differ across health service type?

#### i Differences in use by service type

Subgroup analysis conducted using a three-level meta-analysis suggested differences between service types (*F* = 63.25, df:2,39, *p* < 0.001). A meta-regression was conducted where compared to overall service use, both overall outpatient service and overall residential service use was associated with lower proportion of university students reporting using these services (outpatient: *β* = − 0.77, 95%CI: − 1.26; − 0.29; *p* = 0.01; residential: *β* = − 3.05, 95%CI: − 3.63; − 2.47, *p* < 0.001).

Sensitivity analyses found mixed results (see Table 3). For example, excluding estimates of lifetime service use had an attenuating effect on all pooled proportions, whereas removing low quality studies resulted in a lower pooled proportion only in overall service use. When outliers and influential estimates were removed the pooled proportion for overall service use was higher. A reduction in between study heterogeneity was only observed when outliers and influential cases were removed (see Table 3). Sensitivity analyses continued to suggest differences by service location and treatment type for overall outpatient service use, by service location for overall service use, except when excluding estimates of lifetime use (see Additional file 1: Appendix S11, 12 and 13).

**Table 3:** Sensitivity analyses

Excluding estimates of lifetime use						
K	Variable	Proportion	95% CI	*Ι *^*2*^_*level 2*_	*Ι *^*2*^_*level 3*_	*p*subgroup
6	Overall	0.30	0.17; 0.46	98%	0%	< 0.001
22	Outpatient	0.20	0.14; 0.28			
3	Residential	0.02	0.008; 0.052			

Excluding low quality studies						
K	Variable	Proportion	95% CI	*Ι *^*2*^_*level 2*_	*Ι *^*2*^_*level 3*_	*p*subgroup
8	Overall	0.31	0.20; 0.45	98.5%	0%	< 0.001
21	Outpatient	0.22	0.16; 0.30			
6	Residential	0.03	0.02; 0.05			

Excluding influential cases and outliers						
K	Variable	Proportion	95% CI	*Ι *^*2*^_*level 2*_	*Ι *^*2*^_*level 3*_	*p*subgroup
4	Overall	0.38	0.24; 0.54	96.2%	0%	< 0.001
13	Outpatient	0.16	0.11; 0.23			
6	Residential	0.04	0.03; 0.05			

### Further analyses using three-level meta-analysis

#### i Estimates meeting multiple service categories

##### Narrative summary (n = 12; k = 23)

Twelve studies reported on twenty-one estimates associated with services that could be classified as any DESDE classifications [9, 47, 53, 55, 56, 62–65, 70, 74, 78]. These estimates ranged from 5 to 68%. Lower estimates were reported in services offering specialist or high intensity treatment compared to a range of treatments, whereas higher estimates tended be in campus services. In general, studies asking students report service use over their lifetime were associated with higher estimates [55, 62–65, 78] (see Tables 1 and 2).

##### Meta-analysis (n = 12; k = *23)*

The pooled proportion based on the three-level meta-analytic model was 0.20 (95%CI:0.13; 0.31, p < 0.001). *Ι *^2^_*level 3*_ = 82.9% of the total variation can be attributed to between-cluster, and *Ι *^2^_*level 2*_ = 13.76% to within-cluster heterogeneity. We found that the three-level model provided a significantly better fit compared to a two-level model with level 3 heterogeneity constrained to zero (χ^2^_1_ = 8.10, p 0.004).

##### Subgroup analyses and meta-regressions

Subgroup analyses found differences by service location (*F* = 11.201, df:2,18, *p* < 0.001). Meta regressions found on campus, and off campus location was associated with a high proportion when compared service potentially located in both locations (On campus:*β* = 1.83, 95%CI:0.83, 2.83, *p* = 0.001; off campus:*β* = 0.91, 95%CI:0.003, 1.81, *p* = 0.05) (see Additional file 1: Appendix S14, and Appendix S16 for sensitivity analyses).

#### ii Specific outpatient services

##### Narrative summary (n = 13; k = 37)

Between 6.98% and 62.5% of students reporting outpatient service use out of the ten studies and twenty-seven estimates [49, 55, 61, 64–68, 70, 71, 76, 79]. These estimates were between 6.98% and 62.5% of the study populations reporting outpatient service use. It was difficult to determine what this variation was associated with. The definitions used to measure service use may explain some variation. For example, the highest estimate of 62.5% related to individual counselling, and lowest estimate of 6.98% related to group counselling within the same study, and both classed as low intensity treatments [61]. The country a service was located appeared to potentially be associated with some variation. Estimates in a study of students at risk in Ethiopia were both low compared to most other estimates in the USA [79]. In general, higher estimates tended to be in studies asking students to report whether they had ever used a mental health service [49, 55, 61, 64, 65, 68, 78].

##### Meta-analysis (n = 13; k = 37)

The pooled proportion based on the three-level meta-analytic model was 0.19 (95%CI:0.13; 0.28, *p* < 0.001). *Ι *^2^_*level 3*_ = 31.3% of the total variation can be attributed to between-cluster, and *Ι *^2^_*level 2*_ = 64.3% to within-cluster heterogeneity. We did not find that the three-level model provided a significantly better fit compared to a two-level model with level 3 heterogeneity constrained to zero (χ^2^_1_ = 1.99, *p* = 0.16).

##### Subgroup analyses and meta-regressions

Subgroup analyses found differences by treatment type (*F* = 34.83, df:3,33, *p* < 0.001) and service location (*F* = 35.58, df:2,34, *p* < 0.001). Meta regressions found low intensity (*β* = − 0.94, 95%CI: − 1.17, − 0.71, *p* < *0.0*01), specialist treatment (*β* = − 2.06, 95%CI: − 2.81, − 1.32, *p* < *0.0*01) and on campus locations were associated with lower proportions (*β* = − 0.93, 95%CI: − 1.15, − 0.71, *p* < 0.001) (see Additional file 1: Appendix S15, and Appendix S17 for sensitivity analyses).

## Discussion

### Main findings

This is the first systematic review and meta-analysis to synthesize evidence relating to the proportion of university students using mental health services, and how this varies by service type. In summary, we found there are wide variety of services available taking varying proportions of students, although overwhelmingly these were from HICs, in particular the USA. Across studies when estimates were grouped and pooled in service categories, we found around a 1/3 of students use services overall while attending university, with around 1/5 of students using outpatient services, and between 1 and 3% have used services that could be classed as residential. Our findings suggest where there is greater availability of support there is greater use, as indicated by higher use being associated with services offering a range of treatments. There was limited evidence to suggest services on campus were used more than those off campus, and students with more severe current psychological distress were associated with greater past service use. However, there are significant limitations with the current literature, including few international studies, particularly from LMICs, little clarity on how services link together, no studies of patient flow and limited consistent description of services.

### Findings in the context of existing evidence

The finding of the proportion of students using mental health services is broadly consistent with average proportions of students reporting problems in previous literature from the USA and North America. In 2012 around 18% of students reported receiving any form of mental health treatment, and 36% among students with a likely mental health problem [20]. Annual cross-sectional surveys confirm that service use is aligned with prevalence in the USA and Canada with increases in service utilisation between 2007 and 2017 to around one third of university students using services [8, 9]. Comparisons with estimates in non-student populations are difficult to interpret because of heterogeneous measures used to estimate need, limited international longitudinal analyses, and few studies assessing the effect of university on mental health trajectories [4]. A systematic review of service use among non-student young adults found only 16% reported using any mental health service, lower than our findings [81]. This is unlikely to be due to differences in need as individual studies suggest mental disorder has increased in both groups, at a similar rate [10, 11]. US studies featured predominantly in both this previous review and ours, therefore differences in reported service use may reflect differences in the availability of services and insurance coverage between groups in the USA. Studies in non-students included relatively young populations with an average age of 21 [81]. In the USA context, the transition to university could prompt the earlier emergence of mental health difficulties as students may face significant new pressures, a new social context and new financial challenges prompting earlier help seeking [4, 9, 20, 25, 27, 82].

Our review predominantly reports on studies of US university students in four-year institutions, and therefore our findings likely confounded by what is available there. Higher proportions of students using campus services maybe due to student’s awareness of, and ability to reach and pay for these services in comparison to other services [83]. Four-year US institutions receive comparably higher levels of funding than US community colleges, influencing their ability to provide students with comprehensive mental health services [23, 47, 84]. Studies using both national and regional US samples found four-year university students report higher use of services on campus compared to community college students, despite higher prevalence of mental health problems in community colleges [23, 47]. Cost was cited as the most common barrier to seeking help among community college students [23]. International studies included in this review reported different patterns of service use, which may reflect different patterns of service provision, demand among students, and barriers to help seeking [73–75, 78–80]. For example, countries such as Australia where there may be fewer barriers to support outside of university, students sought help from a broad range of providers, most frequent being General Practitioners [73]. The limited number of studies outside the USA may reflect the relatively recent increases in the number and diversity of students attending university in other HIC countries, such as the UK [4]. Only recent research has highlighted the very limited research focus on LMIC [85], perhaps the reflecting the potentially smaller proportion of their national populations attending university compared to most HICs [1]. However, recent efforts through the WHO WMH-ICS indicates some change in this field [6, 16]. This in the context of the growing emphasis on the importance of global mental health and the role higher education might play in contributing to improvements in population health [1, 3].

The level of heterogeneity observed was striking when compared to the published literature potentially illustrating the wide range of services, likely with a range of entry requirements, and populations of students. This could also reflect inequalities in population coverage and use of mental health services relative to need across the student populations, as noted in other literature [18, 21, 22]. A review in non-student populations found being female, Caucasian, homosexual, or bisexual meant you were more likely to use services, which is similar to findings in students [81]. However, in our review, some studies of international students had comparably lower use of services, one study reporting only 2.6% used a service [52]. Other studies examining use in other populations in our review reported much higher proportions, as high as 75% [73]. It may be that variation among students is even greater than non-students due to the wide variety of needs among students. Despite students in the USA and other HICs potentially having more available services, such as those on campus, these may be particularly underutilised by some groups who experience more significant barriers to help-seeking both inside and outside university [18, 21, 22]. If some groups of students are consistently underrepresented in services, it is unlikely activities and interventions these services provide will be appropriate for their needs, and will continue to be underutilised by these students [86].

### Strengths and limitations

This is the first systematic review to summarise and pool evidence quantitatively about the management of student mental health. This allowed us to explore and then quantify variation in the way mental health services are used by university students. However, there are limitations to the current review. Firstly, generalising the findings of this review outside of the USA should be cautioned given the limited number of international studies. Secondly, there were specific challenges to classifying services studies described or listed. For example, it was not always clear whether the services were interpreted in the same way by all participants or services with similar names were comparable to each other between studies. While we double coded a random sample of these services, this could have introduced classification bias when grouping the services in this review. We found some outlying estimates that may have been explained by the broad definitions used. For example, ‘counselling’ could provide help for a range of needs or be interpreted differently by students answering a survey. While other reviews have commented that there is variation by treatment received, service location, and by specific populations of students [20, 31]. There was not always detailed and consistent data across our included studies to thoroughly evaluate these relationships quantitatively. However, we used a range of synthesis methods to understand the literature.

The methods to examine use of mental health services in the included studies were heterogeneous. While most included binary response options, the reporting periods varied. This meant there were challenges determining whether students used a service at university or before they were students and whether students continued to use services from before university or were new presentations. This may have led to an overestimation of the proportion of students using mental health services. However, we did conduct sensitivity analyses where we excluded these estimates and used meta-regressions to control for reporting period in all analyses. Most of the studies were in the USA. We would therefore caution generalising the findings of this review beyond the USA given the specificities of the healthcare system and infrastructure available to students there, in contrast even to other Western countries.

### Implications for practice, policy, and research

The findings from this review emphasise the importance of a range of service provision being available to students who are experiencing psychological distress, and supports current policy efforts to develop well integrated services to help span levels of need. However, reviews in countries with a significant policy emphasis on integration, such as the UK, highlight the challenges defining this process, and the traditionally top-down approach has led to mixed success [87]. The authors argue this may relate to the highly contextual nature of the problems integration aims to address, therefore it should focus on what needs to be done rather than simply the goal of integration [87]. The findings of our review, particularly the variety of services, groups of students and numbers using mental health services, support this point. This emphasises the need for detailed local needs assessments, the co-production of the process of integration with relevant stakeholders, and adaptations to meet the needs of the local student population [32, 87].

Given the important developmental period students often attend university and the potential important role university’s could play in improving population mental health, the findings of the review suggest a series of important avenues for future research. (1) There is a urgent need to conduct robust international studies to understand student mental health need; (2) international research describing service models available to, acceptable to, and used by, students and similar aged young people; (3) given the few students using formal mental health services across all studies identified in this review, international research should continue to understand alternative models and interventions which might be acceptable and accessible students, such as task shifting, the use of technology, and capacity building within social networks [3, 32]; (4) there are no studies of patient flow and how services are linked together which should be a priority of research particularly given the policy emphasis on integration; (5) there is a limited number of studies examining the adequacy of treatment students receive which could help understand how well services are meeting the needs of students who reach services [42]. (6) To understand how best to adapt current care pathways the experiences of students, healthcare professionals and other stakeholders need to be explored. In some HICs qualitative studies have spoken to students, and staff in counselling services [19, 24, 25, 82], however given the variation of services we found in this review our findings emphasize the need to speak to healthcare professionals, students and other young in a range of settings; (7) The observed differences between the findings of this review and a review in non-student populations [81], it is crucial to understand whether university attendance adds additional risk to mental health trajectories. Our findings suggest significant inequalities in access to mental health services among students and settings, the literature should be systematically reviewed to examine this further.

Globally, future research should pay close attention to health and social inequalities between those with and without a university degree. In many countries, particularly those with a small proportions of people ultimately attaining a university degree, there is the potential to exacerbate inequalities by improving the health of a potentially privileged group of people [1, 88]. Any initiatives aiming to address student mental health should be considered in the relation to wider population as part of a broader strategy to improve population mental health [3].

## Conclusion

This review is the first effort to systematically describe mental health services available to students and quantify students’ use of them. Most studies were in HICs, in particularly the USA, where we found around a third of students had used a mental health service, similar to the proportion of students with symptoms indicative of mental disorder. However, we found significant variation in the utilisation of mental health services across populations of students, settings, and countries. There were some services, such as those on-campus, used more than others potentially reflecting supply and demand patterns in the included study settings. The empirical literature to date is very limited in terms of the relatively small number of international studies, and few studies examining how services link together, and how students move between them which limits our understanding of the problems students face. Our findings support the current renewed effort to study student mental health internationally and emphasises the importance of well-integrated services to support students’ needs.

## Supplementary Information

**Additional file 1.** **Appendix S1.** PRISMA checklist.** Appendix S2.** Key words and MeSH terms.** Appendix S3.** Screening tool and eligibility assessment tool.** Appendix S4.** Data Extraction Form (2). ** Appendix S5.** Relevant Sections from the eDESDE-LTC coding framework used for coding services.** Appendix S6.** search results.** Appendix S7.** Quality Appaisal (2).** Appendix S8.** Overall service use.** Appendix S9.** Overall outpatient service use.** Appendix S10.** Overall residential service use.** Appendix S11.** Sensitivity analyses.** Appendix S12.** Sensitivity analyses – overall service use.** Appendix S13.** Sensitivity analyses – overall outpatient service use.** Appendix S14.** Specific service use (multiple DESDE categories) analyses.** Appendix S15.** Specific outpatient service use analyses.** Appendix S16.** Sensitivity analyses - specific service use (multiple DESDE categories).** Appendix S17.** Sensitivity analyses - specific outpatient service use.

## Data Availability

The datasets used and/or analysed during the current study are available from the corresponding author on reasonable request. Other materials are available in Additional file 1: Appendices 1–17.
