# Supplementary material for: University students’ use of mental health services: a systematic review and meta-analysis
Source: Int J Ment Health Syst. 2022 Dec 17;16:57. doi: 10.1186/s13033-022-00569-0 (PMC9758037; doi:10.1186/s13033-022-00569-0)
Supplement: Supplementary file 1 — Additional file 1. Appendix S1. PRISMA checklist. Appendix S2. Key words and MeSH terms. Appendix S3. Screening tool and eligibility assessment tool. Appendix S4. Data Extraction Form (2). Appendix S5. Relevant Sections from the eDESDE-LTC coding framework used for coding services. Appendix S6. search results. Appendix S7. Quality Appaisal (2). Appendix S8. Overall service use. Appendix S9. Overall outpatient service use. Appendix S10. Overall residential service use. Appendix S11. Sensitivity analyses. Appendix S12. Sensitivity analyses – overall service use. Appendix S13. Sensitivity analyses – overall outpatient service use. Appendix S14. Specific service use (multiple DESDE categories) analyses. Appendix S15. Specific outpatient service use analyses. Appendix S16. Sensitivity analyses - specific service use (multiple DESDE categories). Appendix S17. Sensitivity analyses - specific outpatient service use. [file 13033_2022_569_MOESM1_ESM.docx]

**Appendix S1: PRISMA checklist (1)**

| **Section and Topic** | **Item #** | **Checklist item** | **Location where item is reported** |
| --- | --- | --- | --- |
| **TITLE** | | |  |
| Title | 1 | Identify the report as a systematic review. | Page 1 |
| **ABSTRACT** | | |  |
| Abstract | 2 | See the PRISMA 2020 for Abstracts checklist. | Page 1-2 |
| **INTRODUCTION** | | |  |
| Rationale | 3 | Describe the rationale for the review in the context of existing knowledge. | Pages 2-4 |
| Objectives | 4 | Provide an explicit statement of the objective(s) or question(s) the review addresses. | Page 4 |
| **METHODS** | | |  |
| Eligibility criteria | 5 | Specify the inclusion and exclusion criteria for the review and how studies were grouped for the syntheses. | Page 4 |
| Information sources | 6 | Specify all databases, registers, websites, organisations, reference lists and other sources searched or consulted to identify studies. Specify the date when each source was last searched or consulted. | Page 5 |
| Search strategy | 7 | Present the full search strategies for all databases, registers and websites, including any filters and limits used. | Appendix 2 |
| Selection process | 8 | Specify the methods used to decide whether a study met the inclusion criteria of the review, including how many reviewers screened each record and each report retrieved, whether they worked independently, and if applicable, details of automation tools used in the process. | Page 5, 6, Appendix 3 |
| Data collection process | 9 | Specify the methods used to collect data from reports, including how many reviewers collected data from each report, whether they worked independently, any processes for obtaining or confirming data from study investigators, and if applicable, details of automation tools used in the process. | Page 5-7 |
| Data items | 10a | List and define all outcomes for which data were sought. Specify whether all results that were compatible with each outcome domain in each study were sought (e.g. for all measures, time points, analyses), and if not, the methods used to decide which results to collect. | Page 5-7 |
|  | 10b | List and define all other variables for which data were sought (e.g. participant and intervention characteristics, funding sources). Describe any assumptions made about any missing or unclear information. | Page 5-7 |
| Study risk of bias assessment | 11 | Specify the methods used to assess risk of bias in the included studies, including details of the tool(s) used, how many reviewers assessed each study and whether they worked independently, and if applicable, details of automation tools used in the process. | Page 6  Appendix 3, 4. |
| Effect measures | 12 | Specify for each outcome the effect measure(s) (e.g. risk ratio, mean difference) used in the synthesis or presentation of results. | Page 5-7 |
| Synthesis methods | 13a | Describe the processes used to decide which studies were eligible for each synthesis (e.g. tabulating the study intervention characteristics and comparing against the planned groups for each synthesis (item #5)). | Page 5-7 |
|  | 13b | Describe any methods required to prepare the data for presentation or synthesis, such as handling of missing summary statistics, or data conversions. | Page 5-7 |
|  | 13c | Describe any methods used to tabulate or visually display results of individual studies and syntheses. | Page 5-7 |
|  | 13d | Describe any methods used to synthesize results and provide a rationale for the choice(s). If meta-analysis was performed, describe the model(s), method(s) to identify the presence and extent of statistical heterogeneity, and software package(s) used. | Page 5-7 |
|  | 13e | Describe any methods used to explore possible causes of heterogeneity among study results (e.g. subgroup analysis, meta-regression). | Page 6, 7 |
|  | 13f | Describe any sensitivity analyses conducted to assess robustness of the synthesized results. | Page 7 |
| Reporting bias assessment | 14 | Describe any methods used to assess risk of bias due to missing results in a synthesis (arising from reporting biases). | Page 6 |
| Certainty assessment | 15 | Describe any methods used to assess certainty (or confidence) in the body of evidence for an outcome. | N/A |
| **RESULTS** | | |  |
| Study selection | 16a | Describe the results of the search and selection process, from the number of records identified in the search to the number of studies included in the review, ideally using a flow diagram. | Page 7-9 |
|  | 16b | Cite studies that might appear to meet the inclusion criteria, but which were excluded, and explain why they were excluded. | Page 8-9 |
| Study characteristics | 17 | Cite each included study and present its characteristics. | Page 7-28 |
| Risk of bias in studies | 18 | Present assessments of risk of bias for each included study. | Page 7-28 |
| Results of individual studies | 19 | For all outcomes, present, for each study: (a) summary statistics for each group (where appropriate) and (b) an effect estimate and its precision (e.g. confidence/credible interval), ideally using structured tables or plots. | Page 7-28 and Appendix 8-17. |
| Results of syntheses | 20a | For each synthesis, briefly summarise the characteristics and risk of bias among contributing studies. | Page 7-28, Appendix 8-17. |
|  | 20b | Present results of all statistical syntheses conducted. If meta-analysis was done, present for each the summary estimate and its precision (e.g. confidence/credible interval) and measures of statistical heterogeneity. If comparing groups, describe the direction of the effect. | Page 28-36, Appendix 8-17. |
|  | 20c | Present results of all investigations of possible causes of heterogeneity among study results. | Page 28-36, Appendix 8-17. |
|  | 20d | Present results of all sensitivity analyses conducted to assess the robustness of the synthesized results. | Page 28-36, Appendix 11-17. |
| Reporting biases | 21 | Present assessments of risk of bias due to missing results (arising from reporting biases) for each synthesis assessed. | N/A |
| Certainty of evidence | 22 | Present assessments of certainty (or confidence) in the body of evidence for each outcome assessed. | N/A |
| **DISCUSSION** | | |  |
| Discussion | 23a | Provide a general interpretation of the results in the context of other evidence. | Page 37-40 |
|  | 23b | Discuss any limitations of the evidence included in the review. | Page 37-41 |
|  | 23c | Discuss any limitations of the review processes used. | Page 39-41 |
|  | 23d | Discuss implications of the results for practice, policy, and future research. | Page 40-41 |
| **OTHER INFORMATION** | | |  |
| Registration and protocol | 24a | Provide registration information for the review, including register name and registration number, or state that the review was not registered. | Page 4 |
|  | 24b | Indicate where the review protocol can be accessed, or state that a protocol was not prepared. | Page 4 |
|  | 24c | Describe and explain any amendments to information provided at registration or in the protocol. | Page 4 |
| Support | 25 | Describe sources of financial or non-financial support for the review, and the role of the funders or sponsors in the review. | Page 42-43 |
| Competing interests | 26 | Declare any competing interests of review authors. | Page 42-43 |
| Availability of data, code and other materials | 27 | Report which of the following are publicly available and where they can be found: template data collection forms; data extracted from included studies; data used for all analyses; analytic code; any other materials used in the review. | Appendices |

*From:*  Page MJ, McKenzie JE, Bossuyt PM, Boutron I, Hoffmann TC, Mulrow CD, et al. The PRISMA 2020 statement: an updated guideline for reporting systematic reviews. BMJ 2021;372:n71. doi: 10.1136/bmj.n71

For more information, visit: <http://www.prisma-statement.org/>

**Appendix S2: Key words and MeSH terms**

Using a Condition (service use, psychological) Context (mental health service), Population (student) framework (CoCoPop framework).

*Medline*

# Population

| students/ or exp student dropouts/ or exp students, health occupations/ exp students, | | | |
| --- | --- | --- | --- |
| health occupations/ or exp students, dental/ or exp students, medical/ or exp | |  | |
| students, nursing/ or exp students, pharmacy/ or exp students, premedical/ or exp | | |  |
| students, public health/ |  | |  |

1.

1. (Student* adj3 (dental or dentistry or medical or medic* or nurs* or pharmac* or predical or public health or dropout* or drop out*).tw,kf.
2. ((undergraduate* or under-graduate* or postgraduate* or post-graduate* or (research or PhD or doctoral or college or university or higher education) adj3 student*)).tw,kf
3. 1 OR 2 OR 3.

# Context

1. Mental health services/ or exp community mental health services/ or exp emergency services, psychiatric/
2. Counselling/ or directive counselling/ or distance counselling.
3. Psychotherapy/ or exp psychotherapy, person-centered/ or exp psychotherapy, psychoanalytic/ or exp psychotherapy, multiple/ or exp psychotherapy, brief/ or exp psychotherapy, rational-emotive/ or exp psychotherapy, psychodynamic/ or psychotherapy, interpersonal/.
4. Psychotropic drugs/ or antidepressive agents/ or antidepressive agents, secondgeneration/ or antidepressive agents, tricyclic/ or tranquilizing agents/ or anti-anxiety agents/ or antimanic agents/ or antipsychotic agents/
5. Primary health care.
6. ((inpatient* or outpatient* or out patient* or in patient* or emergenc* or ER or urgent or crisis or (accident and emergency) or A&E or specialist or general*) adj2 service*) or emergenc* room* or A&E or (accident and emergency)).tw,kf.
7. ((Mental health or (community adj2 (mental health or psycho* or psychia*)) or psycho* or psychiat* or counsel* or low intensity or high intensity or IAPT or therap*) adj3 (treatment* or service* or provider* or organi$ation* or institution* or system*)).tw,kf.
8. ((anti-depress* or anti-anxiety or anti-psychotic or psychotrop* or anxiolytic or hyponotic* or mood stabli$er*) adj3 (medication* or treatment*)).tw,kf.
9. 5 OR 6 OR 7 OR 8 OR 9 OR 10 OR 11 OR 12.

# Condition

1. Exp health services accessibility/ or exp health equity/ or exp right to health/ or exp universal health care/ or exp health disparities
2. ((Access* or approach, enter* or admit* or availab* or (seek* adj8 (treatment or help or support or healthcare or health care or health-care)) or engage* or Utili$* or (use* adj8 (service or provider or healthcare or health-care or “health care”)) or usage or reach*)).tw,kf.
3. (Equalit* OR inequalit* OR inequit* OR equit* OR disparit*).tw,kf.
4. 14 OR 15 OR 16.

1. Stress, psychological/ or Burnout, psychological/ or exp Psychological Distress/ or psychological trauma/
2. (Psychological distress* OR emotional distress* OR stress disorder*).tw,kf.
3. Exp Mental health/.
4. (Mental* health*).tw,kf.
5. Mental disorders/ or anxiety disorders/ or mood disorders/ or obsessive-compulsive disorder/ or panic disorder/ or phobic disorders/ or phobia, social/ or "bipolar and related disorders"/ or bipolar disorder/ or dissociative disorders/ or depressive disorder, major/ or depressive disorder, treatment-resistant/ or seasonal affective disorder/ or cyclothymic disorder/ or neurotic, disorders/ or personality disorders/ or psychotic disorders/ or psychoses, substance-induced/ or schizophrenia/ or somatoform disorders/ or "trauma and stressor related disorders"/ or adjustment disorders/ or stress disorders, traumatic/.
6. Exp depression/ or anxiety.
7. Mentally ill person/
8. (Delusion* or hallucinat* or schizophren* or “psychosis” or “schizoaffective” or “psychotic” or “paranoid”).tw,kf.
9. (Mood disorder* or mood disturbance* or affective disorder* or affective disturbance* or affective ill* or cyclothymi* or depression or depressive or neurotic or neurosis or adjustment disorder* or anxiety disorder* or anxious or EDNOS or health anxiety or agoraphobia or obsess* or compulsi* or panic or phobi* or ptsd or posttrauma* or post trauma* or somatoform or somati#ation or medical* unexplained or body dysmorphi* or conversion disorder or hypochondria* or trichotillomania or anhedonia* or affective symptoms or mania* or dysthymia* or dysthymic disorder* or disordered personalit* or personality difficult*).tw,kf.
10. ((mental or psychiatri* or psycholog*) adj3 (illness* or health disorder* or disorder* or problem* or health* or well* or difficult* or issue* or symptom*)).tw,kf.
11. feed and eating disorders/ or anorexia nervosa/ or binge-eating disorder/ or bulimia nervosa/ or diabulimia/
12. ((eating disorder* or EDNOS or anorexi* or orthorexi* or bulimi* or diabulimi* or ((binge and (eat* or purg*)) or (compulsive adj2 (eat* or vomit* or purg*)) not (obes* or metabolic syndrome)).tw,kf.
13. self-injurious behavior/ or self mutilation/ or suicide/ or suicidal ideation/ or suicide, attempted/ or suicide, completed/
14. ((self adj (injur* or mutilat*)) or suicide* or suicidal or parasuicid* or para-suicid*).tw,kf.

1. Conduct disorder/ or exp attention deficit disorder with hyperactivity/
2. ((conduct or behave* or antisocial or anti-social or dyssocial or emotional* or internali#ing or externali#ing) adj1 (problem? or difficult* or psychopath*)).tw,kf.
3. ((conduct or behave* or personalit*) adj1 (aggressi* or agressi* or antisocial or antisocial or dyssocial or defian* or delinquen* or disturb* or disrupt* or internali#ing or externali#ing or problem*)).tw,kf.
4. Substance-related disorders/ or alcohol-related disorders/ or illicit Drugs/ or alcoholism/ or binge drinking/

36. ((Alcohol OR substance*) ADJ2 disorder* OR related disorder*).tw,kf.

37. ((((substance adj1 ("use" or abuse)) or binge-drinking or binge drinking or "drug abuse" OR "substance abuse" OR "alcohol abuse" OR "drug dependence" OR "substance dependence" OR "alcohol dependence" OR "drug addiction" OR "substance addiction" OR "alcohol addiction" OR "substance-use disorder" OR "alcohol-use disorder" OR alcoholi* OR binge drink*))).tw,kf.

38. 18 or 19 or 20 or 21 or 22 or 23 or 24 or 25 or 26 or 27 or 28 or 29 or 30 or 31 or 32 or 33 or 34 or 35 or 36 or 37

39. 4 and 13 and 17 and 38

*Embase*

# Population

1. student/ or exp college student/ or exp disabled student/ or exp foreign student/ or exp graduate student/ or exp health student/ or exp non-medical student/ or exp nontraditional student/ or exp phd student/ or exp postgraduate student/ or exp research student/ or exp social work student/ or exp undergraduate student/ or exp university student/ or exp veterinary student/
2. ((Student* adj3 (dental or dentistry or medical or medic* or nurs* or pharmac* or predical or "public health" or dropout* or "drop out")).ti,ab,kw.
3. ((undergraduate* or under-graduate* or postgraduate* or post-graduate* or (research or PhD or doctoral or college or university or "higher education")) adj3 student*).ti,ab,kw.
4. 1 OR 2 OR 3.

# Context

1. Mental health care/ or exp home mental health care/ or mental health service/ or exp mental hospital/ or psychiatric nursing/ or emergency health service/ or exp psychiatric emergency service/ or exp community mental health service/
2. Counseling/ or exp directive counseling/ or exp e-counseling/
3. Psychotherapy/ or exp behavior therapy/ or exp client centered therapy/ or exp cognitive behavioral therapy/ or exp emotion-focused therapy/ or exp interpersonal psychotherapy/ or exp mentalization-based treatment/ or psychodynamic psychotherapy/ or exp rational emotive behavior therapy/ or exp short term psychotherapy/
4. Psychotropic agent/ or exp mood stabilizer/ or psychostimulant agent/ or exp antidepressant agent/ or tranquilizer/ or exp anxiolytic agent/
5. Exp primary health care.
6. (((inpatient* or outpatient* or "out patient" or "in patient" or emergenc* or ER or urgent or crisis or "accident and emergency" or A&E or specialist or general*) adj2 service*) or emergenc* room* or A&E or “accident and emergency”).ti,ab,kw
7. ((“Mental health” or (community adj2 (mental health or psycho* or psychia*)) or psycho* or psychiat* or counsel* or "low intensity" or "high intensity" or IAPT or therap*) adj3 (treatment* or service* or provider* or organi$ation* or institution* or system*)).ti,ab,kw.
8. ((anti-depress* or anti-anxiety or anti-psychotic or psychotrop* or anxiolytic or hyponotic* or mood stabli$er*) adj3 (medication* or treatment*)).ti,ab,kw.
9. 5 OR 6 OR 7 OR 8 OR 9 OR 10 OR 11 OR 12.

# Condition

1. Exp health care access/ or exp health care availability/ or exp health care disparities/ or exp health care distribution/ or exp right to health/ or exp universal health care/ or health equity
2. ((Access* or approach, enter* or admit* or availab* or (seek* adj8 (treatment or help or support or “health care” or healthcare or health-care)) or engage* or Utili$* or (use* adj8 (service or provider or “health care” or healthcare or health-care)) or usage or reach*)).ti,ab,kw.
3. (Equalit* OR inequalit* OR inequit* OR equit* OR disparit*).ti,ab,kw.
4. 14 OR 15 OR 16.
5. Stress/ or exp acute stress/ or exp behavioral stress/ or Burnout/ or exp student burnout/ or exp emotional stress/ or exp interpersonal stress/ or exp life stress/ or exp mental stress/ or social stress/ or exp psychotrauma/
6. (Psychological distress* OR emotional distress* OR stress disorder*).ti,ab,kw.
7. Exp Mental health/.
8. (Mental* health*).ti,ab,kw.
9. Mental disease/ or exp adjustment disorder/ or exp mood disorder/ or exp dissociative disorder/ or exp emotional disorder/ or exp neurosis/ or exp personality disorder/ or exp psychosis/ or exp psychosomatic disorder/ or psychotrauma/ or exp schizophrenia spectrum disorder.
10. Exp depression/ or exp anxiety/
11. Exp mental patient/
12. (Delusion* or hallucinat* or schizophren* or “psychosis” or “schizoaffective” or “psychotic” or “paranoid”).tw,ab,kw.
13. ((((Mood or affective or adjustment or anxiety or conversion or dysthymic) adj2 disorder*) or (mood or affective)) adj2 disturbance*) or affective ill* or cyclothymi* or depression or depressive or neurotic or neurosis or anxious or health anxiety or agoraphobia or obsess* or compulsi* or panic or phobi* or ptsd or posttrauma* or post trauma* or somatoform or somati#ation or medical* unexplained or body dysmorphi* or hypochondria* or trichotillomania or anhedonia* or affective symptoms or mania* or dysthymia* or disordered personalit* or personality difficult*).ti,ab,kw.
14. ((mental or psychiatri* or psycholog*) adj3 (illness* or health disorder* or disorder* or problem* or health* or well* or difficult* or issue* or symptom*)).ti,ab,kw.
15. Eating disorders/ or anorexia nervosa/ or binge-eating disorder/ or bulimia nervosa/ or diabulimia/
16. ((eating disorder* or EDNOS or anorexi* or orthorexi* or bulimi* or diabulimi* or ((binge and (eat* or purg*)) or (compulsive adj2 (eat* or vomit* or purg*)) not (obes* or metabolic syndrome)).ti,ab,kw.
17. Exp automutilation/ or exp suicidal behaviour/
18. ((self adj (injur* or mutilat*)) or suicide* or suicidal or parasuicid* or para-suicid*).ti,ab,kw.
19. Exp conduct disorder/ or behavior disorder/
20. ((conduct or behave* or antisocial or anti-social or dyssocial or emotional* or internali#ing or externali#ing) adj1 (problem? or difficult* or psychopath*)).ti,ab,kw.
21. ((conduct or behave* or personalit*) adj1 (aggressi* or agressi* or antisocial or antisocial or dyssocial or defian* or delinquen* or disturb* or disrupt* or internali#ing or externali#ing or problem*)).ti,ab,kw.
22. Drug dependence/ or exp alcoholism/ or exp drug abuse patterns/ or exp multiple drug abuse/
23. ((Alcohol OR substance*) ADJ2 disorder* OR related disorder*).ti,ab,kw.
24. ((((substance adj1 ("use" or abuse)) or binge-drinking or "binge drinking" or "drug abuse" or "substance abuse" or "alcohol abuse" or "drug dependence" or "substance dependence" or "alcohol dependence" or "drug addiction" or "substance addiction" or "alcohol addiction" or "substance-use disorder" or "alcohol-use disorder" or alcoholi* or binge drink*).ti,ab,kw.
25. 18 or 19 or 20 or 21 or 22 or 23 or 24 or 25 or 26 or 27 or 28 or 29 or 30 or 31 or 32 or 33 or 34 or 35 or 36 or 37
26. 4 and 13 and 17 and 38

*PsycINFO*

# Population

1. Student/ or exp business students/ or exp college student/ or exp international students/ or exp law students/ or exp medical students/ or exp postgraduate students/ or exp reentry students/ or exp transfer students
2. (Student* adj3 (dental or dentistry or medical or medic* or nurs* or pharmac* or predical or public health or dropout* or drop out*)).ti,ab,hw,id.
3. ((undergraduate* or under-graduate* or postgraduate* or post-graduate* or (research or PhD or doctoral or college or university or higher education) adj3 student*)).ti,ab,hw,id.
4. 1 OR 2 OR 3.

# Context

1. Mental health service/ or exp behavioral health services/ or exp community mental health services/ or psychiatric hospital programs/ or exp emergency services/
2. Counseling/ or exp community counseling/ or exp cross cultural counseling/ or exp educational counseling/ or exp grief counseling/ or exp group counseling/ or exp microcounseling/ or exp multicultural counseling/ or psychotherapeutic counseling/
3. Psychotherapy/ or exp adlerian psychotherapy/ or exp affirmative therapy/ or exp brief psychotherapy/ or exp brief relational therapy/ or exp client centered therapy/ or exp emotion focused therapy/ or exp existential therapy/ or exp experiential psychotherapy/ or exp expressive psychotherapy/ or exp gestalt therapy/ or exp group psychotherapy/ or exp humanistic psychotherapy/ or exp individual psychotherapy/ or exp integrative psychotherapy/ or exp interpersonal psychotherapy/ or exp narrative therapy/ or exp network therapy/ or exp psychoanalysis/ or exp psychodynamic psychotherapy/ or exp rational emotive behavior therapy/ or exp solution focused therapy/ or exp strategic therapy/ or exp supportive psychotherapy/
4. Exp antidepressant drugs/ or exp benzodiazepines/ or exp hypnotic drugs/ or exp mood stabilizers/ or exp muscle relaxing drugs/ or exp neurotransmitter uptake inhibitors/ or exp sedatives/ or exp serotonin agonists/ or exp serotonin antagonists/ or exp tranquilizing drugs/
5. Primary health care.
6. ((inpatient* or outpatient* or out patient* or in patient* or emergenc* or ER or urgent or crisis or (accident and emergency) or A&E or specialist or general*) adj2 service*).ti,ab,hw,id.
7. (emergenc* room* or A&E or accident and emergency).ti,ab,hw,id.
8. ((Mental health or (community adj2 (mental health or psycho* or psychia*)) or psycho* or psychiat* or counsel* or low intensity or high intensity or IAPT or therap*) adj3 (treatment* or service* or provider* or organi$ation* or institution* or system*)).ti,ab,hw,id.
9. ((anti-depress* or anti-anxiety or anti-psychotic or psychotrop* or anxiolytic or hyponotic* or mood stabli$er*) adj3 (medication* or treatment*)).ti,ab,hw,id.
10. 5 OR 6 OR 7 OR 8 OR 9 OR 10 OR 11 OR 12 OR 13.

# Condition

1. Exp health services accessibility/ or exp health equity/ or exp right to health/ or exp universal health care/ or exp health disparities
2. ((Access* or approach, enter* or admit* or (seek* adj8 (treatment or help or support or healthcare or health-care or “health care”)) or engage* or Utili$* or (use* adj8 (service or provider or healthcare or health-care or “health care”)) or usage or reach*)).ti,ab,hw,id.
3. (Equalit* OR inequalit* OR inequit* OR equit* OR disparit*).ti,ab,hw,id.
4. 15 OR 16 OR 17.
5. Exp academic stress/ or occupational stress/ or exp psychological stress/ or exp social stress/ or exp distress/
6. (Psychological distress* OR emotional distress* OR stress disorder*).ti,ab,hw,id.
7. Exp Mental health/.
8. (Mental* health*).ti,ab,hw,id.
9. Mental disorders/ or exp affective disorders/ or exp anxiety disorders/ or exp bipolar disorder/ or exp borderline states/ or exp chronic mental illness/ or exp dissociative disorders/ or exp mental disorders due to general medical conditions/ or exp neurosis/ or exp personality disorders/ or exp psychosis/ or exp serious mental illness/ or exp somatoform disorders/ or exp "stress and trauma related disorders"/ or exp thought disturbances/
10. Exp depression/ or anxiety.
11. Exp Psychiatric Patients/
12. (Delusion* or hallucinat* or schizophren* or psychosis or schizoaffective or psychotic or paranoid).ti,ab,hw,id.
13. (Mood disorder* or mood disturbance* or affective disorder* or affective disturbance* or affective ill* or cyclothymi* or depression or depressive or neurotic or neurosis or adjustment disorder* or anxiety disorder* or anxious or EDNOS or health anxiety or agoraphobia or obsess* or compulsi* or panic or phobi* or ptsd or posttrauma* or post trauma* or somatoform or somati#ation or medical* unexplained or body dysmorphi* or conversion disorder or hypochondria* or trichotillomania or anhedonia* or affective symptoms or mania* or dysthymia* or dysthymic disorder* or disordered personalit* or personality difficult*).ti,ab,hw,id.
14. ((mental or psychiatri* or psycholog*) adj3 (illness* or health disorder* or disorder* or problem* or health* or well* or difficult* or issue* or symptom*)).ti,ab,hw,id.
15. Eating disorders/ or exp anorexia nervosa/ or exp binge eating disorder/ or exp bulimia/ or exp "purging (eating disorders)"/ or exp "rumination (eating)"/
16. ((eating disorder* or EDNOS or anorexi* or orthorexi* or bulimi* or diabulimi* or (binge and (eat* or purg*)) or (compulsive adj2 (eat* or vomit* or purg*))) not (obes* or metabolic syndrome)).ti,ab,hw,id.
17. exp self-injurious behavior/ or exp suicide/ or exp suicidal ideation/
18. ((self adj (injur* or mutilat*)) or suicide* or suicidal or parasuicid* or para-suicid*).ti,ab,hw,id.
19. exp conduct disorder/ or behavior disorders/ or exp attention deficit disorder/
20. ((conduct or behave* or antisocial or anti-social or dyssocial or emotional* or internali#ing or externali#ing) adj1 (problem? or difficult* or psychopath*)).tw,kf.
21. ((conduct or behave* or personalit*) adj1 (aggressi* or agressi* or antisocial or antisocial or dyssocial or defian* or delinquen* or disturb* or disrupt* or internali#ing or externali#ing or problem*)).tw,kf.
22. Sub substance use disorder"/ or exp addiction/ or "alcohol use disorder"/ or exp drug abuse/
23. Alcohol abuse
24. ((Alcohol OR substance*) ADJ2 disorder* OR related disorder*).ti,ab,hw,id.
25. ((((substance adj1 ("use" or abuse)) or binge-drinking or binge drinking or "drug abuse" OR "substance abuse" OR "alcohol abuse" OR "drug dependence" OR "substance dependence" OR "alcohol dependence" OR "drug addiction" OR "substance addiction" OR "alcohol addiction" OR "substance-use disorder" OR "alcohol-use disorder" OR alcoholi* OR binge drink*))).ti,ab,hw,id.
26. 19 or 20 or 21 or 22 or 23 or 24 or 25 or 26 or 27 or 28 or 29 or 30 or 31 or 32 or 33 or 34 or 35 or 36 or 37 or 38 or 39.
27. 4 and 14 and 18 and 40

*ERIC*

# Population

1. DE "Students" OR DE "Adult Students" OR DE "Advanced Students" OR DE "African American Students" OR DE "American Indian Students" OR DE "Asian American Students" OR DE "Bilingual Students" OR DE "College Students" OR DE "College Freshmen" OR DE "College Seniors" OR DE "College Transfer Students" OR DE "First Generation College Students" OR DE "Graduate Students" OR DE "In State Students" OR DE "On Campus Students" OR DE "Out of State Students" OR DE "Preservice Teachers" OR DE "Two Year College Students" OR DE "Undergraduate Students" OR DE "Foreign Students" OR DE "Full Time Students" OR DE "Hispanic American Students" OR DE "Low Income Students" OR DE "Minority Group Students" OR DE "Nontraditional Students" OR DE "Nursing Students" OR DE "Part Time Students" OR DE "Pregnant Students" OR DE "Reentry Students" OR DE "Special Needs Students" OR DE "White Students”.
2. TI ( Student* N3 (dental or dentistry or medical or medic* or nurs* or pharmac* or predical or "public health" or dropout* or "drop out*") ) OR AB ( Student* N3 (dental or dentistry or medical or medic* or nurs* or pharmac* or predical or "public health" or dropout* or "drop out*") ) OR KW ( Student* N3 (dental or dentistry or medical or medic* or nurs* or pharmac* or predical or "public health" or dropout* or "drop out*") )
3. TI ( ((undergraduate* or under-graduate* or postgraduate* or post-graduate* or (research or PhD or doctoral or college or university or higher education) N3 student*)) ) OR AB ( ((undergraduate* or under-graduate* or postgraduate* or post-graduate* or (research or PhD or doctoral or college or university or higher education) N3 student*)) ) OR KW ( ((undergraduate* or under-graduate* or postgraduate* or post-graduate* or (research or PhD or doctoral or college or university or higher education) N3 student*)) )
4. 1 OR 2 OR 3.

# Context

1. DE "Crisis Intervention" OR DE "Psychiatric Services" OR DE “Psychological Services” OR DE “Psychiatric Hospitals”.
2. DE "Counseling" OR DE "Group Counseling" OR DE "Individual Counseling" OR DE "Nondirective Counseling" OR DE "Counseling Services".
3. DE “Psychotherapy”
4. DE “Drug Therapy”
5. DE “Primary health care”
6. TI ( ((inpatient* or outpatient* or "out patient*" or "in patient*" or emergenc* or ER or urgent or crisis or "accident and emergency" or A&E or specialist or general*) N2 service*) or "emergenc* room*" or A&E or "accident and emergency") ) OR AB ( ((inpatient* or outpatient* or "out patient*" or "in patient*" or emergenc* or ER or urgent or crisis or ("accident and emergency") or A&E or specialist or general*) N2 service*) or "emergenc* room*" or A&E or "accident and emergency") ) OR KW ( ((inpatient* or outpatient* or "out patient*" or "in patient*" or emergenc* or ER or urgent or crisis or "accident and emergency" or A&E or specialist or general*) N2 service*) or "emergenc* room*" or A&E or "accident and emergency") )
7. TI ( ((Mental health or (community N2 (mental health or psycho* or psychia*)) or psycho* or psychiat* or counsel* or low intensity or high intensity or IAPT or therap*) N3 (treatment* or service* or provider* or organi?ation* or institution* or system*)) ) OR AB ( ((Mental health or (community N2 (mental health or psycho* or psychia*)) or psycho* or psychiat* or counsel* or low intensity or high intensity or IAPT or therap*) N3 (treatment* or service* or provider* or organi?ation* or institution* or system*)) ) OR KW ( ((Mental health or (community N2 (mental health or psycho* or psychia*)) or psycho* or psychiat* or counsel* or low intensity or high intensity or IAPT or therap*) N3 (treatment* or service* or provider* or organi?ation* or institution* or system*)) )
8. TI ( ((anti-depress* or anti-anxiety or anti-psychotic or psychotrop* or anxiolytic or hyponotic* or mood stabli$er*) N3 (medication* or treatment*)) ) OR AB ( ((anti-depress* or anti-anxiety or anti-psychotic or psychotrop* or anxiolytic or hyponotic* or mood stabli$er*) N3 (medication* or treatment*)) ) OR KW ( ((anti-depress* or anti-anxiety or anti-psychotic or psychotrop* or anxiolytic or hyponotic* or mood stabli$er*) N3 (medication* or treatment*)) )
9. 5 OR 6 OR 7 OR 8 OR 9 OR 10 OR 11 OR 12.

# Condition

1. DE “Access to Health Care”.
2. ((TI ( ((Access* or approach, enter* or admit* or availab* or (seek* N8 (treatment or help or support or healthcare or "health care" or health-care)) or engage* or Utili?* or (use* N8 (service or provider)) or usage or reach*)) ) OR AB ( ((Access* or approach, enter* or admit* or availab* or (seek* N8 (treatment or help or support or healthcare or "health care" or health-care)) or engage* or Utili?* or (use* N8 (service or provider)) or usage or reach*)) ) OR KW ( ((Access* or approach, enter* or admit* or availab* or (seek* N8 (treatment or help or support or healthcare or "health care" or health-care)) or engage* or Utili?* or (use* N8 (service or provider)) or usage or reach*)) )
3. TI ( (Equalit* OR inequalit* OR inequit* OR equit* OR disparit*) ) OR AB ( (Equalit* OR inequalit* OR inequit* OR equit* OR disparit*) ) OR KW ( (Equalit* OR inequalit* OR inequit* OR equit* OR disparit*) )
4. 14 OR 15 OR 16.
5. DE "Burnout" OR DE "Stress Variables".
6. TI ( ("Psychological distress*" OR "emotional distress*" OR "stress disorder*") ) OR AB ( ("Psychological distress*" OR "emotional distress*" OR "stress disorder*") ) OR KW ( ("Psychological distress*" OR "emotional distress*" OR "stress disorder*") )
7. DE “Mental Health”
8. TI ("Mental* Health*") OR AB ("Mental* Health*") OR KW ("Mental* Health*")
9. DE "Mental Disorders" OR DE "Anxiety Disorders" OR DE "Posttraumatic Stress Disorder" OR DE "Emotional Disturbances" OR DE "Psychosomatic Disorders" OR DE "Psychosis" OR DE "Schizophrenia"
10. DE "Depression (Psychology)" OR DE "Anxiety"
11. TI ( (Delusion* or hallucinat* or schizophren* or “psychosis” or “schizoaffective” or “psychotic” or “paranoid”) ) OR AB ( (Delusion* or hallucinat* or schizophren* or “psychosis” or “schizoaffective” or “psychotic” or “paranoid”) ) OR KW ( (Delusion* or hallucinat* or schizophren* or “psychosis” or “schizoaffective” or “psychotic” or “paranoid”) )
12. TI ( ("Mood disorder*" or "mood disturbance*" or "affective disorder*" or "affective disturbance*" or "affective ill*" or cyclothymi* or depression or depressive or neurotic or neurosis or "adjustment disorder*" or "anxiety disorder*" or anxious or EDNOS or health anxiety or agoraphobia or obsess* or compulsi* or panic or phobi* or ptsd or posttrauma* or post trauma* or somatoform or somati#ation or medical* unexplained or body dysmorphi* or conversion disorder or hypochondria* or trichotillomania or anhedonia* or "affective symptoms" or mania* or dysthymia* or dysthymic disorder* or disordered personalit* or personality difficult*) ) OR AB ( ("Mood disorder*" or "mood disturbance*" or "affective disorder*" or "affective disturbance*" or "affective ill*" or cyclothymi* or depression or depressive or neurotic or neurosis or "adjustment disorder*" or "anxiety disorder*" or anxious or EDNOS or health anxiety or agoraphobia or obsess* or compulsi* or panic or phobi* or ptsd or posttrauma* or post trauma* or somatoform or somati#ation or medical* unexplained or body dysmorphi* or conversion disorder or hypochondria* or trichotillomania or anhedonia* or "affective symptoms" or mania* or dysthymia* or dysthymic disorder* or disordered personalit* or personality difficult*) ) OR KW ( ("Mood disorder*" or "mood disturbance*" or "affective disorder*" or "affective disturbance*" or "affective ill*" or cyclothymi* or depression or depressive or neurotic or neurosis or "adjustment disorder*" or "anxiety disorder*" or anxious or EDNOS or health anxiety or agoraphobia or obsess* or compulsi* or panic or phobi* or ptsd or posttrauma* or post trauma* or somatoform or somati#ation or medical* unexplained or body dysmorphi* or conversion disorder or hypochondria* or trichotillomania or anhedonia* or "affective symptoms" or mania* or dysthymia* or dysthymic disorder* or disordered personalit* or personality difficult*) )
13. TI ( ((mental or psychiatri* or psycholog*) N3 (illness* or health disorder* or disorder* or problem* or health* or well* or difficult* or issue* or symptom*)) ) OR AB ( ((mental or psychiatri* or psycholog*) N3 (illness* or health disorder* or disorder* or problem* or health* or well* or difficult* or issue* or symptom*)) ) OR KW ( ((mental or psychiatri* or psycholog*) N3 (illness* or health disorder* or disorder* or problem* or health* or well* or difficult* or issue* or symptom*)) )
14. DE “Eating Disorders”
15. TI ( (("eating disorder*" or EDNOS or anorexi* or orthorexi* or bulimi* or diabulimi* or ((binge and (eat* or purg*)) or (compulsive N2 (eat* or vomit* or purg*)) not (obes* or "metabolic syndrome")) ) OR AB ( (("eating disorder*" or EDNOS or anorexi* or orthorexi* or bulimi* or diabulimi* or ((binge and (eat* or purg*)) or (compulsive N2 (eat* or vomit* or purg*)) not (obes* or "metabolic syndrome")) ) OR KW ( (("eating disorder*" or EDNOS or anorexi* or orthorexi* or bulimi* or diabulimi* or ((binge and (eat* or purg*)) or (compulsive N2 (eat* or vomit* or purg*)) not (obes* or "metabolic syndrome")) )
16. DE “Self Destructive Behavior” OR DE “Suicide”
17. TI ( ((self N2 (injur* or mutilat*)) or suicide* or suicidal or parasuicid* or para-suicid*) ) OR KW ( ((self N2 (injur* or mutilat*)) or suicide* or suicidal or parasuicid* or para-suicid*) ) OR AB ( ((self N2 (injur* or mutilat*)) or suicide* or suicidal or parasuicid* or para-suicid*) )
18. DE “Behavior Problems”
19. TI ( ((conduct or behave* or antisocial or anti-social or dyssocial or emotional* or internali#ing or externali#ing) N1 (problem? or difficult* or psychopath*)) ) OR AB ( ((conduct or behave* or antisocial or anti-social or dyssocial or emotional* or internali#ing or externali#ing) N1 (problem? or difficult* or psychopath*)) ) OR KW ( ((conduct or behave* or antisocial or anti-social or dyssocial or emotional* or internali#ing or externali#ing) N1 (problem? or difficult* or psychopath*)) )
20. TI ( ((conduct or behave* or personalit*) N1 (aggressi* or agressi* or antisocial or antisocial or dyssocial or defian* or delinquen* or disturb* or disrupt* or internali#ing or externali#ing or problem*)) ) OR AB ( ((conduct or behave* or personalit*) N1 (aggressi* or agressi* or antisocial or antisocial or dyssocial or defian* or delinquen* or disturb* or disrupt* or internali#ing or externali#ing or problem*)) ) OR KW ( ((conduct or behave* or personalit*) N1 (aggressi* or agressi* or antisocial or antisocial or dyssocial or defian* or delinquen* or disturb* or disrupt* or internali#ing or externali#ing or problem*)) )
21. Substance-related disorders/ or alcohol-related disorders/ or illicit Drugs/ or alcoholism/ or binge drinking/
22. TI ( ((((substance N1 ("use" or abuse)) or binge-drinking or binge drinking or "drug abuse" OR "substance abuse" OR "alcohol abuse" OR "drug dependence" OR "substance dependence" OR "alcohol dependence" OR "drug addiction" OR "substance addiction" OR "alcohol addiction" OR "substance-use disorder" OR "alcohol-use disorder" OR alcoholi* OR binge drink*))) ) OR AB ( ((((substance N1 ("use" or abuse)) or binge-drinking or binge drinking or "drug abuse" OR "substance abuse" OR "alcohol abuse" OR "drug dependence" OR "substance dependence" OR "alcohol dependence" OR "drug addiction" OR "substance addiction" OR "alcohol addiction" OR "substance-use disorder" OR "alcohol-use disorder" OR alcoholi* OR binge drink*))) ) OR KW ( ((((substance N1 ("use" or abuse)) or binge-drinking or binge drinking or "drug abuse" OR "substance abuse" OR "alcohol abuse" OR "drug dependence" OR "substance dependence" OR "alcohol dependence" OR "drug addiction" OR "substance addiction" OR "alcohol addiction" OR "substance-use disorder" OR "alcohol-use disorder" OR alcoholi* OR binge drink*))) )
23. 18 or 19 or 20 or 21 or 22 or 23 or 24 or 25 or 26 or 27 or 28 or 29 or 30 or 31 or 32 or 33 or 34 or 35 or 36
24. 4 and 13 and 17 and 37

# CINAHL Plus

# Population

1. (MH "Students") OR (MH "Student Dropouts") OR (MH "Students, College") OR (MH "Students, Disabled") OR (MH "Students, Foreign") OR (MH "Students, Health Occupations+") OR (MH "Students, Minority") OR (MH "Students, Non-Traditional") OR (MH “Students, Pre-Nursing")
2. TI ( (Student* N3 (dental or dentistry or medical or medic* or nurs* or pharmac* or predical or "public health" or dropout* or "drop out*") ) AND AB ( (Student* N3 (dental or dentistry or medical or medic* or nurs* or pharmac* or predical or "public health" or dropout* or "drop out*") )
3. AB ( ((undergraduate* or under-graduate* or postgraduate* or post-graduate* or (research or PhD or doctoral or college or university or higher education) N3 student*)) ) OR TI ( ((undergraduate* or under-graduate* or postgraduate* or post-graduate* or (research or PhD or doctoral or college or university or higher education) N3 student*)) )
4. 1 OR 2 OR 3.

# Context

1. (MH "Substance Abuse and Mental Health Services Administration") OR (MH "Community Mental Health Services") OR (MH "Emergency Services, Psychiatric") OR (MH "Mental Health Services")
2. (MH "Counseling")
3. (MH "Psychotherapy") OR (MH "Crisis Intervention") OR (MH "Interpersonal Psychotherapy") OR (MH "Psychotherapy, Brief") OR (MH "Psychotherapy, Psychodynamic")
4. (MH "Antianxiety Agents+") OR (MH "Antidepressive Agents+") OR (MH "Antimanic Agents+") OR (MH "Antipsychotic Agents+") OR (MH "Psychotropic Drugs") OR (MH "Tranquilizing Agents") OR (MH "Serotonin Uptake Inhibitors+")
5. (MH "Primary Health Care")
6. TI ( ((inpatient* or outpatient* or "out patient*" or "in patient*" or emergenc* or ER or urgent or crisis or "accident and emergency" or A&E or specialist or general*) N2 service*) or "emergenc* room*" or A&E or "accident and emergency") ) OR AB ( ((inpatient* or outpatient* or "out patient*" or "in patient*" or emergenc* or ER or urgent or crisis or ("accident and emergency") or A&E or specialist or general*) N2 service*) or "emergenc* room*" or A&E or "accident and emergency") )
7. TI ( (("Mental health" or (community N2 ("mental health" or psycho* or psychia*)) or psycho* or psychiat* or counsel* or "low intensity" or "high intensity" or IAPT or therap*) N3 (treatment* or service* or provider* or organi?ation* or institution* or system*)) ) OR AB ( (("Mental health" or (community N2 ("mental health" or psycho* or psychia*)) or psycho* or psychiat* or counsel* or "low intensity" or "high intensity" or IAPT or therap*) N3 (treatment* or service* or provider* or organi?ation* or institution* or system*)) )
8. TI ( ((anti-depress* or anti-anxiety or anti-psychotic or psychotrop* or anxiolytic or hyponotic* or mood stabli$er*) N3 (medication* or treatment*)) ) OR AB ( ((anti-depress* or anti-anxiety or anti-psychotic or psychotrop* or anxiolytic or hyponotic* or mood stabli$er*) N3 (medication* or treatment*)) )
9. 5 OR 6 OR 7 OR 8 OR 9 OR 10 OR 11 OR 12.

# Condition

1. (MH "Healthcare Disparities") OR (MH "Health Services Accessibility+") OR (MH "Health Resource Utilization")
2. TI ( ((Access* or approach, enter* or admit* or (seek* N8 (treatment or help or support or healthcare or "health-care")) or engage* or Utili$* or (use* N8 (service or provider or healthcare or health-care or "health care")) or usage or reach*)) ) OR AB ( ((Access* or approach, enter* or admit* or (seek* N8 (treatment or help or support or healthcare or "health-care")) or engage* or Utili$* or (use* N8 (service or provider or healthcare or health-care or "health care")) or usage or reach*)) )
3. TI ( (Equalit* OR inequalit* OR inequit* OR equit* OR disparit*) ) OR AB ( (Equalit* OR inequalit* OR inequit* OR equit* OR disparit*) )
4. 14 OR 15 OR 16.
5. (MH "Stress, Psychological+") OR (MH "Psychological Distress") OR (MH "Psychological Trauma+")
6. TI ( ("Psychological distress*" OR "emotional distress*" OR "stress disorder*") ) OR AB ( ("Psychological distress*" OR "emotional distress*" OR "stress disorder*") )
7. (MH “Mental Health”)
8. (MH "Psychiatric Emergencies") OR (MH "Affective Disorders, Psychotic+") OR (MH "Psychotic Disorders") OR (MH "Personality Disorders+") OR (MH "Adjustment Disorders+") OR (MH "Mental Disorders") OR (MH "Mental Disorders, Chronic") OR (MH "Mental Disorders Diagnosed in Childhood") OR (MH "Neurotic Disorders+").
9. (MH "Depression") OR (MH "Anxiety+")
10. TI ( (Delusion* or hallucinat* or schizophren* or “psychosis” or “schizoaffective” or “psychotic” or “paranoid”) ) OR AB ( (Delusion* or hallucinat* or schizophren* or “psychosis” or “schizoaffective” or “psychotic” or “paranoid”) )
11. TI ( ("Mood disorder*" or "mood disturbance*" or "affective disorder*" or "affective disturbance*" or "affective ill*" or cyclothymi* or depression or depressive or neurotic or neurosis or "adjustment disorder*" or "anxiety disorder*" or anxious or EDNOS or health anxiety or agoraphobia or obsess* or compulsi* or panic or phobi* or ptsd or posttrauma* or post trauma* or somatoform or somati#ation or medical* unexplained or body dysmorphi* or conversion disorder or hypochondria* or trichotillomania or anhedonia* or "affective symptoms" or mania* or dysthymia* or dysthymic disorder* or disordered personalit* or personality difficult*) ) OR AB ( ("Mood disorder*" or "mood disturbance*" or "affective disorder*" or "affective disturbance*" or "affective ill*" or cyclothymi* or depression or depressive or neurotic or neurosis or "adjustment disorder*" or "anxiety disorder*" or anxious or EDNOS or health anxiety or agoraphobia or obsess* or compulsi* or panic or phobi* or ptsd or posttrauma* or post trauma* or somatoform or somati#ation or medical* unexplained or body dysmorphi* or conversion disorder or hypochondria* or trichotillomania or anhedonia* or "affective symptoms" or mania* or dysthymia* or dysthymic disorder* or disordered personalit* or personality difficult*) )
12. TI ( ((mental or psychiatri* or psycholog*) N3 (illness* or health disorder* or disorder* or problem* or health* or well* or difficult* or issue* or symptom*)) ) OR AB ( ((mental or psychiatri* or psycholog*) N3 (illness* or health disorder* or disorder* or problem* or health* or well* or difficult* or issue* or symptom*)) )
13. (MH "Eating Disorders") OR (MH "Anorexia") OR (MH "Anorexia Nervosa") OR (MH "Binge Eating Disorder") OR (MH "Avoidant Restrictive Food Intake Disorder") OR (MH "Bulimia") OR (MH "Bulimia Nervosa")
14. TI ( (("eating disorder*" or EDNOS or anorexi* or orthorexi* or bulimi* or diabulimi* or ((binge and (eat* or purg*)) or (compulsive N2 (eat* or vomit* or purg*)) not (obes* or "metabolic syndrome")) ) OR AB ( (("eating disorder*" or EDNOS or anorexi* or orthorexi* or bulimi* or diabulimi* or ((binge and (eat* or purg*)) or (compulsive N2 (eat* or vomit* or purg*)) not (obes* or "metabolic syndrome")) )
15. (MH "Suicide") OR (MH "Suicidal Ideation") OR (MH "Suicide, Attempted")
16. (MH "Injuries, Self-Inflicted") OR (MH "Self-Injurious Behavior")
17. TI ( ((self N1 (injur* or mutilat*)) or suicide* or suicidal or parasuicid* or para-suicid*) ) AND AB ( ((self N1 (injur* or mutilat*)) or suicide* or suicidal or parasuicid* or para-suicid*) )
18. (MH "Social Behavior Disorders")
19. TI ( ((conduct or behave* or antisocial or anti-social or dyssocial or emotional* or internali#ing or externali#ing) N1 (problem? or difficult* or psychopath*)) ) OR AB ( ((conduct or behave* or antisocial or anti-social or dyssocial or emotional* or internali#ing or externali#ing) N1 (problem? or difficult* or psychopath*)) )
20. TI ( ((conduct or behave* or personalit*) N1 (aggressi* or agressi* or antisocial or antisocial or dyssocial or defian* or delinquen* or disturb* or disrupt* or internali#ing or externali#ing or problem*)) ) OR AB ( ((conduct or behave* or personalit*) N1 (aggressi* or agressi* or antisocial or antisocial or dyssocial or defian* or delinquen* or disturb* or disrupt* or internali#ing or externali#ing or problem*)) )
21. (MH "Alcohol-Related Disorders") OR (MH "Alcohol Abuse+") OR (MH "Alcoholic Intoxication") OR (MH "Alcoholism") OR (MH "Substance Abuse") OR (MH "Substance Use Disorders") OR (MH "Substance Dependence")
22. TI ( ((Alcohol OR substance*) N2 disorder* OR "related disorder*") ) OR AB ( ((Alcohol OR substance*) N2 disorder* OR "related disorder*") )
23. TI ( ((((substance N1 ("use" or abuse)) or binge-drinking or binge drinking or "drug abuse" OR "substance abuse" OR "alcohol abuse" OR "drug dependence" OR "substance dependence" OR "alcohol dependence" OR "drug addiction" OR "substance addiction" OR "alcohol addiction" OR "substance-use disorder" OR "alcohol-use disorder" OR alcoholi* OR binge drink*))) ) OR AB ( ((((substance N1 ("use" or abuse)) or binge-drinking or binge drinking or "drug abuse" OR "substance abuse" OR "alcohol abuse" OR "drug dependence" OR "substance dependence" OR "alcohol dependence" OR "drug addiction" OR "substance addiction" OR "alcohol addiction" OR "substance-use disorder" OR "alcohol-use disorder" OR alcoholi* OR binge drink*))) )
24. 18 or 19 or 20 or 21 or 22 or 23 or 24 or 25 or 26 or 27 or 28 or 29 or 30 or 31 or 32 or 33 or 34 or 35 or 36 or 37
25. 4 and 13 and 17 and 38

**All databases**

1. AND ("Meta-analys*" or "systematic review*" or "meta-synthes*" or "literature review"). Title and keywords.

**Appendix S3: Screening tool and eligibility assessment tool**

**Review purpose**

**Three stages of screening**

1. Screening of a random sample of 10% of the **titles** and **abstracts** from the full set of search results by reviewer 1 (TO) and reviewer 2 (SL).
2. Screening of the remaining titles and abstracts by reviewer 1 (TO).
3. Screening of full text articles against the exclusion criteria by reviewer 1 (TO).

**Process for screening in Rayyan**

- Open Rayyan and login (<https://rayyan.qcri.org/welcome>)
- Open My Reviews and open “*Systematic review and meta-analysis of the prevalence of use of mental health services in university students, the associated student characteristics and settings***”.**
- Click on “*Show*”.
- Go to “*Inclusion decisions*” on the left-hand side of the screen.
- Select or click on article and start screening.
- Include or exclude articles – for stage 1 please provide a reason (for example – “not relevant” would imply the article does not refer to use of mental health services by students).
- If you cannot decide if an article should be included or excluded based on the title and abstract, use the “*Maybe*” button.

**Inclusion**

We are looking for articles that are linked to or address our review question:

What is the prevalence of mental health service use in university students experiencing psychological distress and does this differ by student characteristics and health service type?

To simplify:

- **Context:** Mental health services, systems, organisations, providers (*by mental health service we mean: 'the means by which effective interventions are delivered for the dominant or subdominant intention to improve wellbeing or mental health; examples include outpatient services, day treatment, inpatient wards, community mental health teams, services within general practices, mental health hospitals and university counselling services*).
- **Condition:** Utilisation (*maybe referred to as access, availability, entry, admit, seek help or healthcare or support, engagement or reach ONLY attitudinal variables are only relevant if the indicator confirms previous or current use*) for psychological distress (*maybe distress, mental health disorders, affective symptoms or serious mental illness*).
- **Population:** University, college or higher education students.

The specific inclusion criteria for the review are:

1. Empirical study designs that measure the use or utilisation of a mental health service over a defined time period either as a primary or secondary outcome. For example, ‘cohort studies’, ‘case-control studies’, ‘cross-sectional studies’, ‘observational studies’, ‘longitudinal studies’, ‘time-series studies’, ‘pre-post studies’ or ‘before and after studies.
2. Adults (aged 18+) studying at a university.

**Exclusion**

We would exclude articles where any of the following criteria applies:

1. Analytical empirical study designs that aim to test an intervention or approach to address or effect access or the use of a health service.
2. Studies where is it not possible to extract sociodemographic and outcomes data for student participants.
3. Participants less than 18 years.
4. Not university students.

**Eligibility screening for full text articles**

| **Domain** | **Criteria/ questions** | **Possible responses** | **Action** |
| --- | --- | --- | --- |
| **Population** | Are the study subjects aged at least 18 years old? | Yes – all study subjects are aged at least 18 years old | Proceed |
|  |  | Yes – a proportion of the study subjects are aged at least 18 years old | Proceed |
|  |  | No – none of the study subjects are aged at least 18 years old | Exclude |
| **Population** | Are the study subjects university students | Yes – all the study subjects are students | Proceed |
|  |  | Yes – proportion of the study subjects are students | Proceed |
|  |  | No – none of the study subjects are aged at least 18 years old | Exclude |
| **Condition** | Does the empirical study examine use of mental health services in the study population within a specified time period? | Yes | Proceed |
|  |  | No | Study not relevant |
| **Condition** | Is the study testing an intervention or approach to address or effect access or use of a health service? | Yes | Exclude |
|  |  | No | Proceed |
| **Condition** | Is it possible to extract relevant socio-demographic and outcomes data? | Yes | Proceed |
|  |  | No | Contact study authors for study level data |
| **Summary** | - Exclude if no initial response from study authors within two weeks. - If all action for all criteria is “*proceed*” include the study. | | |

**Appendix S4: Data Extraction Form (2)**

| **Study details** | | | | |
| --- | --- | --- | --- | --- |
| Reviewer – either SL or TO |  | | | |
| Study ID/Record Number - is a numeric code to identify the study from which the effect size estimate was obtained |  | | | |
| Full reference |  | | | |
| **Study Method** | | | | |
| Aims of the study – as stated in the report |  | | | |
| Study setting   - may refer to the geographic location, type of university, or country (*please give as much detail as possible*) |  | | | |
| Study design   - briefly describing the type of study design. For e.g., ‘cohort study’, ‘case-control study’, ‘cross-sectional study’, ‘observational study’, ‘longitudinal study’, ‘time-series study’, ‘pre-post study’ or ‘before and after study |  | | | |
| Subject characteristics   - Includes age, sex, country/location, sample size, diagnosis and other relevant characteristics (*e.g., place of residence, race/ethnicity/culture/language, student status, gender/sex, religion, education, socioeconomic status, social capital*) |  | | | |
| Health service description (please give as much description as possible) |  | | | |
| Dependent variable - |  | | | |
| Outcomes   - the primary outcome measured and where relevant includes associated secondary outcomes. (e.g., *Indicators could include attendances, usage, inpatient days, admissions, contacts, episodes, costs due to the receipt of treatment or attendance*). |  | | | |
| Outcome measurements   - describe the scales or tools used to measure the outcomes, and what time frame participants were asked to recall (e.g., service use within the last 12 months). |  | | | |
| Method of data analysis |  | | | |
| **Results** | | | | |
| Prevalence n/N (%) (i.e., numerator/ denominator and percentage) |  | | | |
| Proportion and 95% Confidence Intervals |  | | | |
| Incidence n/N (%) (i.e., numerator/ denominator and percentage) |  | | | |
| Proportion and 95% Confidence Intervals and duration of recruitment or the study |  | | | |
| Authors’ comments |  | | | |
| Reviewer comments |  | | | |
| **Quality appraisal (see Appendix 1) for guidance)** | **Yes** | **No** | **Unclear** | **Not Applicable** |
| 1. Was the sample frame appropriate to address the target population? |  |  |  |  |
| 1. Were study participants sampled in an appropriate way? |  |  |  |  |
| 1. Was the sample size adequate? |  |  |  |  |
| 1. Were the study subjects and setting described in detail? |  |  |  |  |
| 1. Was data analysis conducted with sufficient coverage of the identified sample? |  |  |  |  |
| 1. Were valid methods used for the identification of the condition? |  |  |  |  |
| 1. Was the condition measured in a standard, reliable way for all participants? |  |  |  |  |
| 1. Was there appropriate statistical analysis? |  |  |  |  |
| 1. Was the response rate adequate, and if not, was the low response rate managed appropriately? |  |  |  |  |
| **Quality appraisal (see Appendix 1) for guidance)** | | | | |
| 1. Was the sample frame appropriate to address the target population? |  | | | |
| 1. Were study participants sampled in an appropriate way? |  | | | |
| 1. Was the sample size adequate? |  | | | |
| 1. Were the study subjects and setting described in detail? |  | | | |
| 1. Was data analysis conducted with sufficient coverage of the identified sample? |  | | | |
| 1. Were valid methods used for the identification of the condition? |  | | | |
| 1. Was the condition measured in a standard, reliable way for all participants? |  | | | |
| 1. Was there appropriate statistical analysis? |  | | | |
| 1. Was the response rate adequate, and if not, was the low response rate managed appropriately? |  | | | |

**Additional materials**

1.        Was the sample frame appropriate to address the target population?

This question relies upon knowledge of the broader characteristics of the population of interest and the geographical area. If the study is of women with breast cancer, knowledge of at least the characteristics, demographics and medical history is needed. The term “target population” should not be taken to infer every individual from everywhere or with similar disease or exposure characteristics. Instead, give consideration to specific population characteristics in the study, including age range, gender, morbidities, medications, and other potentially influential factors. For example, a sample frame may not be appropriate to address the target population if a certain group has been used (such as those working for one organisation, or one profession) and the results then inferred to the target population (i.e. working adults).  A sample frame may be appropriate when it includes almost all the members of the target population (i.e. a census, or a complete list of participants or complete registry data).

2.        Were study participants recruited in an appropriate way?

Studies may report random sampling from a population, and the methods section should report how sampling was performed. Random probabilistic sampling from a defined subset of the population (sample frame) should be employed in most cases, however, random probabilistic sampling is not needed when everyone in the sampling frame will be included/ analysed.  For example, reporting on all the data from a good census is appropriate as a good census will identify everybody.  When using cluster sampling, such as a random sample of villages within a region, the methods need to be clearly stated as the precision of the final prevalence estimate incorporates the clustering effect. Convenience samples, such as a street survey or interviewing lots of people at a public gatherings are not considered to provide a representative sample of the base population.

3.        Was the sample size adequate?

The larger the sample, the narrower will be the confidence interval around the prevalence estimate, making the results more precise. An adequate sample size is important to ensure good precision of the final estimate. Ideally we are looking for evidence that the authors conducted a sample size calculation to determine an adequate sample size.  This will estimate how many subjects are needed to produce a reliable estimate of the measure(s) of interest. For conditions with a low prevalence, a larger sample size is needed. Also consider sample sizes for subgroup (or characteristics) analyses, and whether these are appropriate. Sometimes, the study will be large enough (as in large national surveys) whereby a sample size calculation is not required. In these cases, sample size can be considered adequate.

When there is no sample size calculation and it is not a large national survey, the reviewers may consider conducting their own sample size analysis using the following formula: (Naing et al. 2006, Daniel 1999)

n= Z2P(1-P)

d2

Where:

n= sample size

Z = Z statistic for a level of confidence

P = Expected prevalence or proportion (in proportion of one; if 20%, P = 0.2)

d = precision (in proportion of one; if 5%, d=0.05)

Ref:

Naing L, Winn T, Rusli BN. Practical issues in calculating the sample size for prevalence studies Archives of Orofacial Sciences. 2006;1:9-14.

Daniel WW. Biostatistics:  A Foundation for Analysis in the Health Sciences.

Edition. 7th ed. New York: John Wiley & Sons. 1999.

4.        Were the study subjects and setting described in detail?

Certain diseases or conditions vary in prevalence across different geographic regions and populations (e.g.  Women vs. Men, sociodemographic variables between countries).  The study sample should be described in sufficient detail so that other researchers can determine if it is comparable to the population of interest to them.

5.        Was data analysis conducted with sufficient coverage of the identified sample?

Coverage bias can occur when not all subgroups of the identified sample respond at the same rate. For instance, you may have a very high response rate overall for your study, but the response rate for a certain subgroup (i.e. older adults) may be quite low.

6.        Were valid methods used for the identification of the condition?

Here we are looking for measurement or classification bias.  Many health problems are not easily diagnosed or defined and some measures may not be capable of including or excluding appropriate levels or stages of the health problem. If the outcomes were assessed based on existing definitions or diagnostic criteria, then the answer to this question is likely to be yes. If the outcomes were assessed using observer reported, or self-reported scales, the risk of over- or under-reporting is increased, and objectivity is compromised. Importantly, determine if the measurement tools used were validated instruments as this has a significant impact on outcome assessment validity.

7.        Was the condition measured in a standard, reliable way for all participants?

Considerable judgment is required to determine the presence of some health outcomes. Having established the validity of the outcome measurement instrument (see item 6 of this scale), it is important to establish how the measurement was conducted.  Were those involved in collecting data trained or educated in the use of the instrument/s? If there was more than one data collector, were they similar in terms of level of education, clinical or research experience, or level of responsibility in the piece of research being appraised? When there was more than one observer or collector, was there comparison of results from across the observers? Was the condition measured in the same way for all participants?

8.        Was there appropriate statistical analysis?

Importantly, the numerator and denominator should be clearly reported, and percentages should be given with confidence intervals.  The methods section should be detailed enough for reviewers to identify the analytical technique used and how specific variables were measured. Additionally, it is also important to assess the appropriateness of the analytical strategy in terms of the assumptions associated with the approach as differing methods of analysis are based on differing assumptions about the data and how it will respond.

9.        Was the response rate adequate, and if not, was the low response rate managed appropriately?

A large number of dropouts, refusals or “not founds” amongst selected subjects may diminish a study’s validity, as can a low response rates for survey studies. The authors should clearly discuss the response rate and any reasons for non-response and compare persons in the study to those not in the study, particularly with regards to their socio-demographic characteristics. If reasons for non-response appear to be unrelated to the outcome measured and the characteristics of non-responders are comparable to those who do respond in the study (addressed in question 5, coverage bias), the researchers may be able to justify a more modest response rate.

**
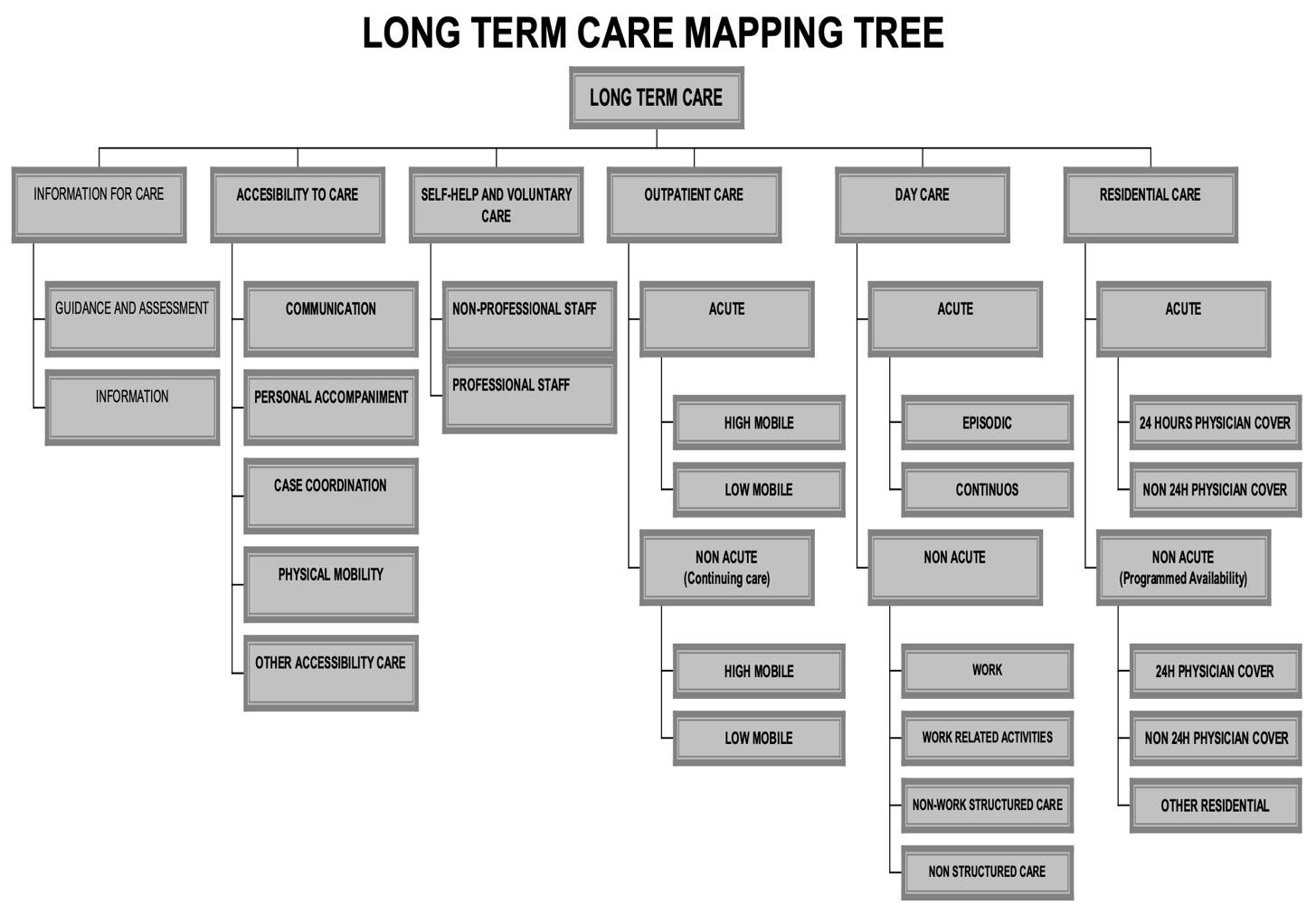
Appendix S5: Relevant Sections from the eDESDE-LTC coding framework used for coding services**, full coding framework available here: [<http://www.edesdeproject.eu/images/toolkit/DESDE-LTC_Instrument.pdf>] (3, 4)

“The aim of Section B is to produce a comprehensive categorisation of the facilities providing care teams for a defined target group in the local population, classified according to function, availability and setting.

DESDE-LTC 2.0 provides a list of care teams (SCTs) identified in each local area and the code assigned to them. This gives a quick vision of the availability of care teams and type of care teams.

DESDE-LTC 2.0 has a glossary giving definitions of all terms used in the long-term care mapping trees in the Guidelines for Coding LTC. Examples of care teams within each category and guidelines on categories that should be mutually exclusive are also given.

A coding of LTC services must also include acute care services as LTC consumers (e.g., experiencing comorbid non- communicable diseases such as cadiovascular problems, respiratory problems, mental disorders, diabetes, and cancer) are also the main users of acute care teams such as emergency and acute hospital care in the local health system

There are six mapping branches for 1) information care 2) accessibility to care; 3) self-help and volunteer care; 4) outpatient care 5) day care, and 6) residential care. However, self-help, and informal care are not included in the Care team Counting Trees as it is assumed that activity volumes are not often documented precisely.

The location in the tree of each SCT is identified by a combination of a letter and a number: (i) a capital “I”, “A”, ”S”, “O”, “D” or “R” indicates whether the care team is part of the information, accessibility, self-help, out-patient, day and/or residential trees; (ii) within these trees, each final branch is given a number.”

**“GUIDELINES FOR CODING CARE PROVISION**

SCTs are classified according to a number of descriptors (types and qualifiers), such as main branch, status of user, care typology, intensity, time of stay, and mobility. These descriptors provide a classification based on the “Main Types of Care,” including information on care, accessibility to care, self-help and volunteer care, outpatient care, day care and residential care. SCTs are arranged or organised either as a single MTC or in cluster combination of MTCs.

There are some examples of types of care that can be classified in each code. This list of examples does not pretend to be exhaustive. Some instructions are also given for situations where branches are mutually exclusive (i.e., pairs of branches where a particular care team never must be classified as part of both at the same time).

The coding framework is hierarchical. Therefore, please start with step 1, code the service in one of the five first codes. Then go to step 2 for the appropriate higher-level code. At step 2, please code from left to right.

Every care function is described in simple language and has a specific alpha-numeric code (for example: provides night accommodation for acute users in a setting with 24-medical care: R2). These codes are defined by a series of qualifiers hierarchically arranged in 6 levels (only using four):

***First Level***- **Main branch** (or main function of care: I) Information, A) Accessibility, S) Self-care and voluntary, O) Outpatient, D) Day care, R) Residential care.
***Second Level*** –**Status of user**. This level typically relates to the clinical or functional status of the users who are attended in the care setting, i.e. a crisis situation will define the care team as acute care.

***Third Level*** –**General type of care**. This level usually describes the main general typology of care (home & mobile/non-mobile, physician or non-physician cover).

***Fourth Level* – Intensity of care**. This level typically refers to the intensity of care that the care team can offer except for “residential acute care” where the third level describes whether care is provided in a registered hospital or not.”

**“STEP 1**

**MENTAL HEALTH SERVICE CODES”**

| **“I: INFORMATION SERVICES FOR CARE – *IF YES, GO TO STEP 2 INFORMATION SERVICES FOR CARE”*** |  |
| --- | --- |
| “Facilities whose main aim is to provide information and assessment to users with long term care needs. This care does not entail a subsequent monitoring/follow-up of the user.” |  |
| **“A: ACCESSIBILITY TO CARE – *IF YES, GO TO STEP 2 ACCESSIBILITY TO CARE”*** |  |
| “Facilities whose main aim (Main Type of Care- MTC) is to provide accessibility aids for users with long term care needs.” |  |
| **“S: SELF-HELP AND VOLUNTEER CARE – *IF YES, GO TO STEP 2 SELF-HELP AND VOLUNTEER CARE”*** |  |
| “The aim of these facilities is to provide users with long term care needs with support, self-help or contact, with un-paid staff that offers accessibility, information, day, outpatient and residential care (as described in other branches).” |  |
| **“O: OUTPATIENT CARE – *IF YES, GO TO STEP 2 OUTPATIENT CARE”*** |  |
| “These are facilities which (i) involve contact between staff and users for some purpose related to management of their condition and its associated clinical and social difficulties and (ii) are not provided as a part of delivery of residential or day services, as defined below.” |  |
| **“D: DAY CARE – *IF YES, GO TO STEP 2 DAY CARE”*** |  |
| “These are facilities that (i) are normally available to several users at a time (rather than delivering services to individuals one at a time); (ii) provide some combination of treatment for problems related to long-term care needs: e.g. providing a structured activity, or social contact and/or support; (iii) have regular opening hours during which they are normally available: and (iv) expect service users to stay at the facilities beyond the periods during which they have face-to-face contact with staff (i.e. the service is not simply based on individuals coming for appointments with staff and then leaving immediately after their appointments). The care delivery is usually planned in advance.” |  |
| **“R: RESIDENTIAL CARE – *IF YES, GO TO STEP 2 RESIDENTIAL CARE”*** |  |
| “Facilities that provide beds overnight for patients for a purpose related to the clinical and social management of their long term care needs - patients are not intended to sleep there solely because they have no home or are unable to reach home.” |  |

**“STEP 2**

**I: INFORMATION SERVICE FOR CARE”**

| **“I1: Guidance and Assessment.** Facilities that offer professional assessment and guidance.” |  |
| --- | --- |
| **“I2: Information.** Facilities intended exclusively to provide information to users with long term care needs.” |  |

**“A: ACCESSIBILITY FOR CARE”**

| **“A1: Communication.** Facilities which main aim is to facilitate the access to information.” |  |
| --- | --- |
| **“A2: Physical mobility.** Facilities which main aim is to facilitate the physical mobility of users with long term care needs.” |  |
| **“A3: Personal accompaniment.** Facilities which main aim is to facilitate the paid personal accompaniment by non-care professionals of users with long term care needs.” |  |
| **“A4: Case coordination.** Facilities which main aim is to facilitate the care coordination and the related accessibility to different types of services, professionals and tests by users with long term care needs.” |  |
| **“A5: Other accessibility care.** Intended to facilitate the access to care which do not include any type of direct care provision.” |  |

**“S: SELF-HELP AND VOLUNTEER CARE”**

| **“S1: NON-PROFESSIONAL STAFF.** Self-help and voluntary facilities where professionals providing assessment, interventions or support to users with long term care needs are below 60% of the total personnel. The 100% of the staff is unpaid.” |  |
| --- | --- |
| **“S2: PROFESSIONAL STAFF.** Self-help and voluntary facilities designed for users with long term care needs that regularly at least 60% of staff are trained or specifically qualified for providing assessment, intervention and support to users with long term care needs. The 100% of the staff is un-paid.” |  |

**“STEP 2**

**O: OUTPATIENT CARE”**

| **“ACUTE CARE**  These emergency facilities (i) provide assessment and initial treatment in response to a crisis, deterioration in physical or mental state, behaviour or social functioning which is related to the condition; and (ii) can usually provide a same day response during working hours or at least within 72 hours after the care demand. At least 20% of the users in the last twelve months do meet the criteria for acute outpatient care for crisis.” |  | **“High Mobility (Home & Mobile) (O1-O2)**  In high mobility or home & mobile facilities contact with users occurs in a range of settings including users’ homes, as judged most appropriate by professionals and users. For a care team to be classified as high mobility (home & mobile), at least 50% of contacts should take place away from the premises at which the care team is based.  ***Either***  **O1 24 hours** 24-hours are acute care teams which are available 24 hours a day, 7 days per week.  **O2 Limited-Hours** These facilities are not always available (opening hours less than 24 hours, 7 days per week).” |  |
| --- | --- | --- | --- |
|  |  | **“Low mobility (Low & Non-mobile) (O3-O4)**  Care teams which do not meet the criteria for high mobility (‘home & mobile’). Low mobile teams include facilities whose main purpose is non-mobile care but that also could provide different levels of mobile care always below 50% of their overall activity.  ***Either***  **Q3 24 hours** 24-hours are acute care teams which are available 24 hours a day, 7 days per week.  **O4 Limited-Hours** These facilities are not always available (opening hours less than 24 hours, 7 days per week).” |  |
| **“NON-ACUTE CARE**  These facilities provide care team users with continuing care including regular contact with a health professional, which may be long term if required. Continuing care care teams may also provide acute/emergency care on a regular basis. Only when acute care is over 20% of the ordinary activity of the care team both acute and non-acute branches should be registered.” |  | **“High Mobility (Home & Mobile) (O5-O7)**  In high mobility or home & mobile facilities contact with users occurs in a range of settings including users’ homes, as judged most appropriate by professionals and users. For a care team to be classified as high mobility (home & mobile), at least 50% of contacts should take place away from the premises at which the care team is based.  ***Either***  **O5 High intensity**  These are facilities which have the capacity to make face to face contact with users at least three times per week when clinically indicated.  **Q4 Medium intensity**  These facilities do not have the capacity to supply three times weekly contact to users, but which can provide contacts at least once a fortnight when indicated.  **O7 Low intensity**  These facilities do not have the capacity to see care team users as often as once a fortnight.” |  |
|  |  | **“Low mobility (Low & Non-mobile) (O3-O4)**  Care teams which do not meet the criteria for high mobility (‘home & mobile’). Low mobile teams include facilities whose main purpose is non-mobile care but that also could provide different levels of mobile care always below 50% of their overall activity.  ***Either***  **O5 High intensity**  These are facilities which have the capacity to make face to face contact with users at least three times per week when clinically indicated.  **Q4 Medium intensity**  These facilities do not have the capacity to supply three times weekly contact to users, but which can provide contacts at least once a fortnight when indicated.  **O7 Low intensity**  These facilities do not have the capacity to see care team users as often as once a fortnight.” |  |

**“STEP 2**

**D: DAY CARE”**

| **“ACUTE CARE**  Facilities where (i) users are regularly admitted because of a crisis or a deterioration in physical or mental state, behaviour or social functioning related to their health condition; (ii) alleviating this crisis/deterioration is the main purpose of the facility. At least 20% of the users in the last twelve months do meet the criteria for acute care for crisis.” |  | **“D0 EPISODIC ACUTE CARE**  Facilities which usually provide day care to users with a deterioration of their health state on a single or a limited number of episodes of care during a defined period of time.” |  |
| --- | --- | --- | --- |
|  |  | **“D1 CONTINUOUS ACUTE CARE**  Facilities where (i) users are regularly admitted because of a crisis or a deterioration in physical or mental state, behaviour or social functioning related to their health condition; (ii) alleviating this crisis/deterioration is the main purpose of the facility; (iii) Care is provided on a continuous base –non episodic, at least 5 days a week- during a limited period of time. These day facilities are organised to provide an alternative to hospitalisation or to accelerate discharge from inpatient units before the crisis is ended or the user is stable. Admission to the facility is usually available within less than 4 weeks from the crisis onset for user discharged from an acute residential unit (R2 or R3). At least 80% of the users in the last twelve months are admitted within less than four weeks of the crisis onset” |  |
| **“NON ACUTE (D2-D9)**  All day care facilities that do not meet the criteria for acute care for crisis.” |  | **“WORK (D2, D6)**  Work facilites which provide users with the opportunity to work for pay. These are usually sheltered work care teams or opportunities on the open labour market.  ***Either***  **D2 High intensity work care**  High intensity facilities are available for care team users who attend for at least the equivalent of four half days per week. Not all care team users need attend as frequently as this for the care team to be classified as ‘high intensity’, but it should at least be possible for them to do so.  **D6 Low intensity work care**  Facilities where users usually attend for less than the equivalent of four half days per week.” |  |
|  |  | **“WORK RELATED CARE (D3, D7)**  These are facilities where users carry out an activity which closely resembles work for which payment would be expected in the open market, but where users are not paid or are paid less than 50% of the usual local expected wage for this form of work. Where there is no minimum wage, we suggest calculating an expected level based on starting salaries for similar jobs advertised in the local press over the past month.  ***Either***  **D3 High intensity work-related care** (as in D2)  **D7 Low intensity work-related care** (as in D6)” |  |
|  |  | **“NON-WORK STRUCTURED DAY CARE (D4, D8)**  Facilities where the user is involved in structured non-work related activities for a specific period of a minimum of 25% of opening hours.These facilities provide structured activities different from work and work-related care. Such activities may include skills training, creative activities such as art or music and group work  ***Either***  **D4 High intensity structured day care** (as in D2)  **D8 Low intensity structured day care** (as in D6)” |  |
|  |  | **“OTHER DAY CARE (D5, D9)**  Facilities which satisfy the criteria for non-acute day care teams, but where work or other structured activities are not available for individual users or are only available less than 25% of the opening hours**,** so that the main functions of the care team are the provision of social contact, practical advice and/or support. For example the user is not in the centre for a minimum period of time; there is not an individual plan for their day activities; the centre cannot provide a number of places available for specific clients as registered consumers may attend spontaneously at any time and perform the structured activities that are available on that day in the centre.  ***Either***  **D5 High intensity non-structured day care** (as in D2)  **D9 Low intensity non-structured day care** (as in D6)” |  |

**“STEP 2**

**R: RESIDENTIAL”**

| **“ACUTE**  Facilities where (i) users are admitted because of a crisis, a deterioration of their physical or mental state, behaviour or social functioning which is related to their health condition,; (ii)admissions usually available within 24 hours; (iii) users usually retain their own accommodation during the admission. At least 20% of the users in the last twelve months do meet the criteria for residential acute care for crisis.” |  | **“R0: Acute, 24 hours physician cover, Non-hospital.** On acute facilities (i) users are admitted because of a crisis, a deterioration of their physical or mental state, behaviour or social functioning which is related to their health condition,; (ii)admissions usually available within 24 hours; (iii) users usually retain their own accommodation during the admission. 24 hours physician cover are facilities where there is 24-hour cover by a registered physician (including medical residents). Non-hospital facilities attend users outside the location of a registered hospital.” |  |
| --- | --- | --- | --- |
|  |  | **“R1: Acute, 24 hours physician cover, Hospital, High intensity.**  Hospitals are meso-organisations with a legal recognition in most countries. This legal recognition of registered hospitals can be used as the basis for identifying hospital SCT. In those countries where there is no legal basis for deciding what are hospital care teams and in those cases where doubt exists, care teams should be classified as hospital SCT if they have more than 20 beds and 24-hour resident physician cover. A stakeholder group and/or local or regional health officers should be consulted where there is doubt about which care teams should be viewed as hospital care teams or not. Beds to which users are admitted due to a deterioration of their physical or mental status severe enough to require continuous  surveillance during 24-hours a day, and/or to require special isolation measures. They provide very high intensity monitoring (for example intensive care units)” |  |
|  |  | **“R2: Acute, 24 hours physician cover, Hospital, Medium intensity.** Acute care facilities with 24-hours physician cover in a registered hospital where (i) users are admitted due to a deterioration of their physical or mental state, behaviour or social functioning which is related to their health condition; (ii) admissions usually available within 24 hours; (iii) users usually retain their own accommodation during the admission. As in R1 except that provide regular care (medium intensity) of surveillance and/or security for in-patient admission.” |  |
|  |  | **“R3: Acute, Non-24 hours physician cover.** Facilities without 24-hour physician cover where (i) users are admitted because of a crisis, a deterioration in their physical or mental state, behaviour or social functioning which is related to the condition; (ii) admission usually available within 24 hours; (iii) users usually retain their own accommodation.” |  |
| **“Non acute (Programmed Availability) (R4-R13)**  Residential facilities that do not satisfy the criteria for acute care. Crisis admissions are sent to other facilities routinely.” |  | **“24 hours physician cover (R4-R7)**  **Hospital: These are facilities officially registered as ‘hospitals’ at national, regional or local level.**  ***Either***  **R4 Time-limited**  These are facilities where a fixed maximum period of residence is routinely specified (temporary stay). A facility should be classified as time-limited if a maximum length of stay is fixed for at least 80% of those entering the facility.  **R6 Indefinite stay**  These facilities do not fulfil the criteria for ‘time-limited’ care teams.  **Non-hospital: These are facilities with 24-hour medical cover that are NOT officially registered as ‘hospitals’ at national, regional or local level.**  ***Either***  **R5 Time limited (as in R4).**  **R7 Indefinite stay (as in R6)”** |  |
|  |  | **“Non-24h physician cover (R8-R13)** Facilities without 24-hour physician cover where i) the residential setting belongs to the government or to a public institution ii) If the person with LTC is paying a rent this is below the 50% of the market price iii), General population has not access to this type of housing iv) there is no other type of care associated but that provided by the care team.  **Time limited (R8-R10) (as in R4)**  ***Either***  **R8 24-h support.** Facilities that provide residential care during non  working hours but where there is a procedure that guarantees that the user receives 24 hours care.  **R9 Daily support**  Members of staff are regularly on site at least five days a week for some part of the day, with responsibilities related to the monitoring and clinical and social care of the user.  **R10 Lower support**  These are facilities where the care team user resides for some purpose related to the management of his/her health condition and where there is a direct link between residing in the facility and some support from staff, but where staff are regularly present fewer than five days per week.  **Indefinite stay (R11-R13) (as in R6)**  ***Either***  **R11  24-h support** (as in R8)  **R12  Daily support** (as in R9)  **R13  Lower support** (as in R10)  **R14  Other residential care** Residential non-acute facilites not classified elsewhere. (n.b. this should only be used as a code if the service cannot be classified elsewhere).” |  |

**Appendix S6: search results**

**March 2021**

| Name of database | Date of search from: | Date of search to: | Records retrieved | Record de-duplicated |
| --- | --- | --- | --- | --- |
| **MEDLINE** | 1946 | 09-03-21 (Feb week 4 2021) | 845 | 845 |
| **PsycINFO** | 1806 | 09-03-21 (March Week 1 2021 | 1635 | 1281 |
| **Embase** | 1980 | 09-03-21 (2021 week 09) | 2450 | 1783 |
| **CINAHL plus** | 1990 | 09-03-21 | 761 | 432 |
| **ERIC** | 1912 | 09-03-21 | 767 | 603 |
|  |  | Total | **6488** |  |
|  |  | De-duplicated |  | **4944** |

**November 2021**

| Name of database | Date of search from: | Date of search to: | Records retrieved | Record de-duplicated |
| --- | --- | --- | --- | --- |
| **MEDLINE** | 2021 | 2021 (November 02, 2021) | 126 | 32 |
| **PsycINFO** | 2021 | 2021 (November week 1) | 124 | 124 |
| **Embase** | 2021 | 2021 (2021 week 43) | 213 | 182 |
| **CINAHL plus** | 2021-01-01 | 2021-12-31 | 70 | 32 |
| **ERIC** | 2021-01-01 | 2021-12-31 | 20 | 16 |
|  |  | Total | **553** |  |
|  |  | De-duplicated |  | **386** |

**November 2021**

| Name of database | Date of search from | Date of search to: | Records retrieved | Records de-duplicated |
| --- | --- | --- | --- | --- |
| **MEDLINE** | 2021 | 2022 (August 23, 2022) | 275 | 154 |
| **PsycINFO** | 2021 | 2022 (August week 4) | 296 | 121 |
| **Embase** | 2021 | 2022 (2022 week 34) | 490 | 161 |
| **CINAHL plus** | 2021-01-01 | 2022-08-31 | 151 | 46 |
| **ERIC** | 2021-01-01 | 2022-08-31 | 49 | 22 |
|  |  | Total | **1251** |  |
|  |  | De-duplicated |  | **504** |

**Appendix S7: Quality Appaisal (2)**

| **Author (year)** | **Joanna Briggs Institute (JBI) Tool Questions** | | | | | | | | | **Quality Appraisal rating** |
| --- | --- | --- | --- | --- | --- | --- | --- | --- | --- | --- |
|  | **Was the sample frame appropriate to address the target population?** | **Were the study participants sampled in an appropriate way?** | **Was the sample size adequate?** | **Were the study subjects and setting described in detail?** | **Was the data analysis conducted with sufficient coverage of the identified sample?** | **Were valid methods used for the identification of the condition?** | **Was the condition measured in a standard, reliable (test) way for all participants?** | **Was there appropriate statistical analysis?** | **Was the response rate adequate, and if not was the low response rate managed appropriately?** |  |
| Yorgason *et al* (2008) | Yes | Yes | Yes | No | No | No | No | No | Unclear | Low |
| Xiao *et al* (2017) | Yes | Yes | Yes | Yes | Yes | No | No | No | Unclear | Moderate |
| Williams *et al* (2021) | Yes | Yes | Yes | No | Yes | No | No | No | Unclear | Moderate |
| Turner *et al* (2015) | Yes | Yes | Yes | No | Yes | No | No | Yes | Unclear | Moderate |
| Sontag-Padilla *et al* (2016) | Yes | Yes | Yes | Yes | Yes | No | No | No | No | Moderate |
| Smith *et al* (2021) | Yes | No | Yes | No | Yes | No | No | Yes | No | Moderate |
| Sifat *et al* (2022) | Yes | Yes | Yes | Yes | Yes | No | No | No | Unclear | Moderate |
| Ryan *et al* (2017) | Yes | Yes | Yes | Yes | Yes | No | No | No | No | Moderate |
| Romano *et al* (2022) | Yes | Yes | Yes | Yes | Yes | Yes | Yes | No | Yes | High |
| Rice (2015) | Yes | Yes | Yes | Yes | Yes | No | No | No | No | Moderate |
| Nilsson *et al* (2004) | Yes | Unclear | Unclear | No | Yes | Unclear | Unclear | No | Yes | Low |
| Nash *et al* (2017) | Yes | Yes | Yes | Yes | No | Yes | Unclear | Unclear | No | Moderate |
| Lu *et al* (2014) | Yes | No | Unclear | Yes | Yes | Yes | No | No | No | Moderate |
| Liu *et al* (2017) | Yes | Yes | Yes | No | Yes | No | No | No | Unclear | Moderate |
| Lipson *et al* (2019) | Yes | Yes | Yes | No | Yes | No | No | No | Yes | Moderate |
| Lipson *et al* (2021) | Yes | Yes | Yes | No | Yes | No | No | No | Yes | Moderate |
| Linden *et al* (2021) | Yes | Yes | Yes | Yes | Yes | No | No | No | No | Moderate |
| Lee *et al* (2021) | Yes | Yes | Yes | Yes | Yes | No | No | No | Yes | Moderate |
| Leao *et al* (2011) | Yes | Yes | Yes | Yes | Yes | No | No | No | Yes | Moderate |
| Kerr *et al* (2013) | No | Yes | Yes | No | Yes | No | No | No | No | Low |
| Karaffa *et al* (2019) | No | Yes | Yes | Yes | Unclear | No | No | No | No | Low |
| Jennings *et al* (2015) | No | Yes | Yes | No | Unclear | No | No | Yes | Unclear | Low |
| Jardon *et al* (2022) | Yes | Yes | Yes | Yes | Yes | No | No | No | Yes | Moderate |
| Huang *et al* (2020) | Yes | Yes | Yes | Yes | Unclear | No | No | No | Yes | Moderate |
| Han *et al* (2016) | Yes | Yes | Yes | Yes | Yes | No | No | No | Yes | Moderate |
| Giusti *et al* (2020) | Yes | Yes | Unclear | Yes | Unclear | No | No | No | Yes | Moderate |
| Gebreegziabher *et al* (2019) | Yes | Yes | Yes | Yes | Yes | Yes | Yes | No | Yes | High |
| Fischbein *et al* (2019) | Yes | Yes | Yes | Yes | Yes | Yes | No | No | Yes | High |
| Eisenberg *et al* (2012) | Yes | Yes | Yes | No | Yes | Yes | No | No | Yes | Moderate |
| Eisenberg *et al* (2011a) | Yes | Yes | Yes | Yes | Yes | Yes | Yes | No | Yes | High |
| Eisenberg *et al* (2011b) | Yes | Yes | Yes | Yes | Yes | Yes | Yes | No | Yes | High |
| Eisenberg *et al* (2007) | Yes | Yes | Yes | No | Yes | Yes | Yes | No | Yes | High |
| Dyrbye *et al* (2015) | Yes | Yes | Yes | Yes | No | Yes | Yes | No | Yes | High |
| Dunbar *et al* (2017) | Yes | Yes | Yes | Yes | No | No | No | No | No | Moderate |
| Cranford *et al* (2008) | Yes | Yes | Yes | Yes | Yes | Yes | No | No | Yes | High |
| Connor *et al* (2022) | Yes | Yes | Yes | Yes | No | No | No | No | Unclear | Moderate |
| Chang *et al* (2013) | Yes | Yes | Yes | Yes | Unclear | No | No | No | Unclear | Moderate |
| Bourdon *et al* (2020) | Yes | Yes | Yes | Yes | Yes | No | No | No | Unclear | Moderate |
| Bonar *et al* (2015) | Yes | Yes | Unclear | Yes | Yes | No | No | No | Yes | Moderate |
| Bastos *et al* (2022) | Unclear | Unclear | Yes | No | No | No | No | No | No | Low |
| Baams *et al* (2018) | Yes | Yes | Yes | Yes | Unclear | No | No | Yes | Unclear | Moderate |
| Artime *et al* (2019) | Yes | Yes | Yes | Yes | Unclear | No | No | No | Yes | Moderate |
| Albright *et al* (2020) | Yes | Yes | Yes | No | Unclear | No | No | No | Unclear | Low |

**Appendix S8: Overall service use**

**8.1: Subgroup analyses**

***Table 8.1.i: Subgroup analysis – population group***

| **K** | **Variable** | **Proportion** | **95% CI** | Ι^2^ | *p*subgroup |
| --- | --- | --- | --- | --- | --- |
| 3 | General | 0.32 | 0.12; 0.61 | 100% | 0.91 |
| 3 | At risk | 0.35 | 0.18; 0.57 | 96.4% |  |
| 3 | Subgroup | 0.37 | 0.02; 0.94 | 99.7% |  |

***Table 8.1.ii: Subgroup analysis – treatment type***

| **K** | **Variable** | **Proportion** | **95% CI** | Ι^2^ | *p*subgroup |
| --- | --- | --- | --- | --- | --- |
| 8 | Any | 0.34 | 0.20; 0.52 | 99.8% | 0.13 |
| 1 | Low intensity | 0.45 | 0.44; 0.45 | N/A |  |

***Table 8.1.iii: Subgroup analysis – service location***

| **K** | **Variable** | **Proportion** | **95% CI** | Ι^2^ | *p*subgroup |
| --- | --- | --- | --- | --- | --- |
| 7 | Either | 0.42 | 0.29; 0.56 | 99.9% | 0.006 |
| 2 | Off-campus | 0.16 | 0.01; 0.98 | 99.9% |  |

***Table 8.1.iv: Subgroup analysis – time-period***

| **K** | **Variable** | **Proportion** | **95% CI** | Ι^2^ | *p*subgroup |
| --- | --- | --- | --- | --- | --- |
| 4 | Twelve months | 0.35 | 0.24; 0.48 | 95.3% | <.001 |
| 2 | While at university | 0.13 | 0.01; 0.90 | 99.2% |  |
| 3 | Ever | 0.54 | 0.27; 0.79 | 98.4% |  |

**8.2: Meta-regressions**

***Table 8.2.i: Meta-regression – population group***

| **K** | **Variable** | **Coefficient** | **SE** | **p-value** | **95% CI** | Ι^^2^ |
| --- | --- | --- | --- | --- | --- | --- |
| 9 | At risk^a^ | 0.17 | 0.68 | 0.82 | -1.49; 1.82 | 99.97% |
|  | Subgroup^a^ | 0.23 | 0.68 | 0.74 | -1.42; 1.88 |  |

*^a^Reference category=general population*

***Table 8.2.ii: Meta-regression – treatment type***

| **K** | **Variable** | **Coefficient** | **SE** | **p-value** | **95% CI** | Ι^^2^ |
| --- | --- | --- | --- | --- | --- | --- |
| 9 | Low intensity^a^ | 0.47 | 0.87 | 0.61 | -1.58; 2.51 | 99.91% |

*^a^Reference category=any treatment*

***Table 8.2.iii: Meta-regression – publication year***

| **K** | **Variable** | **Coefficient** | **SE** | **p-value** | **95% CI** | Ι^^2^ |
| --- | --- | --- | --- | --- | --- | --- |
| 9 | Year^a^ | -0.11 | 0.09 | 0.28 | -0.33; 0.11 | 99.95% |

*^a^Reference category=2015*

***Table 8.2.iv: Meta-regression – country***

| **K** | **Variable** | **Coefficient** | **SE** | **p-value** | **95% CI** | Ι^^2^ |
| --- | --- | --- | --- | --- | --- | --- |
| 9 | Canada^a^ | -0.16 | 1.13 | 0.89 | -2.90; 2.59 | 99.92% |
|  | USA^a^ | -0.71 | 0.86 | 0.44 | -2.80; 1.39 |  |

^a^*Reference category=Australia*

***Table 8.2.v: Meta-regression – service location***

| **K** | **Variable** | **Coefficient** | **SE** | **p-value** | **95% CI** | Ι^^2^ |
| --- | --- | --- | --- | --- | --- | --- |
| 9 | Off-campus^a^ | -1.35 | 0.49 | 0.03 | -2.52; -0.18 | 99.95% |

*^a^Reference category=either on or off campus*

***Table 8.2.vi: Meta-regression – time-period***

| **K** | **Variable** | **Coefficient** | **SE** | **p-value** | **95% CI** | Ι^^2^ |
| --- | --- | --- | --- | --- | --- | --- |
| 9 | Months^a^ | 0.01 | 0.01 | 0.02 | -0.01; 0.01 | 99.82% |

*^a^ Reference category=twelve months*

**Appendix S9: Overall outpatient service use**

**9.1: Subgroup analyses**

***Table 9.1.i: Subgroup analysis – population group***

| **K** | **Variable** | **Proportion** | **95% CI** | Ι^2^ | *p*subgroup |
| --- | --- | --- | --- | --- | --- |
| 8 | General | 0.10 | 0.08; 0.14 | 99.4% | <.001 |
| 12 | At risk | 0.36 | 0.26; 0.49 | 99.7% |  |
| 6 | Subgroup | 0.17 | 0.06; 0.39 | 98.7% |  |

***Table 9.1.ii: Subgroup analysis – treatment type***

| **K** | **Variable** | **Proportion** | **95% CI** | Ι^2^ | *p*subgroup |
| --- | --- | --- | --- | --- | --- |
| 12 | Any | 0.29 | 0.17; 0.55 | 99.3% | 0.09 |
| 4 | High intensity | 0.25 | 0.16; 0.38 | 99.8% |  |
| 10 | Low intensity | 0.14 | 0.07; 0.25 | 100% |  |

***Table 9.1.iii: Subgroup analysis – study design***

| **K** | **Variable** | **Proportion** | **95% CI** | Ι^2^ | *p*subgroup |
| --- | --- | --- | --- | --- | --- |
| 1 | Cohort | 0.05 | 0.05; 0.06 | N/A | <.001 |
| 20 | Cross sectional | 0.25 | 0.18; 0.34 | 99.9% |  |
| 2 | Mixed method | 0.36 | 0.01; 0.99 | 87.8% |  |
| 3 | Secondary data analysis | 0.08 | 0.01; 0.53 | 100.0% |  |

***Table 9.1.iv: Subgroup analysis – service location***

| **K** | **Variable** | **Proportion** | **95% CI** | Ι^2^ | *p*subgroup |
| --- | --- | --- | --- | --- | --- |
| 12 | Either | 0.33 | 0.22; 0.47 | 99.2% | 0.0054 |
| 14 | On-campus | 0.14 | 0.09; 0.22 | 99.9% |  |

***Table 9.1.v: Subgroup analysis – time-period***

| **K** | **Variable** | **Proportion** | **95% CI** | Ι^2^ | *p*subgroup |
| --- | --- | --- | --- | --- | --- |
| 2 | Current | 0.48 | 0.02; 0.99 | 62.2% | 0.06 |
| 1 | Two months | 0.69 | 0.27; 0.93 | N/A |  |
| 7 | Twelve months | 0.18 | 0.09; 0.33 | 99.9% |  |
| 12 | While at university | 0.17 | 0.10; 0.26 | 99.9% |  |
| 4 | Ever | 0.27 | 0.08; 0.62 | 99.5% |  |

**9.2: Meta-regressions**

***Table 9.2.i: Meta-regression – population group***

| **K** | **Variable** | **Coefficient** | **SE** | **p-value** | **95% CI** | Ι^^2^ |
| --- | --- | --- | --- | --- | --- | --- |
| 26 | At risk^a^ | 1.62 | 0.36 | <.001 | 0.88; 2.37 | 99.89% |
|  | Subgroup^a^ | 0.58 | 0.42 | 0.18 | -0.29; 1.46 |  |

*^a^ Reference category=general population*

***Table 9.2.ii: Meta-regression – treatment type***

| **K** | **Variable** | **Coefficient** | **SE** | **p-value** | **95% CI** | Ι^^2^ |
| --- | --- | --- | --- | --- | --- | --- |
| 26 | High intensity^a^ | -0.19 | 0.57 | 0.74 | -1.36; 0.98 | 99.97% |
|  | Low intensity^a^ | -0.91 | 0.42 | 0.04 | -1.78; -0.04 |  |

*^a^ Reference category=any treatment*

***Table 9.2.iii: Meta-regression – publication year***

| **K** | **Variable** | **Coefficient** | **SE** | **p-value** | **95% CI** | Ι^^2^ |
| --- | --- | --- | --- | --- | --- | --- |
| 26 | Year^a^ | 0.08 | 0.04 | 0.08 | -0.01; 0.17 | 99.95% |

*^a^ Reference category=2004*

***Table 9.2.iv: Meta-regression – study design***

| **K** | **Variable** | **Coefficient** | **SE** | **p-value** | **95% CI** | Ι^^2^ |
| --- | --- | --- | --- | --- | --- | --- |
| 26 | Cross-sectional^a^ | 1.82 | 0.91 | 0.06 | -0.07; 3.71 | 99.94% |
|  | Mixed-method^a^ | 2.41 | 1.12 | 0.04 | 0.08; 4.73 |  |
|  | Secondary data analysis^a^ | 0.43 | 1.03 | 0.68 | -1.70; 2.55 |  |

*^a^ Reference category=Cohort study*

***Table 9.2.v: Meta-regression – country***

| **K** | **Variable** | **Coefficient** | **SE** | **p-value** | **95% CI** | Ι^^2^ |
| --- | --- | --- | --- | --- | --- | --- |
| 26 | Bangladesh^a^ | -3.69 | 1.22 | 0.007 | -6.22; -1.15 | 99.97% |
|  | Brazil^a^ | -1.86 | 1.05 | 0.09 | -4.04; 0.33 |  |
|  | China^a^ | -4.03 | 1.20 | 0.003 | -6.53; -1.54 |  |
|  | Italy^a^ | -2.37 | 1.22 | 0.07 | -4.91; 0.18 |  |
|  | USA^a^ | -2.44 | 0.88 | 0.01 | -4.27; -0.61 |  |

^a^ *Reference category=Australia*

***Table 9.2.vi: Meta-regression – service location***

| **K** | **Variable** | **Coefficient** | **SE** | **p-value** | **95% CI** | Ι^^2^ |
| --- | --- | --- | --- | --- | --- | --- |
| 26 | On-campus^a^ | -1.10 | 0.36 | 0.005 | -1.85; -0.36 | 99.96% |

*^a^ Reference category=either on or off campus*

***Table 9.2.vii: Meta-regression – time-period***

| **K** | **Variable** | **Coefficient** | **SE** | **p-value** | **95% CI** | Ι^^2^ |
| --- | --- | --- | --- | --- | --- | --- |
| 26 | Months^a^ | 0.01 | 0.01 | 0.66 | -0.01; 0.01 | 99.97% |

*^a^ Reference category=0.5 months*

**Appendix S10: Overall residential service use**

**10.1: Subgroup analyses**

***Table 10.1.i: Subgroup analysis – population group***

| **K** | **Variable** | **Proportion** | **95% CI** | Ι^2^ | *p*subgroup |
| --- | --- | --- | --- | --- | --- |
| 1 | General | 0.01 | 0.01; 0.01 | N/A | <.001 |
| 4 | At risk | 0.04 | 0.03; 0.05 | 99.7% |  |
| 2 | Subgroup | 0.04 | 0.02; 0.89 | 73.3% |  |

***Table 10.1.ii: Subgroup analysis – time-period***

| **K** | **Variable** | **Proportion** | **95% CI** | Ι^2^ | *p*subgroup |
| --- | --- | --- | --- | --- | --- |
| 3 | Twelve months | 0.02 | 0.01; 0.11 | 99.7% | 0.19 |
| 4 | Ever | 0.04 | 0.02; 0.05 | 80.4% |  |

***Table 10.1.iii: Subgroup analysis – service location***

| **K** | **Variable** | **Proportion** | **95% CI** | Ι^2^ | *p*subgroup |
| --- | --- | --- | --- | --- | --- |
| 3 | Either | 0.03 | 0.01; 0.17 | 99.5% | 0.82 |
| 4 | Off-campus | 0.03 | 0.03; 0.04 | 58.0% |  |

**10.2: Meta-regressions**

***Table 10.2.i: Meta-regression – population group***

| **K** | **Variable** | **Coefficient** | **SE** | **p-value** | **95% CI** | Ι^^2^ |
| --- | --- | --- | --- | --- | --- | --- |
| 7 | At risk^a^ | 1.29 | 0.16 | 0.001 | 0.84; 1.73 | 75.17% |
|  | Subgroup^a^ | 1.50 | 0.25 | 0.004 | 0.80; 2.21 |  |

*^a^Reference category=general population*

***Table 10.2.ii: Meta-regression – time-period***

| **K** | **Variable** | **Coefficient** | **SE** | **p-value** | **95% CI** | Ι^^2^ |
| --- | --- | --- | --- | --- | --- | --- |
| 7 | Months^a^ | 0.01 | 0.01 | 0.18 | -0.01; 0.01 | 97.82% |

*^a^Reference category=twelve months*

***Table 10.2.iii: Meta-regression – publication year***

| **K** | **Variable** | **Coefficient** | **SE** | **p-value** | **95% CI** | Ι^^2^ |
| --- | --- | --- | --- | --- | --- | --- |
| 7 | Year^a^ | -0.16 | 0.09 | 0.25 | -0.34; 0.11 | 97.04% |

*^a^Reference category=2015*

***Table 10.2.iv: Meta-regression – service location***

| **K** | **Variable** | **Coefficient** | **SE** | **p-value** | **95% CI** | Ι^^2^ |
| --- | --- | --- | --- | --- | --- | --- |
| 7 | Off-campus^a^ | 0.05 | 0.43 | 0.92 | -1.07; 1.16 | 97.9% |

*^a^Reference category=either on or off campus*

**Appendix S11: Sensitivity analyses**

**11.1: Subgroup analyses – service type**

***Table 11.1.i: Subgroup analysis – service type excluding lifetime use***

| **K** | **Variable** | **Proportion** | **95% CI** | *Ι* *^2^_level 2_* | *Ι* *^2^_level 3_* | *p*subgroup |
| --- | --- | --- | --- | --- | --- | --- |
| 6 | Overall | 0.30 | 0.17; 0.46 | 98% | 0% | <.001 |
| 22 | Outpatient | 0.20 | 0.14; 0.28 |  |  |  |
| 3 | Residential | 0.02 | 0.01; 0.05 |  |  |  |

***Table 11.1.ii: Subgroup analysis – service type excluding low quality studies***

| **K** | **Variable** | **Proportion** | **95% CI** | *Ι* *^2^_level 2_* | *Ι* *^2^_level 3_* | *p*subgroup |
| --- | --- | --- | --- | --- | --- | --- |
| 8 | Overall | 0.31 | 0.20; 0.45 | 98.5% | 0% | <.001 |
| 21 | Outpatient | 0.22 | 0.16; 0.30 |  |  |  |
| 6 | Residential | 0.03 | 0.02; 0.05 |  |  |  |

***Table 11.1.iii: Subgroup analysis – service type excluding influential cases and outliers***

| **K** | **Variable** | **Proportion** | **95% CI** | *Ι* *^2^_level 2_* | *Ι* *^2^_level 3_* | *p*subgroup |
| --- | --- | --- | --- | --- | --- | --- |
| 4 | Overall | 0.38 | 0.24; 0.54 | 96.2% | 0% | <.001 |
| 13 | Outpatient | 0.16 | 0.11; 0.23 |  |  |  |
| 6 | Residential | 0.04 | 0.03; 0.05 |  |  |  |

**11.2: Meta-regressions – service type**

***Table 11.2.i: Meta-regression – service type excluding lifetime use***

| **K** | **Variable** | **Coefficient** | **SE** | **p-value** | **95% CI** |
| --- | --- | --- | --- | --- | --- |
| 31 | Outpatient^a^ | -0.54 | 0.34 | 0.12 | -1.24; 0.16 |
|  | Residential^a^ | -3.02 | 0.49 | <.001 | -4.03; -2.02 |

*^a^ Reference group=overall*

***Table 11.2.ii: Meta-regression – service type excluding low-quality studies***

| **K** | **Variable** | **Coefficient** | **SE** | **p-value** | **95% CI** |
| --- | --- | --- | --- | --- | --- |
| 35 | Outpatient^a^ | -0.48 | 0.34 | 0.16 | -1.17; -0.21 |
|  | Residential^a^ | -2.83 | 0.44 | <.001 | -3.73; -1.92 |

*^a^ Reference group=overall*

***Table 11.2.iii: Meta-regression – service type excluding influential cases and outliers***

| **K** | **Variable** | **Coefficient** | **SE** | **p-value** | **95% CI** |
| --- | --- | --- | --- | --- | --- |
| 23 | Outpatient^a^ | -1.17 | 0.35 | .003 | -1.90; -0.45 |
|  | Residential^a^ | -2.84 | 0.40 | <.001 | -3.67; -2.02 |

*^a^ Reference group=overall*

**Appendix S12: Sensitivity analyses – overall service use**

**12.1: Subgroup analyses**

***Table 12.1.i: Subgroup analyses – service location excluding lifetime use***

| **K** | **Variable** | **Proportion** | **95% CI** | Ι^2^ | *p*subgroup |
| --- | --- | --- | --- | --- | --- |
| 4 | Either | 0.34 | 0.19; 0.52 | 99.9% | 0.04 |
| 2 | Off-campus | 0.16 | 0.01; 0.99 | N/A |  |

***Table 12.2.ii: Subgroup analyses – treatment type excluding low-quality studies***

| **K** | **Variable** | **Proportion** | **95% CI** | Ι^2^ | *p*subgroup |
| --- | --- | --- | --- | --- | --- |
| 7 | Any | 0.29 | 0.18; 0.45 | 99.8% | 0.01 |
| 1 | Low intensity | 0.45 | 0.44; 0.45 | N/A |  |

***Table 12.3.iii: Subgroup analyses – service location excluding outliers and influential cases***

| **K** | **Variable** | **Proportion** | **95% CI** | Ι^2^ | *p*subgroup |
| --- | --- | --- | --- | --- | --- |
| 3 | Either | 0.42 | 0.28; 0.59 | 82.8% | <.001 |
| 1 | Off-campus | 0.26 | 0.23; 0.28 | N/A |  |

**12.2: Meta-regressions**

***Table 12.2.i: Meta-regressions – service location excluding lifetime use***

| **K** | **Variable** | **Coefficient** | **SE** | **p-value** | **95% CI** | Ι^^2^ |
| --- | --- | --- | --- | --- | --- | --- |
| 6 | Off-campus^a^ | -0.99 | 0.45 | 0.09 | -2.23; 0.25 | 99.86% |

*^a^ Reference group=either on or off campus*

***Table 12.2.ii: Meta-regressions – treatment type excluding low-quality studies***

| **K** | **Variable** | **Coefficient** | **SE** | **p-value** | **95% CI** | Ι^^2^ |
| --- | --- | --- | --- | --- | --- | --- |
| 8 | Low intensity^a^ | 0.68 | 0.71 | 0.37 | -1.05; 2.41 | 99.88% |

*^a^ Reference group=any treatment*

***Table 12.2.iii: Meta-regressions – service location excluding outliers and influential cases***

| **K** | **Variable** | **Coefficient** | **SE** | **p-value** | **95% CI** | Ι^^2^ |
| --- | --- | --- | --- | --- | --- | --- |
| 4 | Off-campus^a^ | -0.73 | 0.22 | 0.08 | -1.67; 0.21 | 56.24% |

**Appendix S13: Sensitivity analyses – overall outpatient service use**

**13.1: Subgroup analyses**

***Table 13.1.i: Subgroup analyses – treatment received excluding lifetime use***

| **K** | **Variable** | **Proportion** | **95% CI** | Ι^2^ | *p*subgroup |
| --- | --- | --- | --- | --- | --- |
| 11 | Any | 0.29 | 0.16; 0.47 | 99.4% | 0.005 |
| 4 | High intensity | 0.25 | 0.16; 0.37 | 99.8% |  |
| 7 | Low intensity | 0.10 | 0.05; 0.20 | 100% |  |

***Table 13.1.ii: Subgroup analyses – treatment received excluding low-quality studies***

| **K** | **Variable** | **Proportion** | **95% CI** | Ι^2^ | *p*subgroup |
| --- | --- | --- | --- | --- | --- |
| 9 | Any | 0.33 | 0.13; 0.44 | 99.4% | 0.359 |
| 4 | High intensity | 0.25 | 0.16; 0.37 | 99.8% |  |
| 8 | Low intensity | 0.17 | 0.09; 0.29 | 100% |  |

***Table 13.1.iii: Subgroup analyses – service location excluding lifetime use***

| **K** | **Variable** | **Proportion** | **95% CI** | Ι^2^ | *p*subgroup |
| --- | --- | --- | --- | --- | --- |
| 9 | Either | 0.34 | 0.17; 0.56 | 98.2% | 0.01 |
| 13 | On-campus | 0.14 | 0.09; 0.22 | 99.9% |  |

*^a^ Reference group=either on or off campus*

***Table 13.1.iv: Subgroup analyses – service location excluding low-quality studies***

| **K** | **Variable** | **Proportion** | **95% CI** | Ι^2^ | *p*subgroup |
| --- | --- | --- | --- | --- | --- |
| 9 | Either | 0.31 | 0.17; 0.50 | 99.4% | 0.03 |
| 12 | On-campus | 0.16 | 0.11; 0.22 | 99.9% |  |

*^a^ Reference group=either on or off campus*

***Table 13.1.v: Subgroup analyses – treatment type excluding outliers and influential cases***

| **K** | **Variable** | **Proportion** | **95% CI** | Ι^2^ | *p*subgroup |
| --- | --- | --- | --- | --- | --- |
| 6 | Any | 0.17 | 0.11; 0.24 | 93.8% | 0.21 |
| 3 | High intensity | 0.21 | 0.15; 0.29 | 95.8% |  |
| 4 | Low intensity | 0.12 | 0.02; 0.45 | 99.0% |  |

*^a^ Reference group=either on or off campus*

***Table 13.1.vi: Subgroup analyses – service location excluding outliers and influential cases***

| **K** | **Variable** | **Proportion** | **95% CI** | Ι^2^ | *p*subgroup |
| --- | --- | --- | --- | --- | --- |
| 5 | Either | 0.16 | 0.10; 0.25 | 94.9% | 0.86 |
| 8 | On-campus | 0.17 | 0.09; 0.29 | 98.1% |  |

*^a^ Reference group=either on or off campus*

**13.2: Meta-regressions**

***Table 13.2.i: Meta-regressions – treatment type excluding lifetime use***

| **K** | **Variable** | **Coefficient** | **SE** | **p-value** | **95% CI** | Ι^^2^ |
| --- | --- | --- | --- | --- | --- | --- |
| 22 | High intensity^a^ | -0.21 | 0.55 | 0.70 | -1.38; 0.94 | 99.96% |
|  | Low intensity^a^ | -1.34 | 0.46 | 0.009 | -2.31; -0.38 |  |

*^a^ Reference group=any treatment*

***Table 13.2.ii: Meta-regressions – treatment received excluding low-quality studies***

| **K** | **Variable** | **Coefficient** | **SE** | **p-value** | **95% CI** | Ι^^2^ |
| --- | --- | --- | --- | --- | --- | --- |
| 21 | High intensity^a^ | -0.02 | 0.53 | 0.96 | -1.14; 1.09 | 99.97% |
|  | Low intensity^a^ | -0.50 | 0.43 | 0.26 | -1.40; 0.41 |  |

*^a^ Reference group=any treatment*

***Table 13.2.iii: Meta-regressions – service location excluding lifetime use***

| **K** | **Variable** | **Coefficient** | **SE** | **p-value** | **95% CI** | Ι^^2^ |
| --- | --- | --- | --- | --- | --- | --- |
| 22 | On-campus^a^ | -1.12 | 0.43 | 0.02 | -2.02; -0.22 | 99.96% |

*^a^ Reference group=either on or off campus*

***Table 13.2.iv: Meta-regressions – service location excluding low-quality studies***

| **K** | **Variable** | **Coefficient** | **SE** | **p-value** | **95% CI** | Ι^^2^ |
| --- | --- | --- | --- | --- | --- | --- |
| 21 | On-campus^a^ | -0.85 | 0.36 | 0.03 | -1.60; -0.09 | 99.96% |

*^a^ Reference group=either on or off campus*

***Table 13.2.v: Meta-regressions – treatment type excluding outliers and influential cases***

| **K** | **Variable** | **Coefficient** | **SE** | **p-value** | **95% CI** | Ι^^2^ |
| --- | --- | --- | --- | --- | --- | --- |
| 13 | High intensity^a^ | 0.30 | 0.48 | 0.56 | -0.79; 1.39 | 99.42% |
|  | Low intensity^a^ | -0.40 | 0.45 | 0.40 | -1.40; 0.61 |  |

*^a^ Reference group=any treatment*

***Table 13.2.vi: Meta-regressions – service location excluding outliers and influential cases***

| **K** | **Variable** | **Coefficient** | **SE** | **p-value** | **95% CI** | Ι^^2^ |
| --- | --- | --- | --- | --- | --- | --- |
| 13 | On-campus^a^ | -0.07 | 0.43 | 0.87 | -0.87; 1.01 | 99.8% |

*^a^ Reference group=either on or off campus*

**Appendix S14: Specific service use (multiple DESDE categories) analyses**

**14.1 Subgroup analyses**

***Table 14.1.i: Subgroup analysis – population group***

| **K** | **Variable** | **Proportion** | **95% CI** | *Ι* *^2^_level 2_* | *Ι* *^2^_level 3_* | *p*subgroup |
| --- | --- | --- | --- | --- | --- | --- |
| 8 | General | 0.19 | 0.08; 0.38 | 13.8% | 82.9% | 0.958 |
| 9 | At risk | 0.22 | 0.09; 0.46 |  |  |  |
| 6 | Subgroup | 0.21 | 0.07; 0.49 |  |  |  |

***Table 14.1.ii: Subgroup analysis – treatment type***

| **K** | **Variable** | **Proportion** | **95% CI** | *Ι* *^2^_level 2_* | *Ι* *^2^_level 3_* | *p*subgroup |
| --- | --- | --- | --- | --- | --- | --- |
| 8 | Any | 0.33 | 0.17; 0.55 | 13.8% | 82.9% | 0.17 |
| 9 | High intensity | 0.17 | 0.09; 0.28 |  |  |  |
| 6 | Specialist | 0.14 | 0.08; 0.26 |  |  |  |

***Table 14.1.iii: Subgroup analysis – country***

| **K** | **Variable** | **Proportion** | **95% CI** | *Ι* *^2^_level 2_* | *Ι* *^2^_level 3_* | *p*subgroup |
| --- | --- | --- | --- | --- | --- | --- |
| 2 | Australia | 0.06 | 0.01; 0.61 | 13.8% | 82.9% | 0.31 |
| 2 | Canada | 0.16 | 0.02; 0.75 |  |  |  |
| 17 | USA | 0.25 | 0.18; 0.35 |  |  |  |

***Table 14.1.iv: Subgroup analysis – service location***

| **K** | **Variable** | **Proportion** | **95% CI** | *Ι* *^2^_level 2_* | *Ι* *^2^_level 3_* | *p*subgroup |
| --- | --- | --- | --- | --- | --- | --- |
| 16 | Either | 0.15 | 0.10; 0.22 | 13.8% | 82.9% | 0.004 |
| 4 | Off-campus | 0.30 | 0.16; 0.48 |  |  |  |
| 3 | On-campus | 0.51 | 0.31; 0.72 |  |  |  |

***Table 14.1.v: Subgroup analysis – time-period***

| **K** | **Variable** | **Proportion** | **95% CI** | *Ι^2^_level 2_* | *Ι^2^_level 2_* | *p*subgroup |
| --- | --- | --- | --- | --- | --- | --- |
| 7 | Twelve months | 0.22 | 0.09; 0.46 | 13.8% | 82.9% | 0.69 |
| 3 | While at university | 0.27 | 0.07; 0.63 |  |  |  |
| 13 | Ever | 0.18 | 0.08; 0.34 |  |  |  |

**14.2 Meta-regressions**

***Table 14.2.i: Meta-regression – population group***

| **K** | **Variable** | **Coefficient** | **SE** | **p-value** | **95% CI** | **Test for Residual Heterogeneity** |
| --- | --- | --- | --- | --- | --- | --- |
| 23 | At risk^a^ | -0.20 | 0.70 | 0.78 | -1.26; 1.67 | <.001 |
|  | Subgroup^a^ | -0.14 | 0.78 | 0.86 | -1.48; 1.76 |  |

*^a^ Reference category=general population*

***Table 14.2.ii: Meta-regression – treatment type***

| **K** | **Variable** | **Coefficient** | **SE** | **p-value** | **95% CI** | **Test for Residual Heterogeneity** |
| --- | --- | --- | --- | --- | --- | --- |
| 23 | High intensity^a^ | -0.91 | 0.53 | 0.11 | -1.26; 0.21 | <.001 |
|  | Specialist^a^ | -1.08 | 0.55 | 0.07 | -2.22; 0.07 |  |

*^a^ Reference category=any treatment*

***Table 14.2.iii: Meta-regression – publication year***

| **K** | **Variable** | **Coefficient** | **SE** | **p-value** | **95% CI** | **Test for Residual Heterogeneity** |
| --- | --- | --- | --- | --- | --- | --- |
| 23 | Year^a^ | 0.10 | 0.10 | 0.292 | -0.09; 0.30 | <.001 |

*^a^ Reference group=2013*

***Table 14.2.iv: Meta-regression – country***

| **K** | **Variable** | **Coefficient** | **SE** | **p-value** | **95% CI** | **Test for Residual Heterogeneity** |
| --- | --- | --- | --- | --- | --- | --- |
| 23 | Canada^a^ | 1.20 | 1.36 | 0.39 | -1.62; 4.03 | <.001 |
|  | USA^a^ | 1.62 | 1.05 | 0.14 | -0.57; 3.81 |  |

*^a^ Reference group=Australia*

***Table 14.2.v: Meta-regression – treatment type***

| **K** | **Variable** | **Coefficient** | **SE** | **p-value** | **95% CI** | **Test for Residual Heterogeneity** |
| --- | --- | --- | --- | --- | --- | --- |
| 23 | Off campus^a^ | 0.91 | 0.43 | 0.05 | 0.003; 1.81 | <.001 |
|  | On campus^a^ | 1.83 | 0.48 | 0.001 | 0.83; 2.83 |  |

*^a^ Reference category=either on or off campus*

***Table 14.2.vi: Meta-regression – time-period***

| **K** | **Variable** | **Coefficient** | **SE** | **p-value** | **95% CI** | **Test for Residual Heterogeneity** |
| --- | --- | --- | --- | --- | --- | --- |
| 23 | Months^a^ | -0.01 | 0.01 | 0.15 | -0.01; 0.01 | <.001 |

*^a^ Reference category=12 months*

**Appendix S15: Specific outpatient service use analyses**

**15.1: Subgroup analyses**

***Table 15.1.i: Subgroup analysis – population group***

| **K** | **Variable** | **Proportion** | **95% CI** | *Ι* *^2^_level 2_* | *Ι* *^2^_level 3_* | *p*subgroup |
| --- | --- | --- | --- | --- | --- | --- |
| 6 | General | 0.20 | 0.08; 0.42 | 64.29% | 31.29% | 0.547 |
| 9 | At risk | 0.27 | 0.13; 0.49 |  |  |  |
| 22 | Subgroup | 0.16 | 0.09; 0.27 |  |  |  |

***Table 15.1.ii: Subgroup analysis – treatment type***

| **K** | **Variable** | **Proportion** | **95% CI** | *Ι* *^2^_level 2_* | *Ι* *^2^_level 3_* | *p*subgroup |
| --- | --- | --- | --- | --- | --- | --- |
| 4 | Any | 0.41 | 0.29; 0.55 | 64.29% | 31.2% | <0.001 |
| 17 | High intensity | 0.17 | 0.12; 0.25 |  |  |  |
| 12 | Low intensity | 0.22 | 0.14; 0.32 |  |  |  |
| 4 | Specialist | 0.08 | 0.05; 0.13 |  |  |  |

***Table 15.1.iii: Subgroup analysis – country***

| **K** | **Variable** | **Proportion** | **95% CI** | *Ι* *^2^_level 2_* | *Ι* *^2^_level 3_* | *p*subgroup |
| --- | --- | --- | --- | --- | --- | --- |
| 2 | Canada | 0.28 | 0.04; 0.78 | 64.29% | 31.3% | 0.07 |
| 4 | Brazil | 0.17 | 0.04; 0.53 |  |  |  |
| 2 | Ethiopia | 0.10 | 0.02; 0.45 |  |  |  |
| 29 | USA | 0.20 | 0.13; 0.31 |  |  |  |

***Table 15.1.iv: Subgroup analysis – service location***

| **K** | **Variable** | **Proportion** | **95% CI** | *Ι* *^2^_level 2_* | *Ι* *^2^_level 3_* | *p*subgroup |
| --- | --- | --- | --- | --- | --- | --- |
| 25 | Either | 0.24 | 0.16; 0.35 | 64.29% | 31.29% | <0.001 |
| 3 | Off-campus | 0.17 | 0.05; 0.46 |  |  |  |
| 9 | On-campus | 0.11 | 0.07; 0.18 |  |  |  |

***Table 15.1.v: Subgroup analysis – time-period***

| **K** | **Variable** | **Proportion** | **95% CI** | *Ι^2^_level 2_* | *Ι^2^_level 2_* | *p*subgroup |
| --- | --- | --- | --- | --- | --- | --- |
| 4 | Current | 0.17 | 0.03; 0.56 | 64.29% | 31.29% | 0.81 |
| 2 | Two weeks | 0.10 | 0.02; 0.47 |  |  |  |
| 4 | Two months | 0.15 | 0.03; 0.51 |  |  |  |
| 8 | Twelve months | 0.15 | 0.05; 0.36 |  |  |  |
| 5 | While at university | 0.20 | 0.06; 0.49 |  |  |  |
| 14 | Ever | 0.27 | 0.14; 0.48 |  |  |  |

**15.2: Meta-regression**

***Table 15.2.i: Meta-regression – population group***

| **K** | **Variable** | **Coefficient** | **SE** | **p-value** | **95% CI** | **Test for Residual Heterogeneity** |
| --- | --- | --- | --- | --- | --- | --- |
| 37 | At risk^a^ | 0.39 | 0.71 | 0.58 | -1.04; 1.83 | <.001 |
|  | Subgroup^a^ | -0.23 | 0.61 | 0.71 | -1.49; 1.03 |  |

*^a^ Reference category=general population*

***Table 15.2.ii: Meta-regression – treatment type***

| **K** | **Variable** | **Coefficient** | **SE** | **p-value** | **95% CI** | **Test for Residual Heterogeneity** |
| --- | --- | --- | --- | --- | --- | --- |
| 37 | High intensity^a^ | -1.21 | 0.34 | 0.001 | -1.91; -0.51 | <.001 |
|  | Low intensity^a^ | -0.94 | 0.11 | <.001 | -1.17; -0.71 |  |
|  | Specialist^a^ | -2.06 | 0.37 | <.001 | -2.81; -1.32 |  |

*^a^ Reference category=any treatment*

***Table 15.2.iii: Meta-regression – publication year***

| **K** | **Variable** | **Coefficient** | **SE** | **p-value** | **95% CI** | **Test for Residual Heterogeneity** |
| --- | --- | --- | --- | --- | --- | --- |
| 37 | Year^a^ | 0.021 | 0.06 | 0.70 | -0.09; 0.13 | <.001 |

*^a^ Reference group=2007*

***Table 15.2.iv: Meta-regression – country***

| **K** | **Variable** | **Coefficient** | **SE** | **p-value** | **95% CI** | **Test for Residual Heterogeneity** |
| --- | --- | --- | --- | --- | --- | --- |
| 37 | Canada | 0.63 | 1.36 | 0.65 | -2.14; 3.40 | <.001 |
|  | Ethiopia^a^ | -0.58 | 1.26 | 0.65 | -3.15; 1.98 |  |
|  | USA^a^ | 0.88 | 0.76 | 0.82 | -1.58; 1.99 |  |

*^a^ Reference group=Brazil*

***Table 15.2.v: Meta-regression – service location***

| **K** | **Variable** | **Coefficient** | **SE** | **p-value** | **95% CI** | **Test for Residual Heterogeneity** |
| --- | --- | --- | --- | --- | --- | --- |
| 37 | Off campus^a^ | -0.46 | 0.71 | 0.52 | -1.89; 0.98 | <.001 |
|  | On campus^a^ | -0.93 | 0.11 | <.001 | -1.15; -0.71 |  |

*^a^ Reference category=either on or off campus*

***Table 15.2.vi: Meta-regression – time-period***

| **K** | **Variable** | **Coefficient** | **SE** | **p-value** | **95% CI** | **Test for Residual Heterogeneity** |
| --- | --- | --- | --- | --- | --- | --- |
| 37 | Months^a^ | 0.01 | 0.01 | 0.089 | -0.00; 0.01 | <.001 |

*^a^ Reference category=0.5 months*

**Appendix S16: Sensitivity analyses - specific service use (multiple DESDE categories)**

**16.1: Subgroup analyses**

***Table 16.1.i: Subgroup analysis – treatment type excluding outliers and influential cases***

| **K** | **Variable** | **Proportion** | **95% CI** | *Ι* *^2^_level 2_* | *Ι* *^2^_level 3_* | *p*subgroup |
| --- | --- | --- | --- | --- | --- | --- |
| 4 | Any | 0.17 | 0.05; 0.42 | 2.6% | 91.9% | 0.945 |
| 6 | High intensity | 0.16 | 0.08; 0.30 |  |  |  |
| 4 | Specialist | 0.15 | 0.08; 0.28 |  |  |  |

***Table 16.1.ii: Subgroup analysis – service location excluding outliers and influential cases***

| **K** | **Variable** | **Proportion** | **95% CI** | *Ι* *^2^_level 2_* | *Ι* *^2^_level 3_* | *p*subgroup |
| --- | --- | --- | --- | --- | --- | --- |
| 12 | Either | 0.14 | 0.09; 0.21 | 2.6% | 91.9% | 0.006 |
| 2 | Off-campus | 0.14 | 0.07; 0.29 |  |  |  |
| 1 | On-campus | 0.59 | 0.29; 0.84 |  |  |  |

***Table 16.1.iii: Subgroup analysis – treatment type excluding lifetime use***

| **K** | **Variable** | **Proportion** | **95% CI** | *Ι* *^2^_level 2_* | *Ι* *^2^_level 3_* | *p*subgroup |
| --- | --- | --- | --- | --- | --- | --- |
| 5 | Any | 0.37 | 0.09; 0.78 | 28.9% | 68.1% | 0.489 |
| 4 | High intensity | 0.17 | 0.03; 0.58 |  |  |  |
| 1 | Specialist | 0.08 | 0.01; 0.82 |  |  |  |

***Table 16.1.iv: Subgroup analysis – service location excluding lifetime use***

| **K** | **Variable** | **Proportion** | **95% CI** | *Ι* *^2^_level 2_* | *Ι* *^2^_level 3_* | *p*subgroup |
| --- | --- | --- | --- | --- | --- | --- |
| 3 | Either | 0.07 | 0.02; 0.22 | 28.9% | 68.1% | 0.02 |
| 4 | Off-campus | 0.31 | 0.13; 0.57 |  |  |  |
| 2 | On-campus | 0.59 | 0.28; 0.84 |  |  |  |

**16.2: Meta-regression**

***Table 16.2.i: Meta-regression – treatment type excluding outliers and influential cases***

| **K** | **Variable** | **Coefficient** | **SE** | **p-value** | **95% CI** | **Test for Residual Heterogeneity** |
| --- | --- | --- | --- | --- | --- | --- |
| 15 | High intensity^a^ | -0.04 | 0.68 | 0.95 | -1.53; 1.45 | <.001 |
|  | Specialist^a^ | 0.13 | 0.68 | 0.86 | -1.62; 1.36 |  |

*^a^ Reference category=any treatment*

***Table 16.2.ii: Meta-regression – service location excluding outliers and influential cases***

| **K** | **Variable** | **Coefficient** | **SE** | **p-value** | **95% CI** | **Test for Residual Heterogeneity** |
| --- | --- | --- | --- | --- | --- | --- |
| 15 | Off campus^a^ | 0.01 | 0.46 | 0.11 | -1.51; 0.17 | <.001 |
|  | On campus | 2.17 | 0.62 | 0.01 | 0.83; 3.52 |  |

*^a^ Reference category=either on or off campus*

***Table 16.2.iii: Meta-regression – treatment type excluding lifetime use***

| **K** | **Variable** | **Coefficient** | **SE** | **p-value** | **95% CI** | **Test for Residual Heterogeneity** |
| --- | --- | --- | --- | --- | --- | --- |
| 10 | High intensity^a^ | -1.06 | 1.11 | 0.37 | -3.67; 1.56 | <.001 |
|  | Specialist^a^ | -1.91 | 1.60 | 0.27 | -5.69; 1.88 |  |

*^a^ Reference category=any treatment*

***Table 16.2.iv: Meta-regression – service location excluding lifetime use***

| **K** | **Variable** | **Coefficient** | **SE** | **p-value** | **95% CI** | **Test for Residual Heterogeneity** |
| --- | --- | --- | --- | --- | --- | --- |
| 10 | Off campus^a^ | 1.81 | 0.73 | 0.04 | 0.09; 3.54 | <.001 |
|  | On campus^a^ | 2.99 | 0.79 | 0.007 | 1.14; 4.85 |  |

*^a^ Reference category=either on or off campus*

**Appendix S7: Sensitivity analyses - specific outpatient service use**

**17.1: Subgroup analyses**

***Table 17.1.i: Subgroup analysis – treatment type excluding outliers and influential cases***

| **K** | **Variable** | **Proportion** | **95% CI** | *Ι* *^2^_level 2_* | *Ι* *^2^_level 3_* | *p*subgroup |
| --- | --- | --- | --- | --- | --- | --- |
| 1 | Any | 0.14 | 0.05; 0.34 | 100% | 0% | 0.87 |
| 11 | High intensity | 0.19 | 0.16; 0.22 |  |  |  |
| 6 | Low intensity | 0.19 | 0.17; 0.22 |  |  |  |
| 1 | Specialist | 0.20 | 0.14; 0.27 |  |  |  |

***Table 17.1.ii: Subgroup analysis – service location excluding outliers and influential cases***

| **K** | **Variable** | **Proportion** | **95% CI** | *Ι* *^2^_level 2_* | *Ι* *^2^_level 3_* | *p*subgroup |
| --- | --- | --- | --- | --- | --- | --- |
| 11 | Either | 0.18 | 0.15; 0.23 | 100% | 0% | 0.67 |
| 3 | Off-campus | 0.17 | 0.10; 0.27 |  |  |  |
| 5 | On-campus | 0.20 | 0.17; 0.23 |  |  |  |

***Table 17.1.iii: Subgroup analysis – treatment type excluding low-quality studies***

| **K** | **Variable** | **Proportion** | **95% CI** | *Ι* *^2^_level 2_* | *Ι* *^2^_level 3_* | *p*subgroup |
| --- | --- | --- | --- | --- | --- | --- |
| 2 | Any | 0.50 | 0.35; 0.65 | 49.5% | 47.6% | <.001 |
| 11 | High intensity | 0.16 | 0.10; 0.24 |  |  |  |
| 8 | Low intensity | 0.27 | 0.17; 0.40 |  |  |  |
| 4 | Specialist | 0.07 | 0.04; 0.13 |  |  |  |

***Table 17.1.iv: Subgroup analysis – service location excluding low-quality studies***

| **K** | **Variable** | **Proportion** | **95% CI** | *Ι* *^2^_level 2_* | *Ι* *^2^_level 3_* | *p*subgroup |
| --- | --- | --- | --- | --- | --- | --- |
| 14 | Either | 0.27 | 0.15; 0.43 | 49.5% | 47.6% | <.001 |
| 2 | Off-campus | 0.16 | 0.03; 0.57 |  |  |  |
| 9 | On-campus | 0.13 | 0.06; 0.23 |  |  |  |

***Table 17.1.v: Subgroup analysis – treatment type excluding lifetime use***

| **K** | **Variable** | **Proportion** | **95% CI** | *Ι* *^2^_level 2_* | *Ι* *^2^_level 3_* | *p*subgroup |
| --- | --- | --- | --- | --- | --- | --- |
| 3 | Any | 0.26 | 0.01; 0.96 | 49.8% | 44.3% | 0.33 |
| 14 | High intensity | 0.17 | 0.12; 0.22 |  |  |  |
| 3 | Low intensity | 0.24 | 0.11; 0.45 |  |  |  |
| 3 | Specialist |  |  |  |  |  |

***Table 17.1.vi: Subgroup analysis – service location excluding lifetime use***

| **K** | **Variable** | **Proportion** | **95% CI** | *Ι* *^2^_level 2_* | *Ι* *^2^_level 3_* | *p*subgroup |
| --- | --- | --- | --- | --- | --- | --- |
| 14 | Either | 0.14 | 0.06; 0.30 | 49.8% | 44.3% | 0.92 |
| 3 | Off-campus | 0.18 | 0.06; 0.45 |  |  |  |
| 6 | On-campus | 0.16 | 0.04; 0.48 |  |  |  |

**17.2: Meta-regression**

***Table 17.2.i: Meta-regression – treatment type excluding outliers and influential cases***

| **K** | **Variable** | **Coefficient** | **SE** | **p-value** | **95% CI** | **Test for Residual Heterogeneity** |
| --- | --- | --- | --- | --- | --- | --- |
| 19 | High intensity^a^ | 0.36 | 0.56 | 0.53 | -0.83; 1.55 | 0.873 |
|  | Low intensity^a^ | 0.41 | 0.55 | 0.48 | -0.78; 1.59 |  |
|  | Specialist^a^ | 0.43 | 0.58 | 0.47 | -0.81; 1.66 |  |

*^a^ Reference category=any treatment*

***Table 17.2.ii: Meta-regression – service location excluding outliers and influential cases***

| **K** | **Variable** | **Coefficient** | **SE** | **p-value** | **95% CI** | **Test for Residual Heterogeneity** |
| --- | --- | --- | --- | --- | --- | --- |
| 19 | Off campus^a^ | -0.09 | 0.29 | 0.78 | -0.71; 0.55 | 0.90 |
|  | On campus^a^ | 0.10 | 0.13 | 0.46 | -0.18; 0.37 |  |

*^a^ Reference category=either on or off campus*

***Table 17.2.iii: Meta-regression – treatment type excluding low-quality studies***

| **K** | **Variable** | **Coefficient** | **SE** | **p-value** | **95% CI** | **Test for Residual Heterogeneity** |
| --- | --- | --- | --- | --- | --- | --- |
| 25 | High intensity^a^ | -1.67 | 0.36 | <.001 | -2.41; -0.91 | <.001 |
|  | Low intensity^a^ | -0.99 | 0.11 | <.001 | -1.22; -0.76 |  |
|  | Specialist^a^ | -2.51 | 0.38 | <.001 | -3.30; -1.73 |  |

*^a^ Reference category=any treatment*

***Table 17.2.iv: Meta-regression – service location excluding low-quality studies***

| **K** | **Variable** | **Coefficient** | **SE** | **p-value** | **95% CI** | **Test for Residual Heterogeneity** |
| --- | --- | --- | --- | --- | --- | --- |
| 25 | Off campus^a^ | -0.66 | 0.94 | 0.50 | -2.61; 1.30 | <.001 |
|  | On campus^a^ | -0.94 | 0.11 | <.001 | -1.17; -0.71 |  |

*^a^ Reference category=either on or off campus*

***Table 17.2.v: Meta-regression – treatment type excluding lifetime use***

| **K** | **Variable** | **Coefficient** | **SE** | **p-value** | **95% CI** | **Test for Residual Heterogeneity** |
| --- | --- | --- | --- | --- | --- | --- |
| 23 | High intensity^a^ | -0.38 | 0.61 | -0.63 | 0.54; -1.65 | <.001 |
|  | Low intensity^a^ | -0.24 | 0.74 | 0.33 | 0.75; -1.78 |  |
|  | Specialist^a^ | -1.26 | 0.63 | 0.06 | -2.56; 0.05 |  |

*^a^ Reference category=any treatment*

***Table 17.2.vi: Meta-regression – service location excluding lifetime use***

| **K** | **Variable** | **Coefficient** | **SE** | **p-value** | **95% CI** | **Test for Residual Heterogeneity** |
| --- | --- | --- | --- | --- | --- | --- |
| 23 | Off-campus^a^ | 0.18 | 0.79 | 0.83 | -1.48; 1.83 | <.001 |
|  | On-campus^a^ | 0.31 | 0.75 | 0.69 | -1.26; 1.88 |  |

*^a^ Reference category=either on or off campus*

**References**

1. Page MJ, McKenzie JE, Bossuyt PM, Boutron I, Hoffmann TC, Mulrow CD, et al. The PRISMA 2020 statement: an updated guideline for reporting systematic reviews. BMJ. 2021;372:n71
2. Munn Z, Moola S, Lisy K, Riitano D, Tufanaru C. Methodological guidance for systematic reviews of observational epidemiological studies reporting prevalence and cumulative incidence data. Int J Evid Based Healthc. 2015;13(3):147-53.
3. Salvador-Carulla, L., Romero, C., Poole, M., Ruiz, M. for the eDESDE-LTC Group. eDESDE-LTC: Instrument-mapping tree [Internet]. Jerez (Spain): PSICOST and Telnet; 2011. Available from: http://www.edesdeproject.eu ISBN (Requested)
4. Salvador-Carulla L, Poole M, Gonzalez-Caballero JL, Romero C, Salinas JA, Lagares-Franco CM, et al. Development and usefulness of an instrument for the standard description and comparison of services for disabilities (DESDE). Acta Psychiatrica Scandinavica. 2006;114(s432):19-28.
